# Autoantibodies against type I IFNs in humans with alternative NF-κB pathway deficiency

**DOI:** 10.1038/s41586-023-06717-x

**Published:** 2023-11-08

**Authors:** Tom Le Voyer, Audrey V. Parent, Xian Liu, Axel Cederholm, Adrian Gervais, Jérémie Rosain, Tina Nguyen, Malena Perez Lorenzo, Elze Rackaityte, Darawan Rinchai, Peng Zhang, Lucy Bizien, Gonca Hancioglu, Pascale Ghillani-Dalbin, Jean-Luc Charuel, Quentin Philippot, Mame Sokhna Gueye, Majistor Raj Luxman Maglorius Renkilaraj, Masato Ogishi, Camille Soudée, Mélanie Migaud, Flore Rozenberg, Mana Momenilandi, Quentin Riller, Luisa Imberti, Ottavia M. Delmonte, Gabriele Müller, Baerbel Keller, Julio Orrego, William Alexander Franco Gallego, Tamar Rubin, Melike Emiroglu, Nima Parvaneh, Daniel Eriksson, Maribel Aranda-Guillen, David I. Berrios, Linda Vong, Constance H. Katelaris, Peter Mustillo, Johannes Raedler, Jonathan Bohlen, Jale Bengi Celik, Camila Astudillo, Sarah Winter, NF-κB Consortium, Stéphanie Boisson-Dupuis, Eric Oksenhendler, Satoshi Okada, Oana Caluseriu, Mathilde Valeria Ursini, Eric Ballot, Geoffroy Lafarge, Tomas Freiberger, Carlos A. Arango-Franco, Romain Levy, COVID Human Genetic Effort, Catriona McLean, Aurélien Guffroy, Joseph L. DeRisi, David Yu, Corey Miller, Yi Feng, Audrey Guichard, Vivien Béziat, Jacinta Bustamante, Qiang Pan-Hammarström, Yu Zhang, Lindsey B. Rosen, Steve M. Holland, Marita Bosticardo, Heather Kenney, Riccardo Castagnoli, Charlotte A. Slade, Kaan Boztuğ, Nizar Mahlaoui, Sylvain Latour, Roshini S. Abraham, Vassilios Lougaris, Fabian Hauck, Anna Sediva, Faranaz Atschekzei, Georgios Sogkas, M. Cecilia Poli, Mary A. Slatter, Boaz Palterer, Michael D. Keller, Alberto Pinzon-Charry, Anna Sullivan, Luke Droney, Daniel Suan, Melanie Wong, Alisa Kane, Hannah Hu, Cindy Ma, Hana Grombiříková, Peter Ciznar, Ilan Dalal, Nathalie Aladjidi, Miguel Hie, Estibaliz Lazaro, Jose Franco, Sevgi Keles, Marion Malphettes, Marlene Pasquet, Maria Elena Maccari, Andrea Meinhardt, Aydan Ikinciogullari, Mohammad Shahrooei, Fatih Celmeli, Patrick Frosk, Christopher C. Goodnow, Paul E. Gray, Alexandre Belot, Hye Sun Kuehn, Sergio D. Rosenzweig, Makoto Miyara, Francesco Licciardi, Amélie Servettaz, Vincent Barlogis, Guillaume Le Guenno, Vera-Maria Herrmann, Taco Kuijpers, Grégoire Ducoux, Françoise Sarrot-Reynauld, Catharina Schuetz, Charlotte Cunningham-Rundles, Frédéric Rieux-Laucat, Stuart G. Tangye, Cristina Sobacchi, Rainer Doffinger, Klaus Warnatz, Bodo Grimbacher, Claire Fieschi, Laureline Berteloot, Vanessa L. Bryant, Sophie Trouillet Assant, Helen Su, Benedicte Neven, Laurent Abel, Qian Zhang, Bertrand Boisson, Aurélie Cobat, Emmanuelle Jouanguy, Olle Kampe, Paul Bastard, Chaim M. Roifman, Nils Landegren, Luigi D. Notarangelo, Mark S. Anderson, Jean-Laurent Casanova, Anne Puel

**Affiliations:** 1 Laboratory of Human Genetics of Infectious Diseases, Necker Branch, INSERM UMR1163, Paris, France; 2 Paris Cité University, Imagine Institute, Paris, France; 3 Diabetes Center, University of California, San Francisco, San Francisco, CA USA; 4 Science for Life Laboratory, Department of Medical Biochemistry and Microbiology, Uppsala University, Uppsala, Sweden; 5 Study Center for Immunodeficiencies, Necker Hospital for Sick Children, Paris, France; 6 Garvan Institute of Medical Research, Sydney, New South Wales Australia; 7 School of Clinical Medicine, UNSW Medicine & Health, Darlinghurst, New South Wales Australia; 8 Department of Biochemistry and Biophysics, University of California San Francisco, San Francisco, CA USA; 9 St. Giles Laboratory of Human Genetics of Infectious Diseases, Rockefeller Branch, The Rockefeller University, New York, NY USA; 10 Division of Pediatric Allergy and Immunology, Ondokuz Mayıs University Faculty of Medicine, Samsun, Turkey; 11 Department of Immunology, AP-HP, Pitié-Salpêtrière Hospital, Paris, France; 12 Virology, Cochin-Saint-Vincent de Paul Hospital, University of Paris, Paris, France; 13 Laboratory of Immunogenetics of Pediatric Autoimmune Diseases, Paris Cité University, Imagine Institute, INSERM UMR1163, Paris, France; 14 Section of Microbiology, University of Brescia, Brescia, Italy; 15 Laboratory of Clinical Immunology and Microbiology, Division of Intramural Research, National Institute of Allergy and Infectious Diseases, National Institutes of Health, Bethesda, MD USA; 16 Institute for Immunodeficiency, Center for Chronic Immunodeficiencies, Medical Center—University Hospital Freiburg, and Faculty of Medicine, Albert-Ludwigs-University, Freiburg, Germany; 17 Department of Rheumatology and Clinical Immunology, Medical Center—University of Freiburg, Faculty of Medicine, University of Freiburg, Freiburg, Germany; 18 Center for Chronic Immunodeficiency (CCI), Medical Center—University of Freiburg, Faculty of Medicine, University of Freiburg, Freiburg, Germany; 19 Primary Immunodeficiencies Group, Department of Microbiology and Parasitology, School of Medicine, University of Antioquia, Medellín, Colombia; 20 Division of Pediatric Clinical Immunology and Allergy, Department of Pediatrics and Child Health, University of Manitoba, Winnipeg, Manitoba Canada; 21 Department of Pediatric Infectious Diseases, Faculty of Medicine, Selcuk University, Konya, Turkey; 22 Division of Allergy and Clinical Immunology, Department of Pediatrics, Tehran University of Medical Sciences, Tehran, Iran; 23 Department of Clinical Genetics, Uppsala University Hospital, Uppsala, Sweden; 24 Department of Immunology, Genetics and Pathology, Section of Clinical Genetics, Uppsala University and University Hospital, Uppsala, Sweden; 25 Center for Molecular Medicine, Department of Medicine (Solna), Karolinska Institute, Stockholm, Sweden; 26 Division of Immunology and Allergy, Department of Paediatrics, Hospital for Sick Children and University of Toronto, Toronto, Ontario Canada; 27 The Canadian Centre for Primary Immunodeficiency and The Jeffrey Modell Research Laboratory for the Diagnosis of Primary Immunodeficiency, The Hospital for Sick Children, Toronto, Ontario Canada; 28 Immunology and Allergy, University of Western Sydney and Campbelltown Hospital, Campbelltown, New South Wales Australia; 29 Division of Allergy and Immunology, Nationwide Children’s Hospital, The Ohio State University College of Medicine, Columbus, OH USA; 30 Division of Pediatric Immunology and Rheumatology, Department of Pediatrics, Dr. von Hauner Children’s Hospital, University Hospital, Ludwig-Maximilians-Universität München, Munich, Germany; 31 Department of Anesthesiology and Reanimation, Selcuk University Faculty of Medicine, Konya, Turkey; 32 Hospital de Niños Roberto del Río, Santiago, Chile; 33 Department of Pediatrics, Facultad de Medicina Clinica Alemana Universidad del Desarrollo, Santiago, Chile; 34 Laboratory of Lymphocyte Activation and Susceptibility to EBV, Paris Cité University, Imagine Institute, Inserm UMR1163, Paris, France; 35 Department of Anatomical Pathology, The Alfred Hospital, Prahran, Victoria Australia; 36 Department of Clinical Immunology and Internal Medicine, National Reference Center for Autoimmune Diseases, Strasbourg University Hospital, Strasbourg, France; 37 Chan Zuckerberg Biohub, San Francisco, CA USA; 38 Joint Unit Hospices Civils de Lyon—BioMérieux, Lyon, France; 39 Department of Biosciences and Nutrition, Karolinska Institutet, Stockholm, Sweden; 40 Division of Immunology, Department of Medical Biochemistry and Biophysics, Karolinska Institutet, Stockholm, Sweden; 41 NIAID Clinical Genomics Program, NIH, Laboratory of Clinical Immunology and Microbiology, Division of Intramural Research, NIAID, NIH, Bethesda, MD USA; 42 Pediatric Unit, Department of Clinical, Surgical, Diagnostic, and Pediatric Sciences, University of Pavia, Pavia, Italy; 43 Pediatric Clinic, Fondazione IRCCS Policlinico San Matteo, Pavia, Italy; 44 Immunology Division, Walter and Eliza Hall Institute, Melbourne, Victoria Australia; 45 Dept Medical Biology, University of Melbourne, Victoria Parkville, Australia; 46 Dept Clinical Immunology and Allergy, The Royal Melbourne Hospital, Parkville, Australia; 47 CeMM Research Center for Molecular Medicine of the Austrian Academy of Sciences, Vienna, Austria; 48 Department of Pediatrics and Adolescent Medicine, Medical University of Vienna, Vienna, Austria; 49 Anna Children’s Cancer Research Institute, Vienna, Austria; 50 Anna Children’s Hospital, Vienna, Austria; 51 French National Reference Center for Primary Immunodeficiencies (CEREDIH), Necker-Enfants University Hospital, Assistance Publique-Hôpitaux de Paris (AP-HP), Paris, France; 52 Department of Pediatric Immunology, Hematology and Rheumatology, Necker-Enfants Malades Hospital, AP-HP, Paris, France; 53 Department of Pathology and Laboratory Medicine, Nationwide Children’s Hospital, Columbus, OH USA; 54 Department of Clinical and Experimental Sciences, Pediatrics Clinic and Institute for Molecular Medicine A. Nocivelli, University of Brescia ASST-Spedali Civili di Brescia, Brescia, Italy; 55 Department of Immunology, Second Faculty of Medicine Charles University and Motol University Hospital, Prague, Czech Republic; 56 Department of Rheumatology and Immunology, Hannover Medical School, Hannover, Germany; 57 Children’s Haemopoietic Stem Cell Transplant Unit, Great North Children’s Hospital, Newcastle-upon-Tyne Hospital NHS Foundation Trust, Newcastle upon Tyne, UK; 58 Allergy and Clinical Immunology, Department of Experimental and Clinical Medicine, University of Florence, Florence, Italy; 59 Division of Allergy and Immunology, Children’s National Medical Center, Washington, DC USA; 60 Clinical Immunogenomics Research Consortium Australasia (CIRCA), Darlinghurst, New South Wales Australia; 61 Immunology and Allergy, Queensland Children’s Hospital, South Brisbane, Queensland Australia; 62 Westmead Clinical School, University of Sydney, Sydney, New South Wales Australia; 63 Faculty of Medicine, University of Sydney, Sydney, New South Wales Australia; 64 South Western Sydney Clinical School, Faculty of Medicine and Health, UNSW Sydney, Sydney, New South Wales Australia; 65 Department of Immunology, Allergy and HIV, St Vincent’s Hospital, Sydney, New South Wales Australia; 66 Centre for Cardiovascular Surgery and Transplantation, Medical Faculty, Masaryk University, Brno, Czech Republic; 67 Department of Paediatrics, Faculty of Medicine, Comenius University Bratislava, Bratislava, Slovakia; 68 Pediatric Department, E. Wolfson Medical Center, Tel Aviv University, Tel Aviv, Israel; 69 Pediatric Oncology Hematology Unit, University Hospital, Plurithématique CIC (CICP), Centre d’Investigation Clinique (CIC) 1401, Bordeaux, France; 70 Internal Medicine Department, Pitié-Salpêtrière Hospital, Paris, France; 71 Department of Internal Medicine & Infectious Diseases, Bordeaux Hospital University, Bordeaux, France; 72 Division of Pediatric Allergy and Immunology, Meram Medical Faculty, Necmettin Erbakan University, Konya, Turkey; 73 Clinical Immunology Department, Saint Louis Hospital, Paris, France; 74 Department of Pediatric Hematology, Toulouse University Hospital, Toulouse, France; 75 Division of Pediatric Hematology and Oncology, Department of Pediatrics and Adolescent Medicine, Medical Center-University of Freiburg, Faculty of Medicine, University of Freiburg, Freiburg, Germany; 76 Department of Pediatric Hematology, Oncology and Immunodeficiencies, University Children’s Hospital Gießen, Giessen, Germany; 77 Department of Pediatric Immunology and Allergy, Ankara University School of Medicine, Ankara, Turkey; 78 Dr. Shahrooei Lab, Tehran, Iran; 79 Clinical and Diagnostic Immunology, Department of Microbiology, Immunology, and Transplantation, KU Leuven, Leuven, Belgium; 80 Department of Allergy and Immunology, University of Medical Science, Antalya Education and Research Hospital, Antalya, Turkey; 81 Department of Biochemistry and Medical Genetics, Rady Faculty of Health Sciences, University of Manitoba, Winnipeg, Manitoba Canada; 82 Immunology and Infectious Diseases, Sydney Children’s Hospital Randwick, Western Sydney University, Campbelltown, New South Wales Australia; 83 CNRS UMR 5308, ENS, UCBL, Lyon, France; 84 National Reference Center for Rheumatic, Autoimmune and Systemic Diseases in Children (RAISE), Lyon, France; 85 Immunopathology Federation LIFE, Hospices Civils de Lyon, Lyon, France; 86 Immunology Service, Department of Laboratory Medicine, Clinical Center, National Institutes of Health, Bethesda, MD USA; 87 Centre d’Immunologie et des Maladies Infectieuses (CIMI), Sorbonne Université, INSERM U1135, Paris, France; 88 Department of Pediatrics and Public Health, Università degli Studi di Torino, Turin, Italy; 89 Internal Medicine, Clinical Immunology and Infectious Diseases Department, University Hospital Center, Reims, France; 90 IRMAIC EA 7509, URCA, Reims, France; 91 CHU Marseille, Hôpital La Timone, Service d’Hémato-oncologie Pédiatrique, Assistance Publique-Hôpitaux de Marseille, Marseille, France; 92 Centre Hospitalier Universitaire Estaing, Clermont-Ferrand, France; 93 Institute of Human Genetics, University of Leipzig Medical Center, Leipzig, Germany; 94 Department of Pediatric Immunology, Rheumatology and Infectious Diseases, Emma Children’s Hospital, Amsterdam University Medical Center, University of Amsterdam, Amsterdam, The Netherlands; 95 Department of Internal Medicine, Edouard Herriot Hospital, Lyon, France; 96 Department of Internal Medicine, Hôpital Michallon, CHU de Grenoble Alpes, Grenoble, France; 97 Department of Pediatrics, Universitätsklinikum Carl Gustav Carus, Technische Universität Dresden, Dresden, Germany; 98 Departments of Medicine and Pediatrics, Mount Sinai School of Medicine, New York, NY USA; 99 IRCCS Humanitas Research Hospital, Rozzano, Italy; 100 CNR-IRGB, Milan Unit, Milan, Italy; 101 Department of Clinical Biochemistry and Immunology, Addenbrooke’s Hospital, Cambridge, UK; 102 Paris Cité University, Paris, France; 103 Pediatric Radiology Department, Assistance Publique-Hôpitaux de Paris (AP-HP), Necker Hospital for Sick Children, Paris, France; 104 CIRI (Centre International de Recherche en Infectiologie), Université de Lyon, Université Claude Bernard Lyon 1, INSERM U1111, CNRS, UMR5308, ENS Lyon, Université Jean Monnet de Saint-Etienne, Lyon, France; 105 Department of Endocrinology, Metabolism and Diabetes, Karolinska University Hospital, Stockholm, Sweden; 106 Department of Medicine, University of California, San Francisco, San Francisco, CA USA; 107 Howard Hughes Medical Institute, New York, NY USA; 108 Department of Pediatrics, Necker Hospital for Sick Children, Paris, France; 109 Department of Pediatrics, Graduate School of Biomedical and Health Sciences, Hiroshima University, Hiroshima, Japan; 110 Department of Medical Genetics, Faculty of Medicine and Dentistry, College of Health Sciences, Women’s and Children’s Research Institute, University of Alberta, Edmonton, Alberta Canada; 111 Institute of Genetics and Biophysics ‘Adriano Buzzati-Traverso’ (CNR), Naples, Italy; 112 Département d’immunologie biologique, Hôpital Saint-Antoine-DMU BioGeMH-AP-HP, Sorbonne Université, Paris, France; 113 San Raffaele Telethon Institute for Gene Therapy, IRCCS Ospedale San Raffaele, and Vita Salute San Raffaele University, Milan, Italy; 114 Immunology Research Lab, Department of Pediatrics, College of Medicine, King Saud University, Riyadh, Saudi Arabia; 115 Department of Genetics and Bioinformatics, Dasman Diabetes Institute, Dasman, Kuwait; 116 Laboratory of Immunobiology, Center for Clinical, Experimental Surgery and Translational Research, Biomedical Research Foundation of the Academy of Athens, Athens, Greece; 117 Department of Microbiology and Parasitology, School of Medicine, University of Antioquia, Medellín, Colombia; 118 School of Microbiology, University of Antioquia UdeA, Medellín, Colombia; 119 The Genetics Institute, Tel Aviv Sourasky Medical Center and Sackler Faculty of Medicine, Tel Aviv University, Tel Aviv, Israel; 120 Shupyk National Medical Academy for Postgraduate Education, Kiev, Ukraine; 121 Neonatology and Neonatal Intensive Care Unit, Fondazione IRCCS Policlinico “San Matteo”, Pavia, Italy; 122 Clinical Immunology Unit, Department of Pediatric Infectious Disease, CHU Ibn Rushd and LICIA, Laboratoire d’Immunologie Clinique, Inflammation et Allergie, Faculty of Medicine and Pharmacy, Hassan II University, Casablanca, Morocco; 123 SciLifeLab, Department Of Women’s and Children’s Health, Karolinska Institutet, Stockholm, Sweden; 124 Department of Medicine, Center for Hematology and Regenerative Medicine, Karolinska Institutet, Stockholm, Sweden; 125 Clinical Genomics, IRCCS San Raffaele Scientific Institute and Vita-Salute San Raffaele University, Milan, Italy; 126 Murdoch Children’s Research Institute and Department of Paediatrics, University of Melbourne, Melbourne, Victoria Australia; 127 Immunology Division, Genetics Department, Hospital Universitari Vall d’Hebron, Vall d’Hebron Research Institute, Vall d’Hebron Barcelona Hospital Campus, UAB, Barcelona, Spain; 128 Department of Immunology, Institute of Biomedical Sciences, University of São Paulo, São Paulo, Brazil; 129 School of Life Sciences, Ecole Polytechnique Fédérale de Lausanne, Lausanne, Switzerland; 130 Precision Medicine Unit, Lausanne University Hospital and University of Lausanne, Lausanne, Switzerland; 131 Research Unit, Hospital Universitario Nuestra Señora de Candelaria, Santa Cruz de Tenerife, CIBER de Enfermedades Respiratorias (CIBERES), Instituto de Salud Carlos III, Madrid, Spain; 132 Genomics Division, Instituto Tecnológico y de Energías Renovables (ITER), Santa Cruz de Tenerife, Spain; 133 Department of Clinical Sciences, University Fernando Pessoa Canarias, Las Palmas de Gran Canaria, Spain; 134 Department of Paediatric Immunology and Pulmonology, Centre for Primary Immunodeficiency Ghent (CPIG), PID Research Laboratory, Jeffrey Modell Diagnosis, Research Centre, Ghent University Hospital, Ghent, Belgium; 135 Research Institute for Medical and Health Sciences, University of Sharjah, Sharjah, United Arab Emirates; 136 Institute for Systems Biology, Seattle, WA USA; 137 Departments of Pediatrics, Immunology and Microbiology, University of Colorado, School of Medicine, Aurora, CO USA; 138 Institute for Personalized Medicine, Icahn School of Medicine at Mount Sinai, New York, NY USA; 139 Department of Genetics and Genomic Sciences, Icahn School of Medicine at Mount Sinai, New York, NY USA; 140 Department of Medical Chemistry and Laboratory Medicine, Poznan University of Medical Sciences, Poznan, Poland; 141 Molecular Pathology, Department of Biomedicine, Institute of Biomedicine and Translational Medicine, University of Tartu, Tartu, Estonia; 142 Chang Gung University, Taoyuan County, Taiwan; 143 Shanghai Public Health Clinical Center, Fudan University, Shanghai, China; 144 Department of Paediatrics & Adolescent Medicine, The University of Hong Kong, Hong Kong, China; 145 Department of Clinical Immunology and Infectious Diseases, National Research Institute of Tuberculosis and Lung Diseases, The Clinical Tuberculosis and Epidemiology Research Center, National Research Institute of Tuberculosis and Lung Diseases (NRITLD), Masih Daneshvari Hospital, Shahid Beheshti, University of Medical Sciences, Tehran, Iran; 146 Department of Pediatrics, University Hospitals Leuven, KU Leuven, Leuven, Belgium; 147 Department of Microbiology, Immunology and Transplantation, Laboratory for Inborn Errors of Immunity, KU Leuven, Leuven, Belgium; 148 Department of Pediatrics, Columbia University Irving Medical Center, New York, NY USA; 149 Department of Biomedicine, Aarhus University, Aarhus, Denmark; 150 Laboratory of Medical Genetics, IRCCS Bambino Gesù Children’s Hospital, Rome, Italy; 151 Department of Biomedicine and Prevention, Tor Vergata University of Rome, Rome, Italy; 152 Tokyo Medical and Dental University, Tokyo, Japan; 153 Department of Molecular Biology and Genetics, Bilkent University, Bilkent, Ankara, Turkey; 154 Institute of Biomedical Research of IdiPAZ, University Hospital “La Paz”, Madrid, Spain; 155 Institut de Biomedicina de València-CSIC, CIBERNED, Unitat Mixta de Neurologia i Genètica, IIS La Fe, Vallencia, Spain; 156 Center for Discovery and Innovation, Hackensack Meridian Health, Nutley, NJ USA; 157 Faculdades Pequeno Príncipe, Instituto de Pesquisa Pelé Pequeno Príncipe, Curitiba, Brazil; 158 Neurometabolic Diseases Laboratory, Bellvitge Biomedical Research Institute (IDIBELL), L’Hospitalet de Llobregat, Barcelona, Spain; 159 Catalan Institution of Research and Advanced Studies (ICREA), Barcelona, Spain; 160 Center for Biomedical Research on Rare Diseases (CIBERER), ISCIII, Barcelona, Spain; 161 Human Evolutionary Genetics Unit, CNRS U2000, Institut Pasteur, Paris, France; Human Genomics and Evolution, Collège de France, Paris, France; 162 A*STAR Infectious Disease Labs, Agency for Science, Technology and Research, Singapore, Singapore; 163 Lee Kong Chian School of Medicine, Nanyang Technology University, Singapore, Singapore; 164 University Hospital St Marina, Varna, Bulgaria; 165 Department of Immunology, University Hospital of Gran Canaria Dr. Negrín, Canarian Health System, Las Palmas de Gran Canaria, Spain; 166 Department of Paediatric Infectious Diseases and Virology, Imperial College London, London, UK; 167 Centre for Paediatrics and Child Health, Faculty of Medicine, Imperial College London, London, UK; 168 Adult Immunodeficiency Unit, Infectious Diseases, Inflammation Center, University of Helsinki and Helsinki University Hospital, Helsinki, Finland; 169 Rare Diseases Center and Pediatric Research Center, Children’s Hospital, University of Helsinki and Helsinki University Hospital, Helsinki, Finland; 170 Department of Immunology, Dmitry Rogachev National Medical Research Center of Pediatric Hematology, Oncology and Immunology, Moscow, Russia; 171 Pediatric Infectious Diseases and Immunodeficiencies Unit, Vall d’Hebron Barcelona Hospital Campus, Barcelona, Spain; 172 Department of Biosciences, Biotechnology and Biopharmaceutics, University of Bari A. Moro, Bari, Italy; 173 Department of Internal Medicine II, Medical University of Innsbruck, Innsbruck, Austria; 174 Al Jalila Children’s Hospital, Dubai, UAE; 175 Laboratory of Genomes & Cell Biology of Disease, INSERM U944, CNRS UMR 7212, Université de Paris, Institut de Recherche Saint-Louis, Hôpital Saint-Louis, Paris, France; 176 Sorbonne Université, Inserm, Centre d’Immunologie et des Maladies Infectieuses-Paris (CIMI PARIS), Assistance Publique-Hôpitaux de Paris (AP-HP) Hôpital Pitié-Salpêtrière, Paris, France; 177 Departments of Medical Genetics & Histology and Embryology, Faculty of Medicine, Bursa Uludag University, Bursa, Turkey; 178 Department of Translational Medicine, Health Sciences Institude, Bursa Uludağ University, Bursa, Turkey; 179 Etablissement Francais du Sang, La Plaine-St Denis, France, UMR 1098 RIGHT, Inserm EFS, Université de Franche-Comté, Besançon, France; 180 BC Children’s Hospital, The University of British Columbia, Vancouver, British Columbia Canada; 181 Centre for Precision Therapeutics, Genetics & Genomic Medicine Centre, NeuroGen Children’s Healthcare and Lecturer, Holy Family Red Crescent Medical College Dhaka, Dhaka, Bangladesh; 182 College of Medicine, Mohammed Bin Rashid University of Medicine and Health Sciences, Dubai, UAE; 183 Cellular Intelligence (Ci) Lab, GenomeArc, Toronto, Ontario Canada; 184 Department of Neurology, Amsterdam Neuroscience, Amsterdam University Medical Center, University of Amsterdam, Amsterdam, The Netherlands; 185 Biosciences Institute, University of São Paulo, São Paulo, Brazil; 186 Department of Medicine, Division of Infectious Diseases, McGill University Health Centre, Montréal, Quebec Canada; 187 Infectious Disease Susceptibility Program, Research Institute, McGill University Health Centre, Montréal, Quebec Canada; 188 Department of Pediatric Pneumology, Immunology and Intensive Care, Charité Universitätsmedizin, Berlin University Hospital Center, Berlin, Germany; 189 Department of Immunology, Labor Berlin, Berlin, Germany; 190 Berlin Institutes of Health (BIH), Berlin-Brandenburg Center for Regenerative Therapies, Berlin, Germany; 191 Department of General Internal Medicine, Medical Intensive Care Unit, University Hospitals Leuven, Leuven, Belgium

**Keywords:** Immunogenetics, Infectious diseases

## Abstract

Patients with autoimmune polyendocrinopathy syndrome type 1 (APS-1) caused by autosomal recessive AIRE deficiency produce autoantibodies that neutralize type I interferons (IFNs)^1,2^, conferring a predisposition to life-threatening COVID-19 pneumonia^3^. Here we report that patients with autosomal recessive NIK or RELB deficiency, or a specific type of autosomal-dominant NF-κB2 deficiency, also have neutralizing autoantibodies against type I IFNs and are at higher risk of getting life-threatening COVID-19 pneumonia. In patients with autosomal-dominant NF-κB2 deficiency, these autoantibodies are found only in individuals who are heterozygous for variants associated with both transcription (p52 activity) loss of function (LOF) due to impaired p100 processing to generate p52, and regulatory (IκBδ activity) gain of function (GOF) due to the accumulation of unprocessed p100, therefore increasing the inhibitory activity of IκBδ (hereafter, p52^LOF^/IκBδ^GOF^). By contrast, neutralizing autoantibodies against type I IFNs are not found in individuals who are heterozygous for *NFKB2* variants causing haploinsufficiency of p100 and p52 (hereafter, p52^LOF^/IκBδ^LOF^) or gain-of-function of p52 (hereafter, p52^GOF^/IκBδ^LOF^). In contrast to patients with APS-1, patients with disorders of NIK, RELB or NF-κB2 have very few tissue-specific autoantibodies. However, their thymuses have an abnormal structure, with few AIRE-expressing medullary thymic epithelial cells. Human inborn errors of the alternative NF-κB pathway impair the development of AIRE-expressing medullary thymic epithelial cells, thereby underlying the production of autoantibodies against type I IFNs and predisposition to viral diseases.

## Abstract (web summary)

Inborn errors of the alternative NF-κB pathway in humans impair the development of AIRE-expressing medullary thymic epithelial cells, thereby underlying the production of autoantibodies against type I IFNs and predisposition to viral diseases

## Main

Autoantibodies neutralizing type I IFNs (AAN-I-IFNs) have been reported in patients treated with type I IFNs, systemic lupus erythematosus (SLE), thymoma or myasthenia gravis^4^. These autoantibodies were widely thought to be clinically silent, with the notable exception of a 77-year-old woman who had such antibodies and disseminated shingles, reported in 1981^5,6^. Nearly 40 years later, we showed that pre-existing neutralizing AAN-I-IFNs underlie at least 15% of cases of life-threatening COVID-19 pneumonia^4,7–11^. These autoantibodies were also found to underlie severe adverse reactions to yellow fever live-attenuated viral vaccine^12^, influenza pneumonia^13^, MERS pneumonia^14^ and West Nile virus encephalitis^15^. AAN-I-IFNs underlie clinical phenocopies of inborn errors of type I IFN immunity, as the same viral diseases have been reported in patients with autosomal-recessive IFNAR1 or IFNAR2 deficiency^4,9,11^. These autoantibodies block cell-protective antiviral effects of type I IFNs in vitro^8,12,13,15^ and impair the induction of IFN-stimulated genes (ISGs) in peripheral blood mononuclear cells and nasal mucosae infected with SARS-CoV-2 ex vivo^7,16,17^. Finally, these autoantibodies are also present in the general population, with the prevalence sharply increasing in individuals over 70 years of age, thereby contributing to the age-related increase in the risk of severe COVID-19^7,10^.

Notably, the production of AAN-I-IFNs can be driven by monogenic inborn errors of immunity (IEIs). These IEIs include autosomal-recessive APS-1, which is caused by germline biallelic deleterious variants of *AIRE*; immunodysregulation polyendocrinopathy enteropathy X-linked (IPEX) syndrome, caused by deleterious hemizygous variants of *FOXP3*; and combined immunodeficiency due to biallelic hypomorphic *RAG1* or *RAG2* variants^4^. All of these IEIs affect T cell thymic selection, in a T-cell-intrinsic or -extrinsic manner. AIRE deficiency impairs the expression of tissue-specific antigens in medullary thymic epithelial cells (mTECs), enabling autoreactive T cells to escape^18,19^. FOXP3 deficiency impairs the development of thymic regulatory T (T_reg_) cells, whereas hypomorphic variants of *RAG1* or *RAG2*, which restrict TCR diversity, also have an effect on thymic architecture and the development of mTECs^20–22^. The disruption of self-tolerance in the thymus therefore seems to underlie the production of AAN-I-IFNs. Patients with APS-1 display severe multiorgan autoimmunity with a wide range of autoantibodies against tissue-specific antigens^18^. They also frequently have neutralizing autoantibodies against IL-17A and/or IL-17F that underlie chronic mucocutaneous candidiasis, a disease that is seen in patients with inborn errors of IL-17A/F immunity^4^. Most, if not all, patients with APS-1 also produce AAN-I-IFNs in early childhood^4^, and are highly vulnerable to critical COVID-19 pneumonia^3^ and to severe varicella^23^.

In mice, the expression of the *Aire* gene in mTECs is controlled by the alternative (or non-canonical) NF-κB pathway^24–26^. Once triggered, NIK activates IKKα, leading to the phosphorylation of the full-length NF-κB2 precursor p100 (amino acids 1–900) on serine residues Ser866 and Ser870. This leads to p100 processing to generate the p52 (amino acids 1–405) active form, which preferentially dimerizes with RELB^27^. This p52–RELB heterodimer migrates to the nucleus, inducing the transcription of target genes involved in lymphoid organ development, germinal centre formation, B cell survival, maturation, homeostasis, mTEC development and osteoclastogenesis^27^. In resting cells, unprocessed cytoplasmic p100 can form high-molecular-mass complexes by homomultimerization (generating kappaBsomes) through its C-terminal IκB-like domain, thereby inhibiting the DNA-binding activity of almost all NF-κB subunits (referred to as IκBδ function)^28^. In the mouse thymus, RANK and the alternative NF-κB pathway have a crucial role in mTECs by governing self-tolerance^24,26^. Deficiencies in mouse *Traf6*, *Ikkα*, *Map3k14* (encoding NIK) or *RelB* impair mTEC development and AIRE expression in mTECs^29^. We tested the hypothesis that human inborn errors of the alternative NF-κB pathway—including autosomal-dominant NF-κB2 disorders, and autosomal-recessive RELB, IKKα and NIK deficiencies—can underlie the production of AAN-I-IFNs, thereby predisposing patients to severe viral diseases, including COVID-19 pneumonia.

## Inborn errors of the alternative NF-κB pathway

**Fig. 1:**
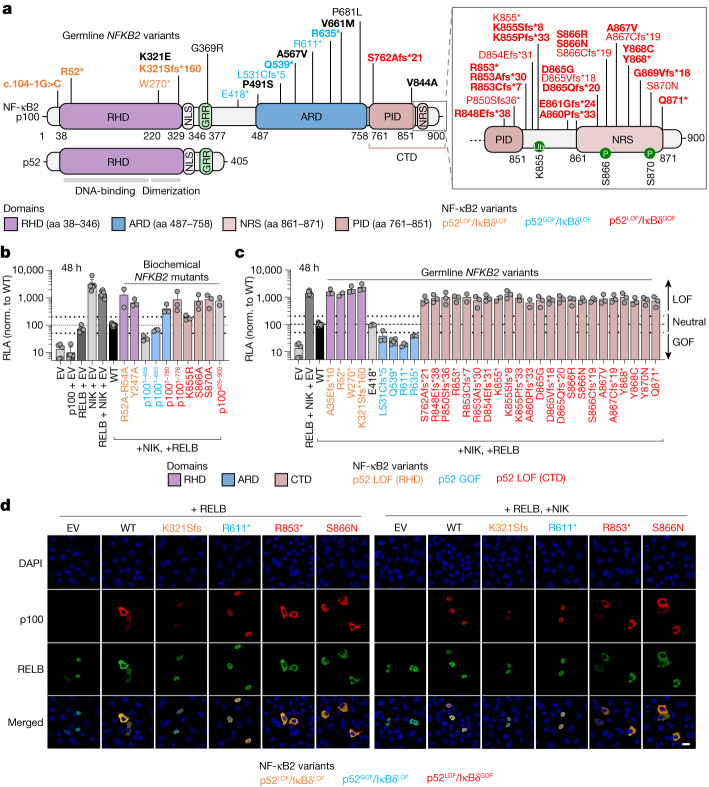
Functional testing of the *NFKB2* alleles by overexpression. **a**, Schematic of the NF-κB2 protein (p100 and p52) with the variants, identified in heterozygous patients, that were included in this study (*n* = 28 variants, shown in bold) or not included here but reported elsewhere (*n* = 13 variants). The C-terminal domain (CTD) spans amino acids (aa) 760–900. The REL-homology domain (RHD; purple), the ankyrin repeat domain (ARD; blue) and the CTD, including the processing-inhibitory domain (PID) and the NIK-responsive sequence (NRS) (brown), are shown. The *NFKB2* variants that are LOF for p52/p52 repression of κB transcriptional activity (p52 activity) and LOF for IκBδ regulatory activity (p52^LOF^/IκBδ^LOF^) are shown in orange. The variants that are GOF for p52 activity and LOF for IκBδ activity are shown in blue (p52^GOF^/IκBδ^LOF^). The variants in the CTD that are both LOF for the p52 activity and GOF for the IκBδ regulatory activity (p52^LOF^/IκBδ^GOF^) are shown in red. Neutral *NFKB2* variants are shown in black. **b**, The relative luciferase activity (RLA) of HEK293T cells transfected with a κB reporter luciferase construct (κB-luc) in the presence or absence of plasmids encoding NIK, RELB and/or p100/NF-κB2 WT or biochemical p100/NF-κB2 mutants reported in previous studies, normalized (norm.) to WT p100/NF-κB2, after 48 h of transfection. Data are mean ± s.d. from three independent experiments. EV, empty vector. **c**, The RLA of HEK293T cells transfected with a κB-luc vector, in the presence of plasmids encoding NIK, RELB and p100/NF-κB2 WT or the *NFKB2* variants included in this study or reported in previous studies, at 48 h after transfection. Data are mean ± s.d. from three independent experiments. **d**, Subcellular localization of the WT or the NF-κB2 variants used for cotransfection with RELB without (left) or with (right) NIK, as determined by confocal microscopy analysis of HeLa cells. The nuclei were stained with DAPI; p100 and RELB were detected using antibodies recognizing their N-terminal domains. Data shown are representative of two independent experiments. Scale bar, 20 μm.

**Fig. 2:**
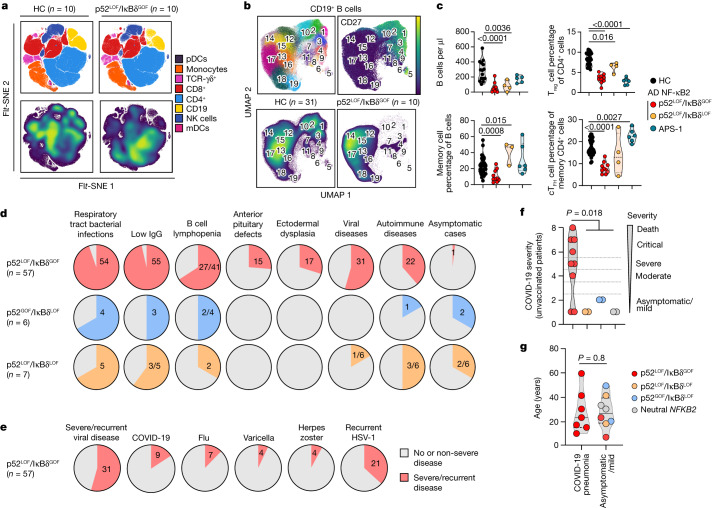
Distinctive immunological and clinical phenotype of patients with p52^LOF^/IκBδ^GOF^ heterozygous variants. **a**, FFT-accelerated interpolation-based (FI) *t*-distributed stochastic neighbour embedding (*t*-SNE) analysis of concatenated whole-blood samples from ten patients with p52^LOF^/IκBδ^GOF^ variants, and ten age-matched healthy control individuals (HC), based on cytometry by time of flight (CyTOF) data. *t*-SNE analysis is not shown for the patients with p52^LOF^/IκBδ^LOF^ variants (*n* = 4) or APS-1 (*n* = 6) owing to their lower number. NK, natural killer cells; mDCs and pDCs, myeloid and plasmacytoid dendritic cells, respectively. **b**, Uniform manifold approximation and projection (UMAP)-based unsupervised clustering analysis of CD19^+^ B cells from a concatenated group of 10 patients with p52^LOF^/IκBδ^GOF^ variants and 31 age-matched controls (HC), with a heat map showing the mean levels of the surface markers included in the clustering defining 19 distinct metaclusters, CD27 marker expression and the metacluster distribution in healthy control individuals and patients with p52^LOF^/IκBδ^GOF^ variants. **c**, The number of B cells and the proportions of memory B cells, T_reg_ cells and circulating T_FH_ (cT_FH_) cells in patients with a p52^LOF^/IκBδ^GOF^ variant (*n* = 10, red dots, except for the B cell numbers, showing only patients above 6 years of age, *n* = 9), age-matched controls (*n* = 27, black dots), patients with a p52^LOF^/IκBδ^LOF^ variant (*n *= 4, orange dots) and patients with APS-1 (*n* = 6, green dots). Statistical comparisons were performed using two-tailed Mann–Whitney *U*-tests. AD, autosomal dominant. **d**, The proportion and number of patients with p52^LOF^/IκBδ^GOF^ (*n* = 57), p52^GOF^/IκBδ^LOF^ (*n* = 6) or p52^LOF^/IκBδ^LOF^ (*n* = 7, including 4 reported here and 3 previously reported^56^) NF-κB2 variants with their corresponding clinical manifestations. **e**, The proportion and number of patients with severe/recurrent (red shape) or no/non-severe (grey shape) viral diseases among the 57 patients with p52^LOF^/IκBδ^GOF^ NF-κB2 variants. **f**, COVID-19 severity scale for unvaccinated patients with a p52^LOF^/IκBδ^GOF^ (red dots, *n* = 9), p52^LOF^/IκBδ^LOF^ (orange dots, *n* = 2), p52^GOF^/IκBδ^LOF^ (blue dots, *n* = 2) or neutral (grey dots, *n* = 2) NF-κB2 variant. Statistical comparisons were performed using two-tailed Mann–Whitney *U*-tests. **g**, Age at the COVID-19 episode in unvaccinated patients with a p52^LOF^/IκBδ^GOF^ (red dots, *n* = 9), p52^LOF^/IκBδ^LOF^ (orange dots, *n* = 2), p52^GOF^/IκBδ^LOF^ (blue dots, *n* = 2) or neutral (grey dots, *n* = 2) NF-κB2 variant, as a function of COVID-19 severity. Statistical comparisons were performed using two-tailed Mann–Whitney *U*-tests.

**Extended Data Fig. 1:**
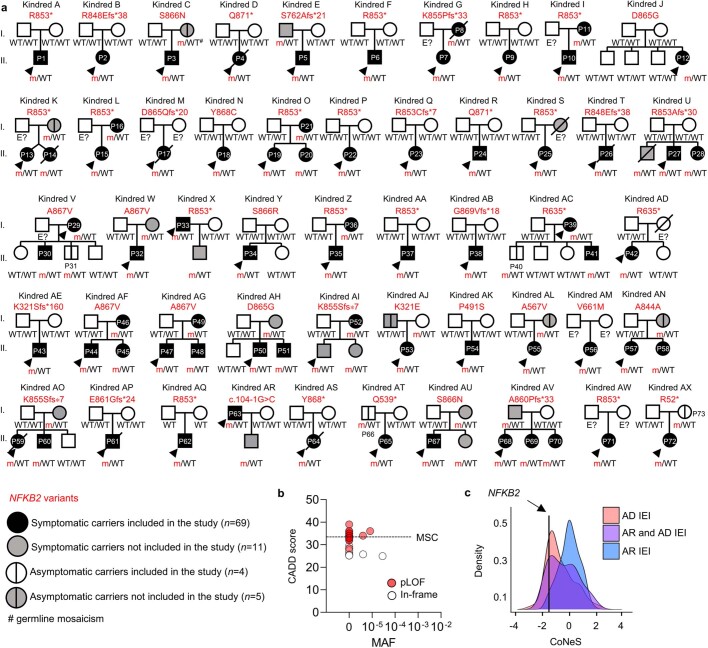
Pedigrees of the 73 patients studied carrying heterozygous *NFKB2* variants. **(a)** Pedigrees of the patients heterozygous for rare variants of *NFKB2*. Generations are indicated by Roman numerals (I–II), and each symptomatic carrier included in the study, represented by a black symbol, is indicated as P followed by an Arabic numeral (P1–P73). Grey symbols represent relatives who are symptomatic carriers but for whom no material was available for this study. A vertical bar, within a white or grey symbol, indicates an asymptomatic carrier included or not included (due to a lack of available material), respectively in the study; an arrow indicates the index case; a black diagonal line indicates that the individual is deceased. “E?” indicates individuals of unknown genotype. (**b**) CADD-MAF (combined annotation-dependent depletion-minor allele frequency) graph of the rare or private *NFKB2* variants (*n* = 28) from the 73 patients recruited. The red and white dots represent pLOF and missense heterozygous *NFKB2* variants, respectively. Each score was calculated with CADD version 1.6. The dashed line represents the mutation significance (MSC) cutoff threshold of 33 for *NFKB2*. **(C)** CoNeS score of the *NFKB2* gene.

**Extended Data Fig. 2:**
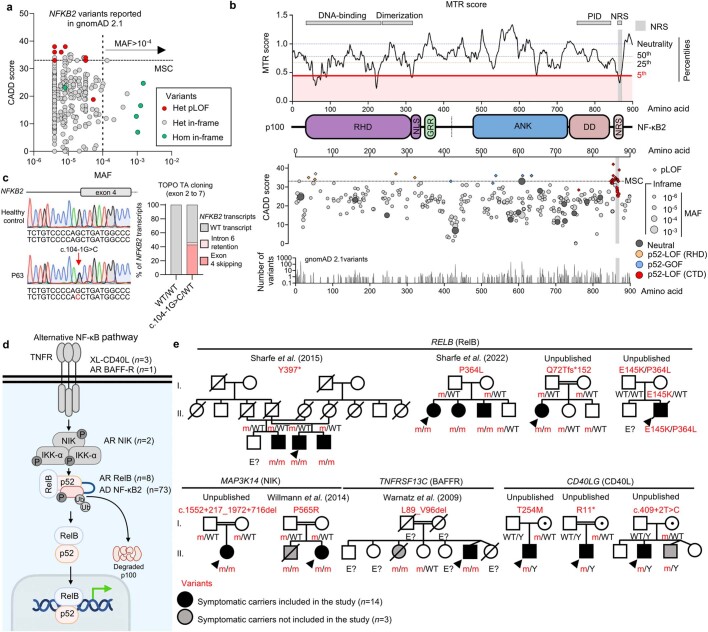
Population genetics and constraint metrics of the *NFKB2* gene, and pedigrees of the patients with inborn errors of RelB, NIK, BAFFR and CD40L. **(a)** CADD-MAF graph for the *NFKB2* variants reported in the gnomAD v2.2.1. The red and grey dots represent monoallelic pLOF and heterozygous in-frame (missense and indel) variants, respectively. The green dots represent homozygous missense variants. The horizontal dashed line represents the mutation significance (MSC) cutoff threshold of 33 for *NFKB2*. (**b**) Genomic constrained coding regions across *NFKB2*, as estimated by the missense tolerance ratio (MTR) score evaluating region-specific intolerance to missense variants. A score <1.0 indicates a lower-than-expected ratio of missense to synonymous variants in the gnomAD v2.0 dataset for the 21 bp window surrounding an amino-acid residue. The horizontal-coloured dashed lines represent the percentiles for the most missense-depleted regions of *NFKB2*. The NIK-responsive sequence (NRS) is within the 5th percentile for the most missense-depleted regions for *NFKB2*. The lower graph shows the distribution of the heterozygous *NFKB2* variants reported in gnomAD 2.1.1 and from the patients reported in this study, by location within the protein and CADD score. (**c**) Electropherograms showing the c.104-1 G > C/WT essential splice-site variant carried in the heterozygous state of P63 and a healthy donor (left) and the proportion of transcripts identified by sequencing 100 colonies from TOPO cloning with cDNA from PCR products corresponding to a region spanning exon 2 to 7 in P63 or a healthy donor. (**d**) Representation of the alternative NF-κB pathway and the patients included. **(e)** Pedigrees and variants of patients with inborn errors of RelB, NIK, BAFFR and CD40L. A dot within a white symbol indicates an asymptomatic carrier; an arrow indicates the index case; a black diagonal line indicates a deceased individual. “E?” indicates individuals of unknown genotype.

**Extended Data Fig. 3:**
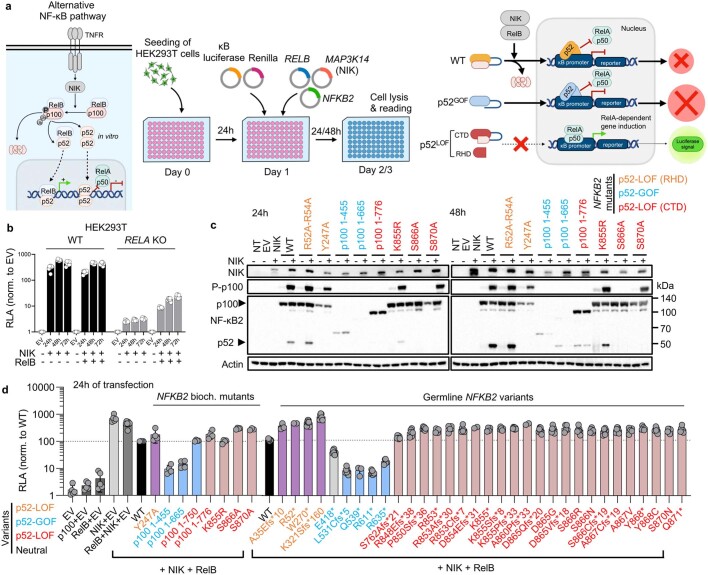
Functional testing of *NFKB2* variants by overexpression. **(a)** Schematic representation of the alternative NF-κB pathway and the function of the p52/RelB and p52/p52 heterodimers (left panel); a graphical overview of the luciferase assay for testing the p52 function of the *NFKB2* variants (middle panel); and a schematic representation of the functional consequences of the WT, p52^GOF^ and p52^LOF^ variants in the luciferase assay (right panel). (**b**) Relative luciferase activity (RLA) of WT or RelA-deficient HEK293T cells transfected with a κB reporter construct (κB-luc) in the absence or presence of plasmids encoding NIK and/or RelB for 24, 48 or 72 h. Results are expressed as the RLA normalized against the value for the EV. Bars represent the mean values (± s.d.) from 3 independent experiments performed in technical duplicates. **(c)** Western blot of HEK293T cells transfected for 24 h (left panel) or 48 h (right panel) in the absence or presence of plasmids encoding NIK and the WT or previously reported biochemical NF- κB2/p100 mutants. Data representative of two independent experiment are shown. **(d)** Luciferase assay testing the NF-κB2/p100 biochemical mutants (left) or deleterious variants from patients (right), 24 h after transfection. Bars represent the mean values (± s.d.) from more than 3 independent experiments.

**Extended Data Fig. 4:**
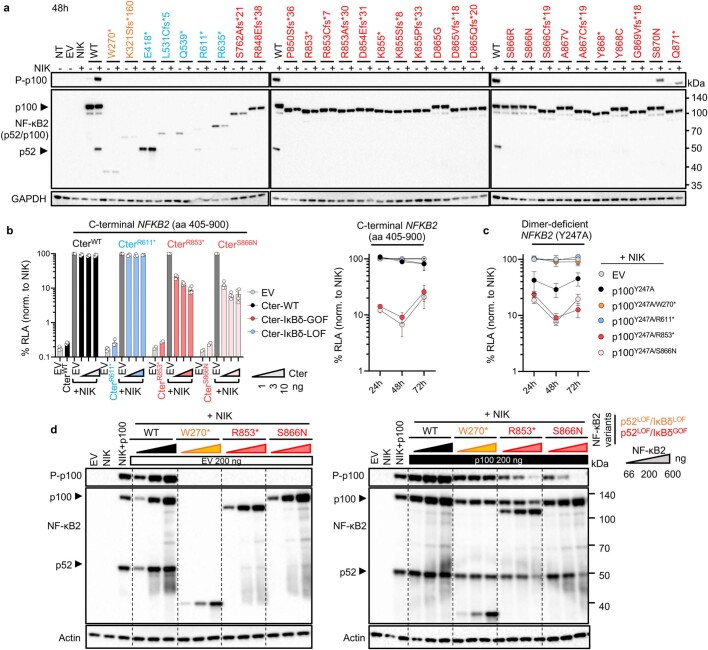
Assessments of NIK-dependent p100 processing and p100-IκBδ activity of the *NFKB2* variants by overexpression. **(a)** Western blot of HEK293T cells transfected for 48 h in the presence or absence of plasmids encoding NIK and WT or mutant NF-κB2, showing phosphorylated Y866-p100 (P-p100) levels, and p100 and p52 expression. Data representative of three independent experiments are shown. **(b)** Relative luciferase activity (RLA), indicating the p100/NF-κB2-dependent capacity to repress κB transcriptional activity for luciferase in HEK293T cells in the presence or absence of plasmids encoding NIK and various amounts of a plasmid encoding the C-terminal part of p100/NF-κB2 (Cter, aa 405-900) from WT or mutants, 48 h after transfection. The results are expressed as a percentage of the κB-luc RLA after transfection with NIK alone (left panel); the kinetic effect of transfection with NIK either alone or together with a plasmid encoding the various Cter constructs, as assessed by κB-luc transcriptional repression, from 24 to 72 h after transfection is shown in the right panel. Bars represent the mean values (± s.d.) from two independent experiments performed in duplicate. **(c)** Kinetic effect of transfection with NIK alone or together with a plasmid encoding the dimer-deficient Y247A single (p100^Y247A^) or double (p100/^Y247A/W270*^, p100^Y247A/R611*^, p100^Y247A/S866N^, or p100^Y247A/R853*^) mutants, in terms of κB transcriptional repression of luciferase activity from 24 to 72 h after transfection. Results are expressed as a percentage of the κB RLA after transfection with NIK alone. Bars represent the mean values (± s.d.) from two independent experiments performed in duplicate. (**d**) Western blot of HEK293T cells cotransfected with a plasmid encoding NIK, together with various amounts of a plasmid encoding the WT or mutant NF-κB2 variants, together with a constant amount of empty vector (left panel) or of WT *NFKB2* (right panel), showing phosphorylated p100 (P-p100), p100 and p52 expression. Data representative of three independent experiments are shown.

**Extended Data Fig. 5:**
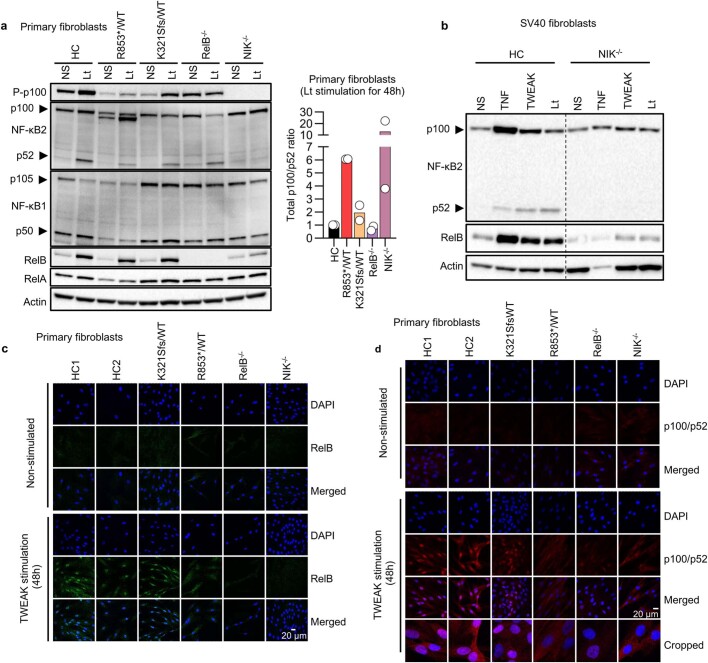
The processing-resistant *NFKB2* mutants have enhanced p100-IκBδ activity in heterozygous patients’ cells. **(a)** Western blot of P-p100, NF-κB2 (p100/p52), NF-κB1 (p105/p50), RelB, and RelA in primary fibroblasts from one healthy donor (HC), a patient with the p52^LOF^/IκBδ^GOF^ R853*/WT variant, a patient with the p52^LOF^/IκBδ^LOF^ K321Sfs/WT variant, a patient with AR complete (Q72Tfs*152/Q72Tfs*152) RelB (RelB^−/−^) or (P565R/P565R) NIK (NIK^−/−^) deficiency, with or without stimulation with LT-α1β2 (Lt) for 48 h (left panel), and a graph depicting total p100/p52 intensity ratio after Lt stimulation (right panel). Bars represent the mean values (± s.d.) from two independent experiments. **(b)** Western blot showing p100 processing into p52 and RelB induction in total cell extracts of SV40 fibroblasts from a healthy donor (HC) or a patient with AR complete NIK deficiency (NIK^−/−^) either left non stimulated (NS) or after stimulation for 48 h with TNF, TWEAK, Lt. Data representative of three independent experiments are shown. **(c)** Confocal microscopy showing the subcellular distribution of RelB in primary fibroblasts from two healthy controls (HC1, HC2), patients with a p52^LOF^/IκBδ^LOF^ K321Sfs/WT or a p52^LOF^/IκBδ^GOF^ R853*/WT NF-κB2/p100 variant, and patients with AR complete RelB (RelB^−/−^) or NIK (NIK^−/−^) deficiency, without and with stimulation with TWEAK for 48 h. Data representative of three independent experiments are shown. **(d)** Confocal microscopy showing the subcellular distribution of p100/p52 in primary fibroblasts from two healthy controls (HC1, HC2), patients with a p52^LOF^/IκBδ^LOF^ K321Sfs*/WT or a p52^LOF^/IκBδ^GOF^ R853*/WT NF-κB2/p100 variant, AR complete RelB deficiency (RelB^−/−^), or AR complete NIK deficiency (NIK^−/−^) without and with stimulation with TWEAK for 48 h, with an antibody recognizing the N-terminus of p100. Data representative of three independent experiments are shown. The bottom panels represent magnified images (cropped images).

**Extended Data Fig. 6:**
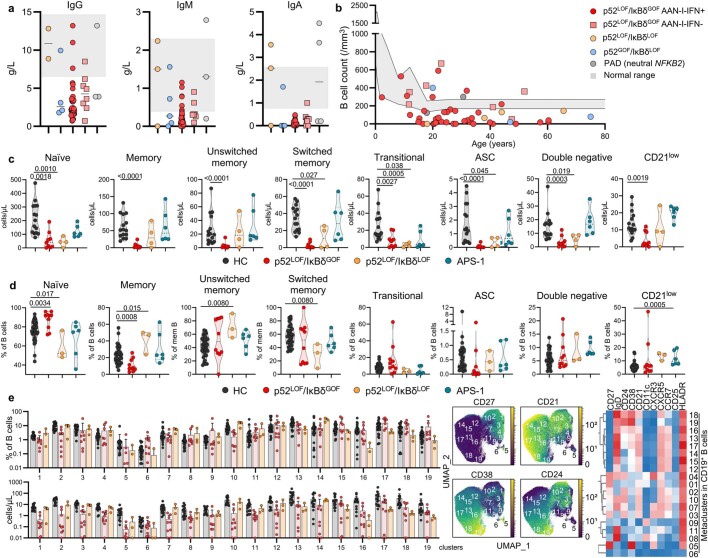
Immunoglobulin level and B cell immunophenotyping of patients with inborn errors of NF-κB2. **(a)** Immunoglobulin IgG, IgM, and IgA levels (g/L) in patients with inborn errors of NF-κB2. Normal immunoglobulin distribution corresponds to the grey area. Bars represent the median values. **(b)** B cell count across ages in patients with p52^LOF^/IκBδ^GOF^ variants with (*n* = 39, red dots) and without (*n* = 7, red squares) AAN-I-IFNs, patients with p52^LOF^/IκBδ^LOF^/WT (*n* = 3, orange dots), p52^GOF^/IκBδ^LOF^/WT (*n* = 4, blue dots), or neutral (PAD, grey dots) NF-κB2 variants. Normal B-cell count for age corresponds to the grey area. **(c)** Cell numbers among B cell subsets, as determined by CyTOF, in healthy donors (*n* = 15, black dots), patients with p52^LOF^/IκBδ^GOF^ variants aged ≥ 6 years (*n* = 9, red dots), patients with p52^LOF^/IκBδ^LOF^ variants (*n* = 4, orange dots), and APS-1 patients (*n* = 6, green dots). **(d)** Proportions of B cell subsets, as determined by CyTOF, in healthy donors (*n* = 15, black dots), patients with p52^LOF^/IκBδ^GOF^ variants (*n* = 10, red dots), patients with p52^LOF^/IκBδ^LOF^ variants (*n* = 3, orange dots), and APS-1 patients (*n* = 6, green dots). B-cell subset proportions from a patient with a p52^LOF^/IκBδ^LOF^ R52*/WT variant are not shown due to his lack of circulating B cells. **(e)** Proportions of B cell subsets and absolute counts of B cells identified in the 19 metaclusters in healthy donors (*n* = 22, black dots), patients with a p52^LOF^/IκBδ^GOF^ variant aged ≥ 6 years (*n* = 9, red dots), and patients with a p52^LOF^/IκBδ^LOF^ variant (*n* = 3, orange dots) (left panels); representation of the CD27, CD21, CD38, and CD24 markers on UMAP (middle panels); heatmap showing the mean levels of the surface markers included in the clustering defining 19 distinct metaclusters. The error bars represent the mean values (± s.d.) of each group.

We recruited an international cohort of 73 patients from 50 kindreds heterozygous for 28 different rare (minor allele frequency (MAF) < 0.0001) non-synonymous *NFKB2* variants (Extended Data Fig. 1a,b and Supplementary Table 1). Most affected individuals had a predominant phenotype of primary antibody deficiency (PAD) (62 out of 69, 89.9% of these patients). After a comprehensive functional characterization, we identified three types of autosomal-dominant inborn errors of NF-κB2, designated as p52^LOF^/IκBδ^LOF^ in 4 patients heterozygous for *NFKB2* variants causing haploinsufficiency of p100 and p52; p52^GOF^/IκBδ^LOF^ in 6 patients heterozygous for *NFKB2* variants causing GOF of p52; and p52^LOF^/IκBδ^GOF^ in 57 patients heterozygous for *NFKB2* variants that are associated with both transcriptional (p52 activity) LOF due to impaired p100 processing to generate p52, and regulatory activity (IκBδ activity) GOF due to the accumulation of unprocessed p100 (Fig. 1, Supplementary Results 1, Extended Data Figs. 2–5 and Supplementary Figs. 2–6). Six other patients carried a neutral *NFKB2* heterozygous variant (hereafter, idiopathic PAD). Among the three inborn errors of NF-κB2, only the p52^LOF^/IκBδ^GOF^ variants severely impaired the alternative NF-κB pathway activation by preventing the nuclear translocation of p52 and RELB (Fig. 1d and Supplementary Results 1). Moreover, the p52^LOF^/IκBδ^GOF^ variants also impaired the formation of p52–RELB heterodimers in patients’ heterozygous fibroblasts, in contrast to fibroblasts that are heterozygous for a p52^LOF^/IκBδ^LOF^ variant (Extended Data Fig. 5c,d). Finally, only patients heterozygous for p52^LOF^/IκBδ^GOF^ variants displayed a unique immunological phenotype associated with B cell lymphopenia and reduced T_reg_ and T_FH_ cell counts (Fig. 2a–c, Supplementary Results 2, Extended Data Fig. 6 and Supplementary Fig. 7). We also enrolled 14 patients with other inborn errors of the alternative NF-κB pathway (autosomal-recessive NIK (*n* = 2) and autosomal-recessive RELB (*n* = 8) deficiencies) or upstream receptors (autosomal-recessive BAFF (*n* = 1) or X-linked recessive CD40L (*n* = 3) deficiencies) (Extended Data Fig. 2d,e and Supplementary Table 2).

## Viral diseases of patients with p52^LOF^/IκBδ^GOF^

Whereas PAD and autoimmune diseases were reported in patients with any of the three types of autosomal-dominant NF-κB2 deficiency, ectodermal dysplasia and anterior pituitary hormone deficiencies were reported exclusively in patients carrying p52^LOF^/IκBδ^GOF^ variants (Fig. 2d and Supplementary Table 1). Severe or recurrent viral diseases were almost exclusively reported in patients carrying p52^LOF^/IκBδ^GOF^ variants (*n* = 31 out of 57, 54%) (Fig. 2d,e). This susceptibility could not be explained by immunosuppressive treatments (used in seven patients with p52^LOF^/IκBδ^GOF^ variants) (Supplementary Table 1). The main viral disease reported was recurrent mucocutaneous HSV-1 lesions (*n* = 21, 37%) (Fig. 2e). Six out of the nine unvaccinated patients and two patients with an unknown vaccination status with p52^LOF^/IκBδ^GOF^ variants developed hypoxaemic COVID-19 pneumonia (NIH scale, 5 to 8, out of 8) after infection with SARS-CoV-2. Three of these patients, aged 17, 23 and 39 years, were admitted to intensive care and two of these individuals (aged 23 and 39 years) died (Fig. 2f). One patient was hospitalized for COVID-19 pneumonia without requiring oxygen supplementation (NIH scale, 4). Eight additional unvaccinated patients developed asymptomatic disease or mild symptoms (NIH scale, 1–2) without pneumonia or hospitalization. These patients carried a p52^LOF^/IκBδ^GOF^ (*n* = 2, aged 7 and 22 years), p52^LOF^/IκBδ^LOF^ (*n* = 2, aged 17 and 41 years), p52^GOF^/IκBδ^LOF^ (*n* = 2, aged 20 and 49 years) or neutral (*n* = 2, aged 30 and 31 years) NF-κB2 variant (Fig. 2f). COVID-19 severity was not associated with age or treatment (Fig. 2g and Supplementary Table 1). Severe influenza pneumonia was reported in 7 out of the 57 patients with p52^LOF^/IκBδ^GOF^ variants (12%), five of whom required hospitalization and oxygen supplementation, including one patient with acute respiratory distress syndrome (ARDS) and encephalitis (Fig. 2e). Four patients suffered from recurrent (*n* = 1) or severe (*n* = 3) varicella (Fig. 2e). All patients with severe varicella were hospitalized, including one with encephalitis and one with severe skin disease requiring acyclovir. The other severe viral diseases observed are indicated in Supplementary Table 1. None of the patients were vaccinated with yellow fever YFV-17D live-attenuated vaccine. All eight of the patients with inborn errors of NF-κB2 who died carried a p52^LOF^/IκBδ^GOF^ variant; six died from suspected or proven viral illnesses, including two from COVID-19. Together, these findings suggest that, in contrast to patients with the other two forms of inborn errors of NF-κB2, patients with p52^LOF^/IκBδ^GOF^ variants present a distinctive syndrome that is strongly associated with the risk of developing PAD and/or a severe viral disease. Conversely, p52/p100 haploinsufficiency (p52^LOF^/IκBδ^LOF^) and GOF of p52 (p52^GOF^/IκBδ^LOF^) may underlie humoral deficiency with variable clinical and immunological penetrance, whereas these conditions do not appear to underlie ectodermal, endocrine or viral phenotypes. The milder clinical phenotype associated with these forms may account for the smaller number of patients with such defects identified.

## AAN-I-IFNs in patients with p52^LOF^/IκBδ^GOF^

**Fig. 3:**
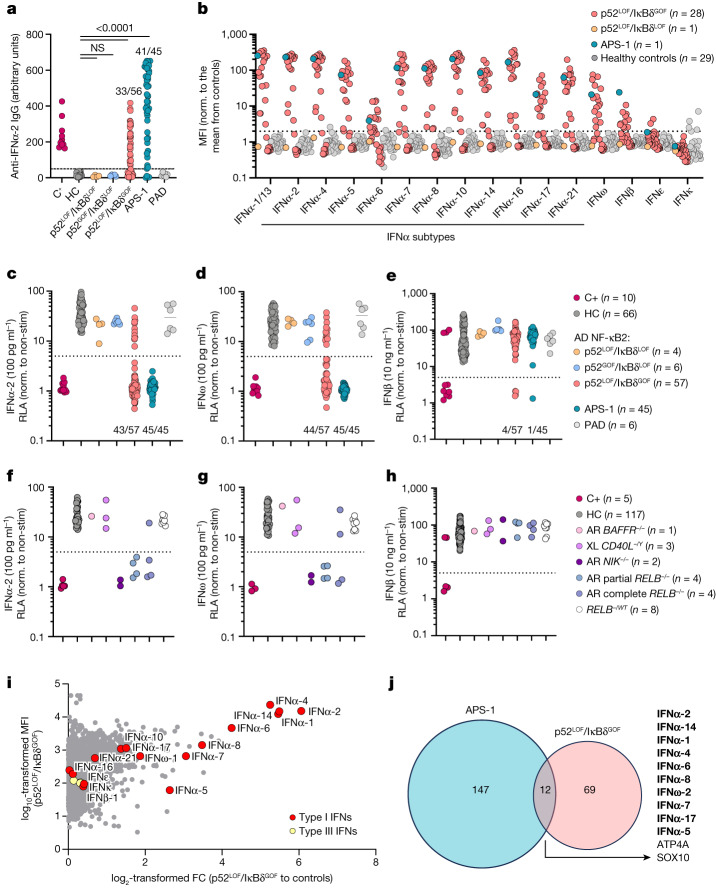
AAN-I-IFNs detected in patients heterozygous for p52^LOF^/IκBδ^GOF^ variants and patients with inborn errors of RELB or NIK. **a**, Detection of IgG autoantibodies against IFNα-2 by Gyros in patients with inborn errors of NF-κB2 with a p52^LOF^/IκBδ^LOF^ (*n* = 4), p52^GOF^/IκBδ^LOF^ (*n* = 6) or p52^LOF^/IκBδ^GOF^ (*n* = 56) variant, patients with APS-1 (*n* = 45), patients with idiopathic PAD (*n* = 6), positive control individuals with AAN-I-IFNs (C+, *n* = 10) or healthy control individuals (HC, *n* = 25). Data are the mean values from at least three independent experiments. Statistical comparisons were performed using two-tailed Mann–Whitney *U*-tests. NS, not significant. **b**, Detection, using a multiplex bead assay, of autoantibodies against the 16 type I IFNs in patients with p52^LOF^/IκBδ^GOF^ (*n* = 28) or p52^LOF^/IκBδ^LOF^ (*n* = 1) variants or with APS-1 (*n* = 1). Values are normalized to the mean fluorescence intensity (MFI) of plasma samples from healthy control individuals (*n* = 29) for each indicated cytokine. **c**–**e**, Luciferase-based neutralization assay to detect autoantibodies neutralizing 100 pg ml^−1^ IFNα-2 (**c**), IFNω (**d**) or 10 ng ml^−1^ IFNβ (**e**) in positive-control individuals (*n* = 10), healthy control individuals (*n* = 66), patients with a p52^GOF^/IκBδ^LOF^ (*n* = 6), p52^LOF^/IκBδ^LOF^ (*n* = 4) or p52^LOF^/IκBδ^GOF^ (*n* = 57) variant, patients with idiopathic PAD (*n* = 6) and patients with APS-1 (*n* = 45). Non-stim., non-stimulated. **f**–**h**, Luciferase-based neutralization assay to detect autoantibodies neutralizing 100 pg ml^−1^ IFNα-2 (**f**) or IFNω (**g**) or 10 ng ml^−1^ IFNβ (**h**) in patients with autosomal-recessive BAFFR (*n* = 1), X-linked (XL) CD40L deficiency (*n* = 3), autosomal-recessive NIK deficiency (*n* = 2), autosomal-recessive RELB partial or complete deficiency (*n* = 8) in healthy relatives heterozygous for a null or hypomorphic *RELB* allele (*n* = 8), positive control individuals (*n* = 5) or healthy control individuals (*n* = 117). All neutralization assay data are presented as the mean of at least two independent experiments. **i**, Protein microarray showing the distribution of autoantibody reactivity in plasma samples from patients carrying a p52^LOF^/IκBδ^GOF^ variant (*n* = 13). Data are represented as the fold change (FC) relative to 26 plasma samples from healthy control individuals. Data for HuProt are presented as the mean of at least two technical replicates. **j**, Representation of the global autoantigen profile of patients with APS-1 and patients with a p52^LOF^/IκBδ^GOF^ variant, with their overlap. Type I IFN autoantigens are highlighted in bold.

**Extended Data Fig. 7:**
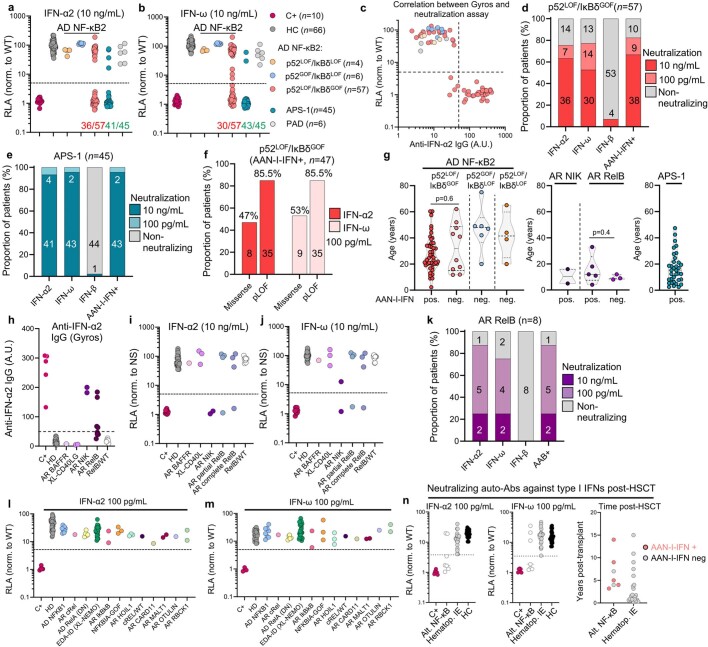
AAN-I-IFNs in patients with inborn errors of the alternative NF-κB pathway. **(a-b**) Luciferase-based neutralization assay for detecting auto-Abs neutralizing 10 ng/mL IFN-α2 (**a**) or IFN-ω (**b**) in patients with the three inborn errors of NF-κB2, APS-1 and PAD patients, positive controls (C+), and healthy controls (HC). **(c)** Correlation between the detection of auto-Abs against IFN-α2 by Gyros (*x*-axis) and results for the luciferase-based neutralization assay (*y*-axis) after stimulation with 10 ng/mL IFN-α2. The dotted line represents the cutoffs for detection (A.U. value > 50) or neutralization (induction <5). **(d-e)** Proportion of patients with auto-Abs neutralizing type I IFNs at 10 ng/mL or 100 pg/mL among patients with a p52^LOF^/IκBδ^GOF^ variant (**d**), and APS-1 patients (**e**). (**f**) Proportion of patients with AAN-I-IFNs among patients carrying a missense or pLOF p52^LOF^/IκBδ^GOF^ variant. (**g**) Age distribution of patients with the three inborn errors of NF-κB2, AR RelB or NIK deficiency, or APS-1 according to the presence or absence of AAN-I-IFNs in plasma. (**h**) Detection of IgG auto-Abs against IFN-α2 by Gyros in patients with inborn errors of NIK, RelB, BAFF and CD40L. (**i-j**) Luciferase-based neutralization assay for detecting auto-Abs neutralizing 10 ng/mL IFN-α2 (**i**) or IFN-ω (**j**) in patients with inborn errors of NIK, RelB, BAFF and CD40L. **(k)** Proportion of patients with auto-Abs neutralizing type I IFNs at 10 ng/mL or 100 pg/mL in patients with AR RelB deficiency. (**l-m**) Luciferase-based neutralization assay for detecting auto-Abs neutralizing 100 pg/mL IFN-α2 (**l**) or IFN-ω (**m**) in patients with inborn errors of the canonical NF-κB pathway. DN = dominant-negative. (**n**) Detection of auto-Abs neutralizing 100 pg/mL IFN-α2 or IFN-ω in patients with inborn errors of the alternative NF-κB pathway post-HSCT (*n* = 7) versus children with inborn errors of T-cell intrinsic immunity [(SCID, *n* = 3, CID, *n* = 1), neutrophil-intrinsic immunity (chronic granulomatous disease, CGD, *n* = 10), cytotoxicity (familial hemophagocytic lymphohistiocytosis, HLH, *n* = 3), erythrocyte function (β-thalassaemia, *n* = 3)] who underwent HSCT (Hematop. IE, *n* = 20) (left panel), with the time interval between HSCT and plasma collection (right panel).

Given the strong susceptibility of patients heterozygous for p52^LOF^/IκBδ^GOF^ to viral diseases, we assessed the presence of AAN-I-IFNs in the plasma of 73 patients heterozygous for a deleterious or neutral variant. We detected high titres (arbitrary units > 50) of anti-IFNα-2 IgG in 33 out of 56 (59%) patients with p52^LOF^/IκBδ^GOF^ variants, 41 out of 45 (91%) patients with APS-1, but none in those carrying p52^LOF^/IκBδ^LOF^ (*n* = 4) or p52^GOF^/IκBδ^LOF^ (*n* = 6) alleles, or with idiopathic PAD (*n* = 6) (Fig. 3a). Moreover, patients with p52^LOF^/IκBδ^GOF^ variants and autoantibodies against IFNα-2 also had detectable autoantibodies against most of the 11 other IFNα subtypes, IFNω and, less frequently, IFNβ, but not against IFNκ or IFNε, as evaluated in a multiplex bead assay (Fig. 3b). We next assessed the neutralization ability of patients’ plasma in the presence of high (10 ng ml^−1^) or low (100 pg ml^−1^) concentrations of IFNα-2, IFNω or IFNβ (10 ng ml^−1^). Overall, 36 out of 57 (65%), 30 out of 57 (53%) and 4 out of 57 (7%) patients with p52^LOF^/IκBδ^GOF^ variants neutralized high concentrations of IFNα-2, IFNω and IFNβ, respectively (Fig. 3e and Extended Data Fig. 7a–c), and 43 out of 57 (75%) and 44 out of 57 (77%) neutralized low concentrations of IFNα-2 or IFNω, respectively (Fig. 3c,d and Extended Data Fig. 7d). For comparison, 41 (91%), 43 (96%) and 1 (2%) out of the 45 patients with APS-1 neutralized IFNα, IFNω and IFNβ, respectively, at a concentration of 10 ng ml^−1^ (Fig. 3e and Extended Data Fig. 7a, b), and serum from all of these patients neutralized IFNα-2 and IFNω at a concentration of 100 pg ml^−1^ (Fig. 3c,d and Extended Data Fig. 7e). By contrast, none of the plasma samples from any of the patients with p52^LOF^/IκBδ^LOF^, p52^GOF^/IκBδ^LOF^ or neutral *NFKB2* variants neutralized IFNα-2, IFNω or IFNβ (at 10 ng ml^−1^ or 100 pg ml^−1^). The proportion of patients with p52^LOF^/IκBδ^GOF^ variants carrying AAN-I-IFNs was higher among those carrying pLOF variants than among those carrying missense variants but was independent of patient age at testing (*P* = 0.6) or sex (Extended Data Fig. 7f,g). In ten patients with p52^LOF^/IκBδ^GOF^ variants, no neutralizing autoantibodies against IFNα-2, IFNω or IFNβ could be detected. Seven of them carried the A867V variant (Supplementary Results 3 and Supplementary Fig. 8). In total, plasma samples from 82% (47 out of 57) of the patients with a p52^LOF^/IκBδ^GOF^ variant neutralized IFNα-2 and/or IFNω; the plasma of three of these patients neutralized only IFNα-2, whereas that of four patients neutralized only IFNω, and that of another four patients neutralized IFNα-2, IFNω and IFNβ (Extended Data Fig. 7d and Supplementary Table 4). Overall, we found a strong association between the *NFKB2* genotype (p52^LOF^/IκBδ^GOF^) and the presence of AAN-I-IFNs (Supplementary Fig. 9).

## AAN-I-IFNs in NIK or RELB deficiency

We next investigated the presence of AAN-I-IFNs in patients with other inborn errors of the alternative NF-κB pathway. AAN-I-IFNs were detected in the two patients with complete autosomal-recessive NIK deficiency. In one of these patients, the autoantibodies detected neutralized IFNα-2 and IFNω at a concentration of 10 ng ml^−1^, whereas, in the other, they neutralized IFNα-2 at 10 ng ml^−1^ and IFNω at 100 pg ml^−1^ (Fig. 3f–h, Extended Data Fig. 7h–j and Supplementary Fig. 10). AAN-I-IFNs were also detected in patients with autosomal-recessive RELB deficiency (*n* = 7 out of 8: four patients with partial and three with complete deficiency). These autoantibodies neutralized IFNα-2 and IFNω at 10 ng ml^−1^ in two patients, and IFNα-2 and/or IFNω at 100 pg ml^−1^ in five patients (Fig. 3f–h and Extended Data Fig. 7h–k). By contrast, no AAN-I-IFNs were detected in patients with autosomal-recessive BAFFR or X-linked CD40L deficiency, or in the plasma from heterozygous relatives of patients with autosomal-recessive RELB deficiency (*n* = 8) (Fig. 3f–h and Extended Data Fig. 7h–j). Finally, we tested eight patients with autosomal-dominant NF-κB1 haploinsufficiency, and 32 additional patients with deleterious mutations of 10 different canonical NF-κB pathway-related genes (*REL*, *RELA*, *IKBKB*, *IKBKG*, *NFKBIA*, *HOIL1*, *CARD11*, *MALT1*, *OTULIN* and *RBCK1*). All of the patients tested negative for AAN-I-IFNs (Extended Data Fig. 7l,m). These autoantibodies were also absent in patients with IEIs associated with defective T follicular helper (T_FH_) cell function (autosomal-dominant STAT3 deficiency, *n* = 11), low T_reg_ cell proportions (autosomal-dominant IL6ST deficiency, *n* = 10; autosomal-recessive ZNF341 deficiency, *n* = 10), or both low T_reg_ and T_FH_ cell counts (autosomal-recessive CARMIL2 deficiency, *n* = 16) (Supplementary Fig. 11). Haematopoietic stem cell transplantation (HSCT) cannot cure defects of thymic stromal cells. We therefore hypothesized that AAN-I-IFNs might appear even after transplantation. One of the four patients with autosomal-recessive complete RELB deficiency who had undergone HSCT had neutralizing AAN-I-IFNs before transplantation (at the age of 2 years). Neutralizing AAN-I-IFNs were detected in post-transplant samples from two out of the three other patients with RELB deficiency (Q72Tfs*152 and Y397*, 6 and 2.5 years after HSCT, respectively), whereas no such autoantibodies were detected in a patient with autosomal-recessive c-REL deficiency over a period of 7 years after HSCT, or in children with inborn errors of T-cell-intrinsic or neutrophil-intrinsic immunity or of erythrocyte function (*n* = 20), up to 15 years after transplantion (Supplementary Table 5 and Extended Data Fig. 7n). These autoantibodies were also detected in the plasma of patients with autosomal-recessive complete NIK deficiency (*n* = 2 out of 2, 3 and 7 years after HSCT), or with a p52^LOF^/IκBδ^GOF^ variant (*n* = 1 out of 1, 14 years after HSCT), for whom the available plasma samples were collected exclusively after transplantation (Supplementary Table 5). These results suggest that inborn errors of RELB, NIK and NF-κB2 from the alternative NF-κB pathway underlie the development of AAN-I-IFNs, even after HSCT, whereas defects of the canonical NF-κB pathway do not. Effective functioning of the alternative NF-κB pathway in thymic stromal cells therefore appears to be essential to prevent the generation of AAN-I-IFNs.

## Autoantibody profile of patients with p52^LOF^/IκBδ^GOF^

**Extended Data Fig. 8:**
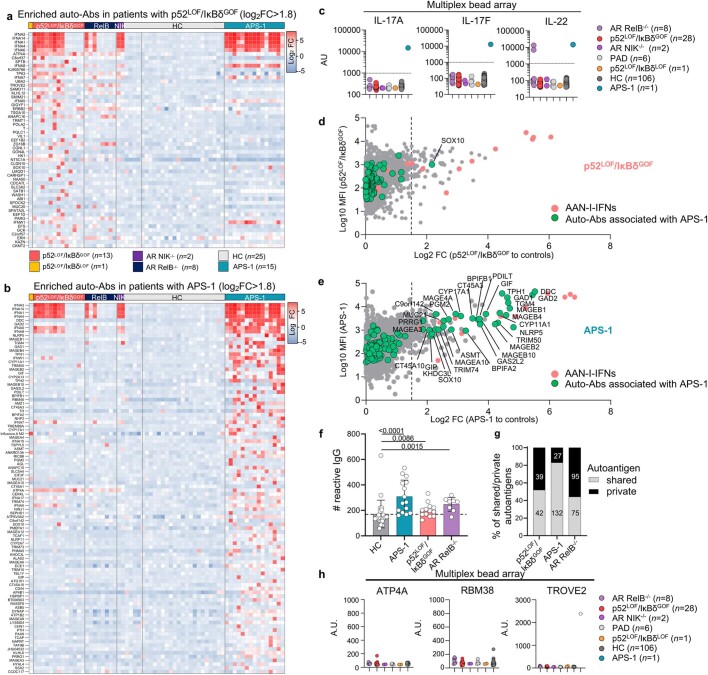
Narrow autoantibody profiles in patients with p52^LOF^/IκBδ^GOF^ variants. (**a-b**) Heat map of the autoantigens with the highest levels of enrichment in patients with a p52^LOF^/IκBδ^GOF^ variant (*n* = 13, **a**) or APS-1 patients (*n* = 15, **b**), versus patients with AR RelB deficiency (*n* = 8), AR NIK (*n* = 2) deficiency, or with APS-1 (*n* = 15), as determined with protein microarray (HuProt). Results are shown as the mean fluorescence of two technical replicates with a log_2_ fold-change >1.8 in patients with p52^LOF^/IκBδ^GOF^ variants **(a)** or APS-1 **(b)** relative to 25 healthy controls (HC). (**c**) Detection of IgG auto-Abs against IL-17A, IL-17F, or IL-22 using a multiplex bead array in patients with inborn errors of the alternative NF-κB pathway. Data representative of one independent experiment are shown. (**d**) Protein microarray distribution of auto-Abs against IFN-α and IFN-ω (red dots) or other autoantigens frequently found targeted in patients with APS-1 (green dots), in patients with a p52^LOF^/IκBδ^GOF^ variant, relative to controls. (**e**) Protein microarray distribution of auto-Abs against IFN-α and IFN-ω (red dots), or other autoantigens associated with APS-1 (green dots) in APS-1 patients relative to controls. (**f**) Number of autoreactive IgG in each patient (APS-1, p52^LOF^/IκBδ^GOF^, RelB^−/−^) or control, as determined by the sum of autoantigens with a log_2_ FC > 1.5 relative to the mean value for all healthy controls (HC). The error bars represent the median ± s.d. of the autoreactive IgG in each group. Comparisons done using two-tailed Mann–Whitney test. (**g**) Proportion of shared (by ≥ 2 patients) and private reactive autoantigens in the group of patients with a p52^LOF^/IκBδ^GOF^ variant, APS-1, or AR RelB deficiency. (**h**) Detection of auto-Abs against ATP4A, RBM38, or TROVE2 in a multiplex bead array. The white dot indicates the positive control for the detection of anti-TROVE2 auto-Abs. A.U. corresponds to arbitrary units. Data representative of one independent experiment are shown.

The presence of autoantibodies against other proteins was assessed in patients with inborn errors of the alternative NF-κB using a panel of around 20,000 human proteins corresponding to a large proportion of the full-length proteome, many of which were in their native conformation (HuProt). Moreover, 15 patients with APS-1 and 25 healthy controls, all sex- and aged-matched with the 13 patients with p52^LOF^/IκBδ^GOF^ variants tested, were included. The IFNα subtypes and IFNω were among the autoantigens with the highest level of enrichment in the 13 patients with p52^LOF^/IκBδ^GOF^ tested relative to control plasma (log_2_-transformed fold change of >1.5) (Fig. 3i and Extended Data Fig. 8a). This enrichment was specific to the IFNα subtypes and IFNω, but not other type I IFNs (IFNβ, IFNκ or IFNε) or type III IFNs (Fig. 3i). By contrast, autoantibodies against IL-17A, IL-17F and IL-22 (multiplex beads assay) and most of the other autoantigens commonly identified in cohorts of patients with APS-1 (HuProt microarray) were not found in patients with p52^LOF^/IκBδ^GOF^ variants (Extended Data Fig. 8b–e). Patients with p52^LOF^/IκBδ^GOF^ variants had a lower diversity of IgG-binding autoantigens compared with patients with APS-1 (*n* = 81 and 159 targeted proteins, respectively). Moreover, half (*n* = 39, 48%) of the enriched reactive autoantigens in patients with p52^LOF^/IκBδ^GOF^ variants were private, whereas a much smaller proportion (*n* = 27, 17%) of those enriched in patients patients with APS-1 was private (Extended Data Fig. 8f,g). There were only 12 overlapping IgG-reactive autoantigens, 10 of which were IFNω or IFNα subtypes (Fig. 3j). Most of the reactivities other than those to type I IFNs identified in patients with p52^LOF^/IκBδ^GOF^ variants by HuProt were not detected in a multiplex bead assay (Extended Data Fig. 8h), whereas no pituitary, skin or other tissue-specific autoantigens were detected by HuProt in these patients. We confirmed, by classical diagnostic methods, that almost all of the patients (26 out of 30, 87%) with p52^LOF^/IκBδ^GOF^ variants lacked the tissue-specific autoantibodies typically observed in patients with APS-1 (detected in 25 out of 31, 81%) (Supplementary Fig. 12). These data suggest that autoantibodies neutralizing the 12 IFNα subtypes and IFNω are the principal disease-associated autoantibodies detected in patients with inborn errors of the alternative NF-κB pathway.

## AAN-I-IFNs underlie viral susceptibility

**Fig. 4:**
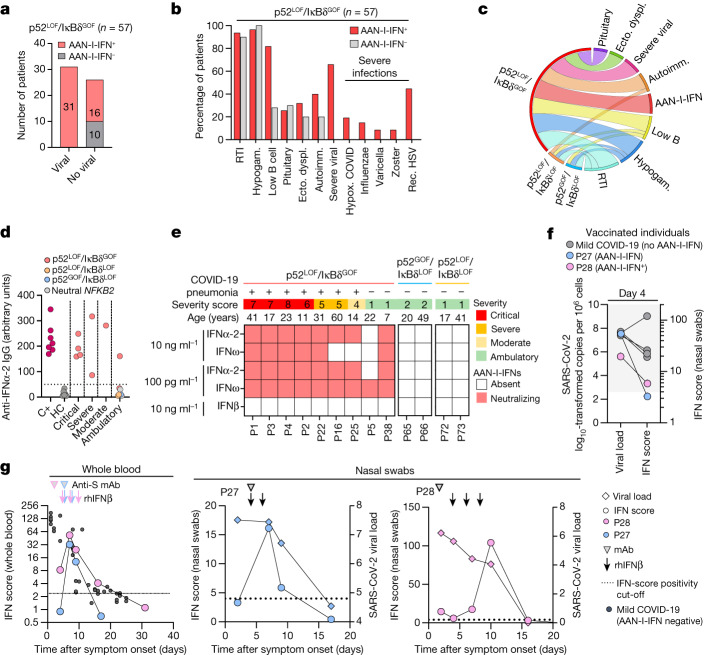
Susceptibility to COVID-19 pneumonia and other severe viral diseases is strongly associated with the presence of AAN-I-IFNs. **a**, The number of patients with a p52^LOF^/IκBδ^GOF^ variant and manifestations of viral diseases as a function of their AAN-I-IFN status. **b**, Clinical and immunological manifestations in patients with a p52^LOF^/IκBδ^GOF^ variant, as a function of their AAN-I-IFN status. Autoimm., autoimmunity; ecto. dyspl., ectodermal dysplasia; hypogam., hypogammaglobulinaemia; hypox., hypoxaemic; rec., recurrent; RTI, recurrent bacterial respiratory tract infection. **c**, Chord diagram of the main clinical and immunological manifestations of patients with inborn errors of NF-κB2. **d**, Anti-IFNα-2 IgG detection by Gyros in positive control individuals (*n* = 10), healthy control individuals (*n* = 7), patients with a p52^LOF^/IκBδ^GOF^ (*n* = 9), p52^LOF^/IκBδ^LOF^ (*n* = 2), p52^GOF^/IκBδ^LOF^ (*n* = 2) or neutral (*n* = 2) NF-κB2 variant and COVID-19, as a function of disease severity. **e**, Heat map showing the type I IFN neutralization profile of unvaccinated patients during COVID-19, according to disease severity and clinical presentation during infection, including patients with a p52^LOF^/IκBδ^GOF^ (*n* = 9), p52^GOF^/IκBδ^LOF^ (*n* = 2) or p52^LOF^/IκBδ^LOF^ (*n* = 2) variant. The red squares indicate a complete neutralization ability of the plasma for ISRE induction in the luciferase reporter assay system, and the white squares indicate a total absence of neutralizing autoantibody detection in the ISRE–luciferase assay. **f**, The viral load and IFN score in nasal swabs over the course of SARS-CoV-2 infection in patients with a p52^LOF^/IκBδ^GOF^ variant (*n* = 2) with AAN-I-IFNs, and in vaccinated individuals with a mild disease and no AAN-I-IFNs (*n* = 4). **g**, The IFN score and viral load in whole blood (left) or nasal swabs (right) over the course of SARS-CoV2 infection in patients with a p52^LOF^/IκBδ^GOF^ variant with AAN-I-IFNs (*n* = 2), or in individuals infected with SARS-CoV-2 presenting only mild disease (*n* = 36). The vertical arrows indicate the times of recombinant human IFNβ (rhIFNβ) injection and the arrowheads indicate the infusion of monoclonal antibodies (mAbs) against SARS-CoV-2 spike protein.

**Extended Data Fig. 9:**
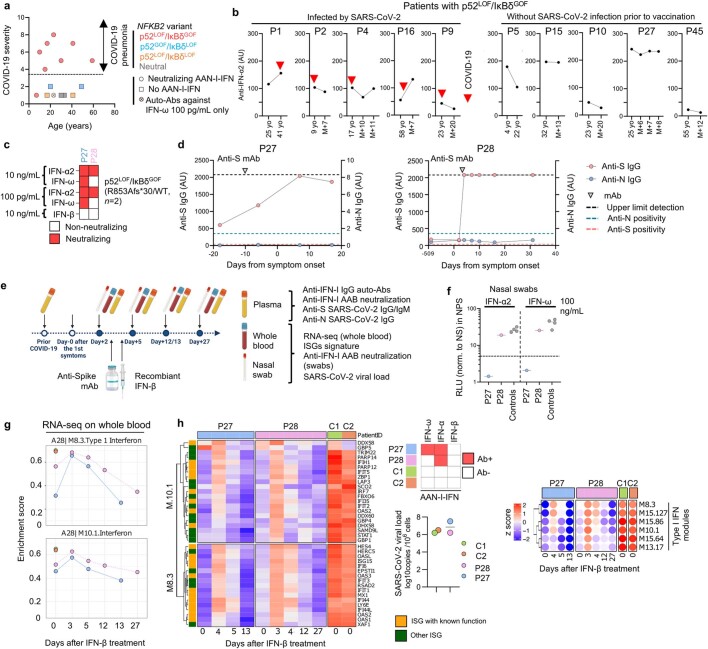
AAN-I-IFNs prevent ISG induction in blood and the upper respiratory tract during COVID-19, a defect that can be rescued by exogenous IFN-β treatment. (**a**) Correlation between age and COVID-19 severity in patients with inborn errors of NF-κB2. The crossed light red square represents a patient with auto-Abs neutralizing only IFN-ω at 100 pg/mL. **(b)** Changes in the titres of auto-Abs against IFN-α2, as measured by Gyros, with age, in patients with a p52^LOF^/IκBδ^GOF^ variant and COVID-19. Red arrows indicate the onset of COVID-19. (**c**) Heatmap showing the neutralization profile of P27 et P28 heterozygous for a p52^LOF^/IκBδ^GOF^ variant (R853Afs*30/WT) during COVID-19. (**d**) Longitudinal follow-up of anti-S and anti-N IgG in P27 and P28 during the course of COVID-19, before and after treatment by the infusion of an anti-S monoclonal Ab (mAb, grey arrow). **(e)** Overview of the longitudinal investigation of COVID-19 episodes in P27 et P28. (**f**) Neutralization capacity of the nasal swab from P27 et P28 upon SARS-CoV-2 infection and individuals infected with the omicron variant but without detectable AAN-I-IFNs (controls, *n* = 4, grey dots). Bars represent the median. **(g)** Longitudinal IFN module enrichment score during the course of COVID-19 in P27 and P28 and in two age-matched controls infected with SARS-CoV-2. IFN modules M.10.1 and M.8.3 are represented. Values obtained before and after the treatment of P27 and P28 with IFN-β. (**h**) ISG score induction by IFN module analysis during the course of COVID-19 in P27 and P28, before and after recombinant IFN-β treatment, and in age-matched controls (C1 and C2, *n* = 2) infected with SARS-CoV-2.

We hypothesized that the susceptibility to viral diseases, including COVID-19, reported in patients with inborn errors of the alternative NF-κB pathway might be at least partly explained by the presence of AAN-I-IFNs. All of the patients (*n* = 31) with p52^LOF^/IκBδ^GOF^ variants and severe viral infections had AAN-I-IFNs, including all of those with severe forms of COVID-19, influenza, varicella zoster virus or recurrent HSV-1 disease (Fig. 4a). Furthermore, at least one episode of severe or recurrent viral disease was reported in 31 of the 47 (66%) patients with p52^LOF^/IκBδ^GOF^ variants and AAN-I-IFNs, but not in those without such antibodies. With the exception of viral susceptibility and B cell lymphopenia, there were no strong clinical or immunological differences between patients with p52^LOF^/IκBδ^GOF^ variants with and without AAN-I-IFNs (Fig. 4b,c). Two out of the eight patients with autosomal-recessive RELB deficiency developed a severe viral disease (varicella pneumonia, *n* = 2; and PML, *n* = 1), and both had autoantibodies neutralizing IFNα and IFNω (Supplementary Table 2). All seven patients with p52^LOF^/IκBδ^GOF^ variants who developed COVID-19 pneumonia during the prevaccination period had neutralizing autoantibodies against both IFNα-2 and IFNω, and experienced critical (*n* = 4), severe (*n* = 2) or moderate (*n* = 1) COVID-19 pneumonia (Fig. 4d,e, Extended Data Fig. 9a and Supplementary Table 6). Plasma samples collected from two of these patients (P1 and P16) before SARS-CoV-2 infection neutralized IFNα-2 and IFNω at a concentration of 10 ng ml^−1^. These samples were collected up to 16 years before COVID-19, demonstrating that these neutralizing autoantibodies were present before infection and were therefore not triggered by SARS-CoV-2 infection (Extended Data Fig. 9b). These autoantibodies against IFNα and IFNω blocked type I IFN signalling by impairing type I IFN ISG induction in vivo in the blood and upper respiratory tract during COVID-19, which could be rescued by exogenous IFNβ treatment in these patients (Supplementary Results 4, Extended Data Fig. 9c–h and Supplementary Fig. 13). Two other patients were infected without developing pneumonia or requiring hospitalization: one 22-year-old patient with autoantibodies neutralizing only IFNω at the lowest dose of 100 pg ml^−1^ (P5, S762Afs*21/wild type (WT)) and one 7-year-old patient with autoantibodies neutralizing both IFNα-2 and IFNω at a concentration of 10 ng ml^−1^ (P38, G869Vfs*18/WT) (Fig. 4d,e). The six infected patients without AAN-I-IFNs received ambulatory care and did not develop pneumonia. They were heterozygous for neutral (A567 and V661M) variants, for the Q539* p52^GOF^/IκBδ^LOF^ variant (*n* = 2) or for the R52*/WT p52^LOF^/IκBδ^LOF^ variant (*n* = 2) (Fig. 4d,e and Extended Data Fig. 9a). Furthermore, ten patients with a p52^LOF^/IκBδ^GOF^ variant and pre-existing AAN-I-IFNs encountered SARS-CoV-2 after vaccination (corresponding to the Omicron period, from October 2021 to February 2022) (Supplementary Fig. 14). They received an infusion of anti-SARS-CoV-2 monoclonal antibodies (*n* = 4, as sotrovimab (*n* = 3) or tixagevimab/cilgavimab (*n* = 1)), remdesivir (*n* = 1) or nirmatrelvir/ritonavir (*n* = 1) and/or recombinant IFNβ (*n* = 2) in addition to intravenous immunoglobulin supplementation (*n* = 10). All of these patients reported asymptomatic to mild (NIH scale, 1–3) COVID-19 without pneumonia (Supplementary Fig. 14 and Supplementary Table 6). P3, who developed critical COVID-19 pneumonia during the first wave of the SARS-CoV-2 pandemic, developed ambulatory disease (NIH score, 2) after vaccination and the therapeutic infusion of sotrovimab. The two patients with p52^LOF^/IκBδ^LOF^ variants (P43 and P63) and three with p52^GOF^/IκBδ^LOF^ variants (P39, P40 and P41) without AAN-I-IFNs had ambulatory disease. Overall, these results indicate that AAN-I-IFNs are clinically important, underlying severe forms of COVID-19 pneumonia and, probably, other severe viral diseases, including influenza pneumonia and severe varicella.

## AIRE expression in alternative NF-κB IEIs

**Fig. 5:**
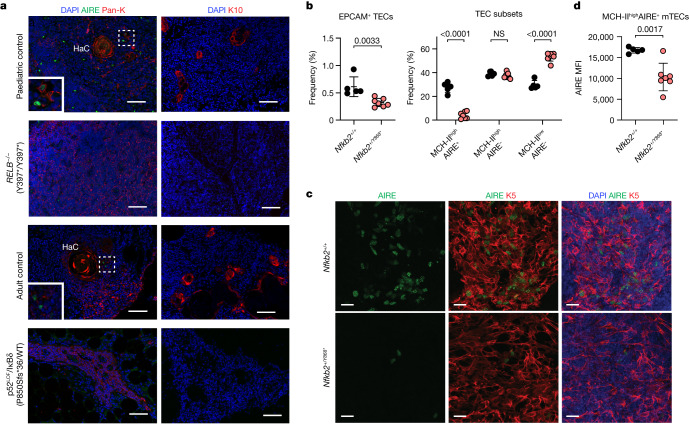
Impaired mTEC development and thymic AIRE expression in a patient with autosomal-recessive RELB deficiency, a patient heterozygous for a p52^LOF^/IκBδ^GOF^ NF-κB2 variant and in mice heterozygous for the Y868* NF-κB2 variant. **a**, Immunofluorescence staining of thymic tissue from age-matched controls, a patient with autosomal-recessive complete RELB deficiency or heterozygous for a p52^LOF^/IκBδ^GOF^ NF-κB2 variant. AIRE-expressing cells (green) and Hassall’s corpuscles (HaC) are shown on the left. Pan-K, pan-keratin. Staining for K10 (red), defining terminally differentiated corneocyte-like mTECs, is shown on the right. DAPI staining is shown in blue. Scale bars, 50 μm (left) and 100 μm (right). Inset: the controls at a higher magnification. Data shown are representative of one independent experiment. **b**, The percentage of EPCAM^+^CD45^−^ thymic epithelial cells (TECs), and the various TEC subsets (defined on the basis of their MHC class II (MHC-II) and AIRE expression) in WT controls (*Nfkb2*^*+/+*^, black dots, *n* = 5) and mice carrying a heterozygous missense variant homologous to the human Y868* p52^LOF^/IκBδ^GOF^ NF-κB2 variant (*Nfkb2*^+/Y868*^, red dots, *n* = 7). Statistical comparisons were performed using unpaired, parametric, two-tailed Student’s *t*-tests (EPCAM^+^ TECs) or two-way nonparametric analysis of variance (ANOVA) (Sidak’s test) with correction for multiple comparisons (TEC subsets). Data are mean ± s.d. Data shown are representative of three independent experiments. **c**, Representative confocal microscopy images of AIRE (green), K5 (red) and DAPI (blue) of WT (*Nfkb2*^*+/+*^, *n* = 3, top) and *Nfkb2*^+/Y868*^ (*n* = 3, bottom) mouse thymuses. Scale bars, 20 μm. Data shown are representative of two independent experiments. **d**, Mean fluorescence intensity (MFI) of AIRE expression in mature MHC-II^high^AIRE^+^ mTECs from WT (*n* = 5) and *Nfkb2*^+/Y868*^ (*n* = 7) mouse thymuses. Statistical comparisons were performed using unpaired, parametric two-tailed Student’s *t*-tests. Data are mean ± s.d. Data shown are representative of three independent experiments. Source Data

**Extended Data Fig. 10:**
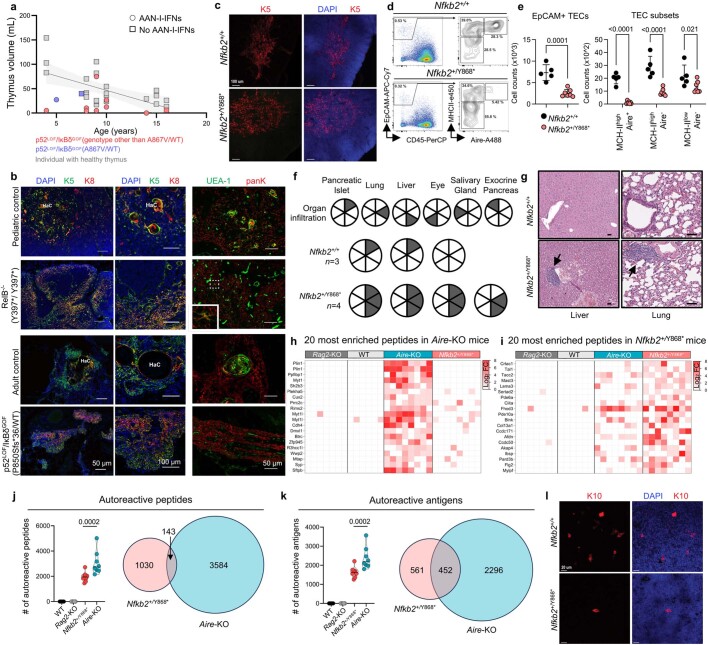
Thymus volume in p52^LOF^/IκBδ^GOF^ children, and autoimmune pathology and autoreactive IgG profiles in *Nfkb2*^+/Y868*^ mice. (**a**) Estimation of thymus volume in p52^LOF^/IκBδ^GOF^ patients relative to aged-matched controls aged from 3 to 16 years. Black line represents simple linear regression of the control thymus volume with its 95% confidence bands (grey area). Data representative of one independent experiment are shown. (**b**) Immunofluorescence staining of thymic tissue from age-matched controls and patients with AR complete RelB deficiency or a p52^LOF^/IκBδ^GOF^ variant. HaC, Hassall’s corpuscles. Scale bars, 50 μm (left and right panel) or 100 μm (central panel). (**c**) Representative confocal microscopy images of the thymic medulla stained for cytokeratin 5 (K5) for the WT controls (*Nfkb2*^*+/+*^, *n* = 3) or *Nfkb2*^*+/*Y868*^ mice (*n* = 3). Scale bar, 100 μm. Data representative of two independent experiment are shown. (**d**) Contour plot for Aire expression in mature MHC-II^+^ mTECs. (**e**) Absolute number of EpCAM^+^CD45^-^ thymic epithelial cells (TECs), and TEC subsets in *Nfkb2*^*+/+*^ (*n* = 5) and *Nfkb2*^*+/*Y868*^ (*n* = 7) mice. Comparisons done using unpaired, parametric, two-tailed Student’s *t*-test (for EpCAM^+^ TECs) or two-way non-parametric ANOVA (Sidak’s test) with correction for multiple comparisons (for TEC subsets). Three independent experiments were performed. (**f**) Summary of lymphocytic cell infiltrates in *Nfkb2*^*+/+*^ (*n* = 3) and *Nfkb2*^*+/*Y868*^ (*n* = 4) mice. Each circle represents an individual animal, and each slice of the circle represents a tested organ. Lymphocytic infiltrates of the designated organ are indicated by the grey-shaded sections of the circle. One independent experiment was performed. (**g**) Representative tissue sections stained with hematoxylin and eosin (H&E) showing lymphocytic infiltrates (black arrows) in *Nfkb2*^*+/+*^ or *Nfkb2*^*+/*Y868*^ mice. Scale bar, 50 μm. Data representative of one independent experiment are shown. (**h-i**) Heatmap of the top 20 autoreactivities by degree of enrichment, in *Aire*-KO (**h**) or *Nfkb2*^+/Y868*^ (**i**) mice relative to WT and *Rag2*-KO mice. (**j-k**) Number of autoreactive peptides (**j**) or antigens (**k**) displaying enrichment in WT, *Rag2*-KO, *Nfkb2*^+/Y868*^ and *Aire*-KO mice, and representation of the autoreactive peptide and antigen profiles of *Nfkb2*^+/Y868*^ and *Aire*-KO mice, with their overlap. Comparisons done using two-tailed Mann–Whitney test. (**l**) Representative confocal microscopy images of K10 and DAPI staining on *Nfkb2*^+/+^ (*n* = 3) and *Nfkb2*^*+/*Y868*^ (*n* = 3) mouse thymuses. Scale bars, 20 μm. Data representative of two independent experiments are shown. Source Data

In mice, mTEC development and AIRE expression are dependent on the alternative NF-κB pathway, through NIK and RELB^24–26^. Consequently, *Relb*- and *Nik*-deficient mice, and mice heterozygous for a p52^LOF^/IκBδ^GOF^ variant, display thymic hypoplasia with weak medullary thymic formation, impaired maturation of AIRE-expressing mTECs and tolerance breakdown^26,30,31^. In human fetal thymuses, *NFKB2* and *RELB* transcripts are highly abundant in AIRE^+^ mTECs^32^. However, the impact of deleterious variants affecting the alternative NK-κB pathway on human AIRE expression remains unclear. We hypothesized that patients with inborn errors of NIK, RELB or NF-κB2 develop AAN-I-IFNs due to insufficient AIRE expression in the thymus. An analysis of the thymic volume in patients with p52^LOF^/IκBδ^GOF^ variants (*n* = 11) aged 4 to 16 years revealed that the total thymic volume was smaller in these patients compared with age-matched controls with conditions unrelated to immunity (Extended Data Fig. 10a). We next analysed thymic biopsy samples from a patient with autosomal-recessive complete RELB deficiency (mutation Y397*/Y397*, biopsy performed at the age of one year, with AAN-I-IFNs) and a deceased patient with a p52^LOF^/IκBδ^GOF^ variant (P850Sfs*36/WT, the sample was collected at the age of 27 years; no plasma sample was available). Immunofluorescence analysis of the thymic tissue sections from these two patients revealed a dysplastic organ with a disorganized corticomedullary architecture and atrophic medulla (Extended Data Fig. 10b). A residual epithelial cell population (pan-keratin-expressing cells) with disorganized keratin 5 (K5)- and keratin 8 (K8)-positive cells was detected in the thymuses of both patients (Extended Data Fig. 10b). mTECs (defined as pan-keratin^+^UEA-1^+^ cells) were rare, but not entirely absent, in the thymus of the patient with autosomal-recessive RELB deficiency (Extended Data Fig. 10b). However, no AIRE or keratin 10 (K10)-positive Hassall’s corpuscles were detected (Fig. 5a). These findings suggest that RELB deficiency does not completely block mTEC specification but, rather, prevents differentiation into AIRE-expressing and post-AIRE mTECs. An analysis of the thymus from the adult patient with a p52^LOF^/IκBδ^GOF^ variant showed that, relative to an age-matched control thymus, the thymus tissue from this patient lacked UEA-1^+^ mTECs, AIRE and Hassall’s corpuscles (Fig. 5a and Extended Data Fig. 10b). These data suggest that the p52^LOF^/IκBδ^GOF^ genotype impaired the maturation of human AIRE-expressing cells. However, thymic involution in this older patient made it difficult to draw definitive conclusions regarding the impact of the mutation on mTEC development earlier in life. Collectively, these data suggest that human p52–RELB heterodimers control the development of mature mTECs and the thymic expression of AIRE, and that inborn errors of the human alternative NF-κB pathway underlie the production of AAN-I-IFNs due to the impaired development of mature AIRE-expressing mTECs.

## Aire expression in *Nfkb2*^+/Y868*^ mice

We further investigated the role of the p52–RELB heterodimer in mature AIRE^+^ mTEC development by generating mice carrying a heterozygous variant homologous to the human Y868* p52^LOF^/IκBδ^GOF^ NF-κB2 variant (*Nfkb2*^+/Y868*^)^31,33^. Despite the relatively normal thymic medullary compartmentalization, as shown by K5 immunofluorescence staining (Extended Data Fig. 10c), the cellularity of the thymic epithelium was significantly lower in *Nfkb2*^+/Y868*^ mice compared with in WT mice, reflecting a dysregulation of mTEC development and homeostasis (Fig. 5b and Extended Data Fig. 10d,e). The proportion of AIRE^+^ mTECs and absolute counts for this subset were very low, but non-zero, in *Nfkb2*^+/Y868*^ mice relative to in WT mice, as shown by both flow cytometry and immunofluorescence staining of tissue sections (Fig. 5b,c and Extended Data Fig. 10d,e). The residual AIRE^+^ mTECs in the *Nfkb2*^+/Y868*^ mice displayed significantly weaker AIRE expression compared with their WT counterparts (Fig. 5d). Impaired mTEC development in *Nfkb2*^+/Y868*^ and *Nfkb2*^*+/D865G*^ mice causes T cell autoimmunity in multiple organs^31^. The lymphocytic infiltrates in the *Nfkb2*^+/Y868*^ mice affected the pancreatic islets, lung and liver, as in NOD *Aire*-KO mice of the same age^34^. However, in marked contrast to NOD *Aire*-KO mice, the salivary glands, exocrine pancreas and retina were spared (Extended Data Fig. 10f,g). Consistently, the autoreactive IgG profiles of *Nfkb2*^+/Y868*^ mice, like those of their human counterparts, are narrower than that of *Aire*-KO mice, with only a minimal overlap, as shown by analysis using phage-display immunoprecipitation and sequencing (PhIP–seq) (Extended Data Fig. 10j,k). After their maturation into MHC-II^high^AIRE^+^ mTECs, these cells can display a downregulation of AIRE expression and give rise to terminally differentiated mTECs (also called post-AIRE mTECs or mimetic cells), with distinctive extrathymic parenchyma-specific features^35,36^. In mice, AIRE expression and function are required for the development of some terminally differentiated mTECs, as shown by the small proportions of corneocyte-like mTECs in *Aire*-KO mice^35,37,38^. We therefore investigated the consequences of impaired p52–RELB heterodimer activation in mouse mTECs on the development of corneocyte-like mTECs by assessing K10 expression. Like their human counterparts, *Nfkb2*^+/Y868*^ mice had very small numbers of K10-expressing post-AIRE mTECs in the medulla (Extended Data Fig. 10i). These data strongly suggest that the human alternative NF-κB pathway is essential for the development of mature mTECs, as its defects prevent proper AIRE expression and the generation of other AIRE-dependent terminally differentiated mTECs, whereas this pathway appears to be redundant for the development of some other mTEC subsets (MHC^low^AIRE^−^ cells).

## Discussion

We found that human inborn errors of the alternative NF-κB pathway (autosomal-recessive NIK, autosomal-recessive RELB or autosomal-dominant p52^LOF^/IκBδ^GOF^ NF-κB2 disorders) define a new group of IEIs underlying the development of AAN-I-IFNs. The presence of these autoantibodies is consistent with the cellular phenotype found in the patients’ fibroblasts, culminating in defective p52–RELB activity, which may be secondary to the impaired processing of p100 to generate p52 or to a quantitative or qualitative RELB deficiency. By contrast, no AAN-I-IFNs were found in patients who were heterozygous for p100–IκBδ LOF variants, or in patients with inborn errors of the canonical NF-κB pathway. This suggests that a correct NIK-dependent processing of p100 is a key checkpoint for the p52–RELB-dependent activation of the alternative NF-κB pathway that is required to prevent the development of AAN-I-IFNs.

The p52–RELB heterodimers control AIRE expression in mouse mTECs^29^. Indeed, *Map3k14*- (encoding NIK), *Ikka*- or *Relb*-deficient mice, and *Nfkb2*^+/Y868*^ mice have strongly reduced thymic AIRE expression^26,31,39,40^, and the deletion of the enhancer element containing two NF‐κB binding sites upstream from the *Aire*-coding locus phenocopies *Aire* deficiency^41^. We detected no AIRE expression in human thymuses lacking RELB or heterozygous for a p52^LOF^/IκBδ^GOF^ variant. Moreover, the development of terminally differentiated corneocyte-like mTECs was impaired in both humans and mice with defective p52–RELB activity. This finding is consistent with the small size of this population in *Aire*-KO mice, and the role of AIRE in decreasing chromatin accessibility at NF-κB regulatory elements in mature mTECs, facilitating their terminal differentiation^35,37,38,42^. Thus, the alternative NF-κB pathway appears to be essential for the development of mature mTECs in humans, and for proper thymic AIRE expression, by ensuring correct p52–RELB activation.

The notable association between the presence of AAN-I-IFNs in patients with human inborn errors of NIK, RELB and NF-κB2 suggests that intact p52–RELB activation is essential to prevent the breakdown of AIRE-dependent central T cell tolerance toward type I IFNs in humans. Causality is supported by several lines of evidence: the development of these AAN-I-IFNs in almost all, if not all, humans with inherited AIRE deficiency, regardless of age or ancestry^1,2,23,43–48^; the reduced AIRE expression in patients with other germline (hypomorphic *RAG1* or *RAG2* variants^20–22^) or somatic (mTEC neoplasia^49^) conditions underlying the development of these autoantibodies; the impaired development of AIRE-expressing mTECs in both mouse and human disorders of the alternative NF-κB pathway^26,31,39,40^; the absence of Hassall’s corpuscles in patients with inborn errors of the alternative NF-κB pathway, which mirrors the decreased levels of terminally differentiated corneocytes observed in *Nfkb2*^+/Y868*^ mice and *Aire*-KO mice; and the persistence of these AAN-I-IFNs up to 14 years after HSCT with engraftment.

However, it is surprising that AIRE deficiency in patients with inborn errors of the alternative NF-κB pathway leads to such an apparently narrow breakdown of central tolerance, restricted almost exclusively to the 12 IFNα subtypes and IFNω, when pathogenic autoantibodies are considered. This situation contrasts with the immunological and clinical manifestations of patients with APS-1, which only partially overlap those of patients with inborn errors of the NF-κB pathway^44,45^. The absence of the typical clinical and immunological features of APS-1 other than AAN-I-IFNs in patients with p52^LOF^/IκBδ^GOF^ variants may be attributed to the presence of residual mature mTECs or terminally differentiated mTECs (mimetic cells) that would ensure central tolerance to the other antigens targeted in APS-1^35,36,50^. Conversely, the low blood counts of B and T_FH_ cells in patients with p52^LOF^/IκBδ^GOF^ variants, but not in patients with APS-1, may be a result of the impaired alternative NF-κB pathway in B cells or in non-mTEC stromal cells^51^. The low T_reg_ cell counts of patients with p52^LOF^/IκBδ^GOF^ variants may result from an impaired alternative NF-κB pathway in T cells or impaired AIRE expression in mTECs^31,52,53^. The clinical manifestations in patients with p52^LOF^/IκBδ^GOF^ variants also differ from those in patients with the other two forms of autosomal-dominant inborn errors of NF-κB2, probably due to the higher levels of IκBδ activity of the mutant protein (Supplementary Table 7).

Our findings confirm the detrimental consequences of the presence of AAN-I-IFNs for viral susceptibility (COVID-19 pneumonia, influenza pneumonia and herpesvirus diseases)^3,7,13,23^. Despite their high risk of developing life-threatening COVID-19 pneumonia, unvaccinated patients with inborn errors of the alternative NF-κB pathway displayed a high but incomplete penetrance of hypoxaemic COVID-19 pneumonias, as reported in patients with APS-1 or SLE^3,54,55^. Additional protective or risk factors may be required in these patients to influence the clinical outcome of COVID-19, such as age or the nature of the AAN-I-IFNs (neutralizing IFNω and/or the 12 IFNα subtypes). Our findings also suggest that a reinforcement of prophylactic or therapeutic interventions can improve the clinical outcome of viral diseases in patients with AAN-I-IFNs, throughout their lives, as these autoantibodies may persist even after HSCT^12^. Collectively, these results suggest that the human alternative NF-κB pathway controls AIRE expression in mTECs and that human inborn errors of this pathway thereby underlie the development of AAN-I-IFNs and the resulting predisposition to viral infection. They confirm that at least some individuals develop AAN-I-IFNs because of an underlying IEI, suggesting that other genetic aetiologies remain to be discovered in the 0.3% to 2% of individuals under 70 years of age who carry such autoantibodies. The observation that genetic aetiologies of *AIRE* in *cis* or in *trans* that disrupt central T cell tolerance underlie these autoantibodies suggests that as yet undiscovered genetic aetiologies may also affect this process. The genetic study of patients with AAN-I-IFNs may reveal new molecular components in this or other processes. What triggers the rise in autoantibody levels against type I IFNs after the age of 70 years is another related question potentially linked to thymic involution.

## Methods

### Participants and samples

We enrolled 73 patients with rare variants of *NFKB2* though an international collaborative study. Data were collected through an anonymized survey sent to specialists in immunology or paediatrics with reported or unreported patients with these IEIs (Supplementary Table 1). All index cases were genotyped after suspicion of an inborn error of immunity. An analysis of the familial segregation of each *NFKB2* variant was performed in all relatives for whom genomic DNA was available. We included all individuals heterozygous for a rare (MAF < 0.0001) *NFKB2* non-synonymous variant (detected by Sanger sequencing: *n* = 19; IEI gene NGS panel: *n* = 26; whole-exome sequencing (WES): *n* = 23; or whole-genome sequencing (WGS): *n* = 5) for whom a plasma/serum sample was also available. Clinical and immunological data were collected with a standardized questionnaire using Microsoft Excel, together with at least one plasma sample. The plasma samples from patient P1 were obtained through the NCT03394053 and NCT03610802 protocols with the approval of the National Institutes of Health institutional review board. We also enrolled 14 patients with other inborn errors of the alternative NF-κB pathway including patients with autosomal-recessive complete NIK deficiency (*n* = 2 from 2 kindreds^57^ and unpublished results); autosomal-recessive partial (*n* = 4 from 2 kindreds) or complete (*n* = 4 from 2 kindreds) RELB deficiency^57–61^ (and unpublished results), or the related TNF receptors (TNFR) (autosomal-recessive complete BAFFR deficiency (*n* = 1)^58^); or X-linked recessive CD40L deficiency (*n* = 3 from 3 kindreds (unpublished)). They were detected by Sanger sequencing: *n* = 7; and WES: *n* = 7. No plasma from patients with autosomal-recessive IKKα deficiency was available.

### Definitions and outcome measures

PAD was defined by the association of hypogammaglobulinaemia and recurrent bacterial respiratory tract infections^62^. Ectodermal dysplasia was defined by the association of sparse hair, eyebrows, or eyelashes, or nail dysplasia, with or without alopecia areata or totalis.

The severity of COVID-19 was defined according to the NIH ordinal scale, as previously reported^8,63^. The NIH scale is an eight-point ordinal scale ranging from ambulatory (1, no limitations of activities; 2, limitation in activity), to hospitalized (3, not requiring supplemental oxygen), moderate (4, not requiring supplemental oxygen but requiring ongoing medical care (related to COVID-19 or to other medical conditions)), severe (5, requiring supplemental oxygen) or critical (6, requiring non-invasive ventilation or use of high-flow oxygen devices; 7, receiving invasive mechanical ventilation or extracorporeal membrane oxygenation (ECMO); and 8, death).

### Plasmids and mutagenesis

The *NFKB2* (encoding p100), *RELB* and *MAP3K14* (encoding NIK) plasmids were obtained from Origen with a C-terminal DDK tag. The κB reporter construct (κB-luc), pGL4.32[luc2P/NF-κB-RE/Hygro] and pRL-SV40 vectors were obtained from a previous study^64^. Site-directed mutagenesis was performed as previously described^64^.

### Cell culture and transfection

HEK293T cells or HeLa cells (American Type Culture Collection) were maintained in Dulbecco’s modified Eagle medium (DMEM; Gibco) supplemented with 10% FBS (Gibco). Transient transfection was performed using X-tremeGENE 9 DNA Transfection Reagent (Merck) according to the manufacturer’s instructions. The cell lines were regularly tested and were found to be free of mycoplasma contamination.

### Functional evaluation of *NFKB2* variants

#### Luciferase reporter assays

The luciferase reporter assay was performed as previously described^64^. WT HEK293T cells in 96-well plates were transfected with a κB reporter plasmid (100 ng per well), the pRL-SV40 vector (10 ng per well), WT *MAP3K15*, WT *RELB*, and a WT or mutant p100 in the presence of X-tremeGENE 9 DNA Transfection Reagent (Merck). After incubation for 24 to 48 h, cells were collected, and luciferase activity was measured with the Dual-Glo Luciferase Assay System (Promega). We considered a deleterious variant to be p52-LOF if its luciferase activity was equivalent to that after cotransfection with EV, *RELB* and *MAP3K14*, hypomorphic if this activity was more than half that of the WT allele, and p52 gain-of-function (GOF) if this activity was less than half of that after cotransfection with *RELB*, *MAP3K14* and WT *NFKB2*.

#### Western blotting

Whole-cell lysates from HEK293T cells, MDDC, T cell blasts, primary or SV-40-transformed fibroblasts were prepared in RIPA buffer (50 mM Tris-HCl, pH 7.5, 150 mM NaCl, 1% Nonidet P40, 0.5% sodium deoxycholate and 0.1% SDS) supplemented with Complete Protease Inhibitor Cocktail (Roche). Proteins were separated by electrophoresis in 10% PROTEAN TGX Precast Protein Gels (Bio-Rad), and transferred onto Immobilon-P polyvinylidene fluoride membrane (Millipore). All blots were incubated overnight with primary antibodies and developed with the Pierce ECL Western Blotting Substrate (Thermo Fisher Scientific). The antibodies used in this study included antibodies against p100/p52 (4882; Cell Signaling Technology, 1:1,000), p105/p50 (N terminus; 3035; Cell Signaling Technology, 1:1,000), p65 (sc-372; Santa Cruz Biotechnology, 1:1,000), RELB (sc-48366; Santa Cruz Biotechnology, 1:800), REL (sc-6955; Santa Cruz Biotechnology, 1:1,000), and the following secondary antibodies: Amersham ECL mouse IgG, HRP-linked whole antibody (from sheep; NA931; GE Healthcare Life Sciences) and Amersham ECL rabbit IgG, HRP-linked whole antibody (from donkey; NA934; GE Healthcare Life Sciences). Uncropped western blots can be found in Supplementary Fig. 1.

#### Confocal microscopy

HeLa cells were plated on chambered coverslips (80826, iBidi) and were left untransfected or were transiently transfected with a plasmid encoding p100, RELB and/or NIK and/or an empty pCMV6 vector for 48 h. Primary or SV-40 fibroblasts were plated on chamber coverslips and left unstimulated or were stimulated with 100 ng ml^−1^ Lt or 100 ng ml^−1^ TWEAK for 48 h. The cells were fixed in 4% formaldehyde in phosphate-buffered saline (PBS), pH 7.4. Cells were incubated overnight at 4 °C with anti-p100/p52 (4882; Cell Signaling Technology, 1:1,000), or RELB (sc-48366; Santa Cruz Biotechnology, 1:800) primary antibodies. The cells were washed three times with 1× PBS and stained by incubation with secondary antibodies for 1 h at room temperature (goat anti-mouse IgG Alexa Fluor 488 (A-11029, 1:250); goat anti-rabbit IgG Alexa Fluor 633 (A-11037, 1:250) before mounting in Prolong-gold and visualization by confocal microscopy (×63 or ×40 oil-immersion lens).

### Detection and functional evaluation of anti-cytokine autoantibodies

#### Gyros

Cytokines, rhIFNα-2 (Miltenyi Biotec, 130-108-984) or rhIFNω (Merck, SRP3061) were first biotinylated with EZ-Link Sulfo-NHS-LC-Biotin (Thermo Fisher Scientific, A39257), according to the manufacturer’s instructions, with a biotin-to-protein molar ratio of 1:12. The detection reagent contained a secondary antibody (Alexa Fluor 647 goat anti-human IgG (Thermo Fisher Scientific, A21445) diluted in Rexip F (Gyros Protein Technologies, P0004825); 1/500 dilution of the 2 mg ml^−1^ stock to yield a final concentration of 4 µg ml^−1^). PBS-T 0.01% buffer and Gyros Wash buffer (Gyros Protein Technologies, P0020087) were prepared according to the manufacturer’s instructions. Plasma or serum samples were then diluted 1/100 in PBS-T 0.01% and tested with Bioaffy 1000 CD (Gyros Protein Technologies, P0004253), and Gyrolab X-Pand (Gyros Protein Technologies, P0020520). Cleaning cycles were performed in 20% ethanol.

#### Luciferase reporter assays

The blocking activity of anti-IFNα-2 and anti-IFNω autoantibodies was determined with a reporter luciferase assay. In brief, HEK293T cells were transfected with a plasmid containing the firefly luciferase gene under the control of the human *ISRE* promoter in the pGL4.45 backbone, and a plasmid constitutively expressing *Renilla* luciferase for normalization (pRL-SV40). Cells were transfected in the presence of the X-tremeGene 9 transfection reagent (Sigma-Aldrich, 6365779001) for 24 h. Cells in DMEM (Thermo Fisher Scientific) supplemented with 2% fetal calf serum and 10% healthy donors or patient serum/plasma were either left unstimulated or were stimulated with IFNα-2 (Miltenyi Biotec, 130-108-984) or IFNω (Merck, SRP3061) at 10 ng ml^−1^ or 100 pg ml^−1^, or with IFNβ (Miltenyi Biotech, 130-107-888) at 10 ng ml^−1^ or 1 ng ml^−1^, or with one of the 13 IFNα subtypes for 16 h at 37 °C. Each sample was tested once for each cytokine and dose in at least two independent experiments. Finally, cells were lysed for 20 min at room temperature and the luciferase levels were measured using the Dual-Luciferase Reporter 1000 assay system (Promega, E1980), according to the manufacturer’s protocol. Luminescence intensity was measured with a VICTOR X Multilabel Plate Reader (PerkinElmer Life Sciences). RLA was calculated by normalizing firefly luciferase activity against *Renilla* luciferase activity, and then normalizing against non-stimulated conditions. The samples were considered to be neutralizing if the luciferase activity signal, normalized to the non-stimulated conditions, was below 5.

#### Protein microarray

Protein microarrays (HuProt, CDI laboratories) were incubated in 5 ml blocking buffer, consisting of 2% bovine serum albumin and 0.05% Tween-20 in PBS, for 90 min. The arrays were then incubated overnight in 5 ml blocking buffer per array with serum from a blood donor or patient diluted 1:2,000. Each array was then washed five times, for 5 min each, with 5 ml PBS-T (PBS + 0.05% Tween-20). Alexa Fluor 647 goat anti-human IgG (Thermo Fisher Scientific, A-21445, RRID:AB_2535862) and Dylight 550 goat anti-GST (Columbia Biosciences, D9-1310) were diluted in blocking buffer (1:2,000 and 1:10,000, respectively) and each array was incubated in 5 ml of the resulting mixture for 90 min. Five washes were then conducted as previously described. Incubations and washes were performed on an orbital shaker, with aluminium foil to block out the light during the steps after adding the fluorescent antibodies. Finally, each array was immersed in deionized water three times and centrifuged for approximately 30 s for drying. The arrays were scanned later the same day with an Innoscan 1100AL Fluorescence scanner (Innopsys) using Mapix v.9.1.0 and the resulting images were analysed with the Jan 18-22 Huprot v4.0 Genepix Array List file and either of GenePix Pro v.5.1.0.19 or GenePix Pro 7. Normalization was used to compensate for variation in the signal intensity between experiments. Data from additional healthy donors from separate protein array experiments was included. Signal intensities were extracted from the scanned image with GenePix Pro v.5.1.0.19 and GenePix Pro 7, with the subtraction of the local background. IgG-reactive proteins were identified as proteins with a fluorescence intensity log_2_[fold change] ≥ 1.5. Autoantigens identified in patients with APS-1 were extracted from previous studies^46,47,65^. Protein arrays were performed on plasma from 24 patients with inborn errors of the alternative NF-κB pathway, with (*n* = 15) or without (*n* = 9) AAN-I-IFNs: p52^LOF^/IκBδ^GOF^ variant (*n* = 8 and 5), autosomal-recessive RELB (*n* = 5 and 3) and autosomal-recessive NIK (*n* = 2 with AAN-I-IFNs) deficiency, and the p52^LOF^/IκBδ^LOF^ variant (*n* = 1, without AAN-I-IFNs). Moreover, plasma from patients with APS-1 (*n* = 15) and healthy donors (*n* = 25) was included, sex- and aged-matched with the 13 patients with the p52^LOF^/IκBδ^GOF^ variant.

#### Multiplex bead arrays

The method for detecting human IgG in the serum using magnetic beads was described previously^66^. We used this method with a few modifications, as specified below. The AnteoTech Activation Kit for Multiplex Microspheres (A-LMPAKMM-10) was used in accordance with the manufacturer’s protocol, including the optional blocking, to couple magnetic beads (MagPlex, Luminex) to a panel of 96 analytes including the following commercially available proteins (with 1.5 × 10^6^ beads to 3 μg of the proteins not provided as lysates): IFN-α2, IFN-α1, IFN-α7, IFN-α14, IFN-β1, IFN-ε, IFNω-1, IFN-α5, IL-22, IFN-α6, IFN-α10, IFN-α8, IFN-α16, IFN-α17, IFN-κ, anti-IgG, IFN-α21, IL-17A, TROVE2, RBM38, IFN-α4, IL-17F, and ATP4A. The samples were diluted 1:25 in PBS and then 1:10 in assay buffer (0.05% PBS-T, 3% BSA, 5% milk). Stocks of magnetic beads were sonicated for 1 min before mixing with storage buffer from the activation kit. The diluted samples were centrifuged for 1 min at 3,000 rpm, and 45 μl of each sample was then incubated for 2 h in the dark at room temperature with 5 μl of stock bead solution, with shaking at 650 rpm. The beads were then washed (3 times with PBS-T 0.05%), centrifuged at 2,000 rpm, resuspended in 50 μl 0.2% PFA per well and carefully vortexed. After incubation for 10 min at room temperature and centrifugation at 2,000 rpm, the beads were washed (3 times with PBS-T 0.05%) and incubated with secondary antibodies (Invitrogen, H10104, 2384336) for 30 min at room temperature. Finally, the wash routine described above was repeated, and the beads were dispensed in PBS-T 0.05% before the Luminex FlexMap 3D read out.

### Screening for tissue-specific autoantibodies

Plasma samples obtained from patients with APS-1 (*n* = 31), p52^LOF^/IκBδ^GOF^ (*n* = 30) or p52^LOF^/IκBδ^LOF^ (*n* = 4) variants were analysed for the presence of specific autoantibodies in various immunological tests. The anti-tissue autoantibodies on rat tissue test (BioRad/Kallestad, 29020) and the anti-adrenal autoantibodies on primate tissue test (Inova, 508375) were performed using commercially available slides. The detection of anti-intrinsic factor (Thermo Fisher Scientific, Phadia, 14-5668-01), anti-thyroperoxydase (Thermo Fisher Scientific, Phadia, 14-5641-01) and anti-thyroglobulin (Thermo Fisher Scientific, Phadia, 14-5642-02) antibodies was performed using the ELiA technique; the presence of anti-IA2 (Theradiag, 10513417) or anti-21-OH (Theradiag, RL21E/96D) autoantibodies were assessed by ELISA. All of the test procedures were performed once and conducted according to the protocols provided by the kit manufacturers.

### Microbiological investigations

The normalized viral load was determined for each sample, by determining the viral load for 1 million cells in the nasopharyngeal swabs by quantitative PCR with reverse transcription using the SARS-CoV-2 R-gene kit (bioMérieux). In brief, nucleic acids were extracted from 0.2 ml nasopharyngeal swab (NPS) with NUCLISENS easyMAG and amplification was performed using the Bio-Rad CFX96 instrument. The viral load was determined with four internally developed quantification standards (QSs) targeting the SARS-CoV-2 *N* gene: QS1 to QS4, at 1 × 10^5^, 1 × 10^4^, 1 × 10^3^ and 1 × 10^2^ copies per µl, respectively, of a SARS-CoV-2 DNA standard. These QSs were controlled and quantified using a Nanodrop spectrophotometer (Thermo Fisher Scientific) and Applied Biosystems QuantStudio 3D Digital PCR. In parallel, NPS were tested using the CELL Control R-GENE kit (amplification of the *HPRT1* housekeeping gene; bioMérieux), which contains two quantification standards, QS1 and QS2, at 10^4^ copies per µl (50,000 cells per PCR in our conditions) and 10^3^ copies per µl (5,000 cells per PCR) of DNA standard, respectively, to normalize the viral load according to the amount of sample. Normalized viral load was calculated as log_10_[copies per 10^6^ cells]. Potential co-infections were investigated using the BioFire Respiratory 2.1 plus Panel (RP2.1plus) detecting 23 respiratory pathogens, including SARS-CoV-2 (bioMérieux).

### Blood and nasal IFN score determination

Total RNA was extracted from whole blood into PAXgene tubes using the Maxwell16 LEV simplyRNA Blood kit (Promega) according to the manufacturer’s instructions. Blood IFN score was determined using Nanostring technology as previously described^67^. For the nasal IFN score, we tested 100 μl nasal pharyngeal swab samples with the IFN prototype, as previously described^17^. The first prototype of the IFN pouch encompasses four ISGs (IFNα-inducible protein 27, IFI44L, IFN-induced protein with tetratricopeptide repeats 1, radical *S*-adenosyl methionine domain containing 2) and three housekeeping genes (hypoxanthine phosphoribosyltransferase 1, peptidylprolyl isomerase B and 2,4-dienoyl-CoA reductase 1) for signal normalization. In brief, the pouches were hydrated with the hydration solution. The PAXgene blood or nasal pharyngeal swab samples were mixed with 800 μl of the sample buffer provided with the kit and injected directly into the pouch and run on FilmArray 2.0 and FilmArray Torch instruments (BioFire Diagnostics). Results were delivered within 1 h. Using a research version of the instrument, we determined the real-time quantification cycle values and post-amplification melt peaks for each assay. The normalized expression values for each assay were then calculated with the internal reference genes. Nasal pharyngeal ISG score was calculated using the same method as for PAXgene samples, as previously described^67^.

### CyTOF

The whole-blood mass cytometry panels used were custom produced, and their contents are shown in Supplementary Table 8. Labelled cells were frozen at −80 °C after overnight dead-cell staining, and acquisition was performed on the Helios machine (Fluidigm). All of the samples were processed within 48 h of sampling. The six patients with autosomal-recessive APS-1 included in the analysis were on treatment with JAK inhibitors at the time of blood collection. Data analysis was performed using OMIQ software. The gating strategy for CyTOF immunophenotyping is shown in Supplementary Fig. 15.

### Immunostaining of human thymus sections

Thymic biopsy samples were collected from a patient with complete autosomal-recessive RELB deficiency (mutation Y397*/Y397*, P2 from ref. ^59^) and a deceased patient with a p52^LOF^/IκBδ^GOF^ variant (P850Sfs*36/WT from ref. ^68^). Tissues were fixed in 4% paraformaldehyde (Thermo Fisher Scientific), washed with PBS and embedded in paraffin. Antigen retrieval was performed on rehydrated tissue by boiling sections in Citra antigen retrieval solution (Biogenex). The sections were blocked by incubation for 30 min at room temperature in CAS-Block (Thermo Fisher Scientific) plus 0.2% Triton X-100 (Sigma-Aldrich), and were then incubated overnight at 4 °C with primary antibodies. The sections were washed with PBS-Tween 0.1% and stained by incubation with a biotinylated secondary antibodies for 1 h at room temperature for AIRE visualization. When necessary, secondary antibody staining was performed at room temperature for 1 h. The sections were washed with PBS-Tween 0.1% and mounted in ProLong Diamond Antifade mounting solution (Thermo Fisher Scientific). Images were acquired on an Apotome microscope (Zeiss). The antibodies used were K8-Alexa647, Rb (EP1628Y), Abcam, ab192468, 1:300; K5 Alexa488, Rb (EP1601Y), Abcam, ab193894, 1:300; AIRE, rat, eBioscience, 14-9534-82, 1:50; pan-keratin, Rb, Abcam, ab9377, 1:200; K10 Alexa647, Rb (EP1607IHCY), Abcam, ab194231, 1:300; UEA-1 biotinylated, Vector Laboratories, B-1065-2, 1:500.

### Mice

*Nfkb2*^+/Y868*^ NOD mice were generated by the Genetics Core Facility at National Jewish Health, Denver Colorado. Both WT littermate control and *Nfkb2*^+/Y868*^ NOD mice were maintained in specific-pathogen-free facilities at the University of California San Francisco (UCSF) in accordance with the guidelines established by the Institutional Committee on Animal Use and Care (IACUC) and Laboratory Animal Resource Center (LARC). Animal procedures were approved by the IACUC and LARC at UCSF, where mice aged 8–12 weeks, matched for age and sex, were used for tissue collection.

### Mouse mTEC isolation and flow cytometry

A previously established mouse thymus tissue-processing and single-cell-isolation protocol was used for flow cytometry analysis^69^. Single-cell suspensions were incubated with Live/Dead Fixable Blue Dead Cell Stain (Thermo Fisher Scientific) in 1× PBS for 15 min at 4 °C and then washed in PBS. They were blocked by incubation with anti-mouse CD16/CD32 (24G2) antibodies (UCSF Hybridoma Core Facility) for 15 min at 4 °C before cell surface marker staining in FACS buffer for 30 min at 4 °C. Cells were fixed and permeabilized with the FOXP3 staining buffer kit (eBioscience) according to the manufacturer’s protocol for intracellular protein staining. Flow cytometry data were collected on the LSRII Flow Cytometer (BD Biosciences) and analysed using FlowJo v.10.8.1. Antibodies against the following proteins were used: AIRE (5H12, eBioscience, 53593482), CD45 (30-F11, BioLegend, 103130), EPCAM (G8.8, BioLegend, 118218), I-Ak (10-3.6, BioLegend, 109908).

### Immunostaining of mouse thymus sections

Mouse thymuses were fixed by incubation in 2% paraformaldehyde (Thermo Fisher Scientific, 28908) in PBS for 2 h at room temperature and were then incubated overnight at 4 °C in 30% (w/v) sucrose (Sigma-Aldrich, S7903-1KG) in PBS. Tissues were embedded in Optimal Cutting Temperature Compound (Tissue-Tek 4583) and stored at −80 °C until sectioning (30–50 μm) on a Cryostat (Leica). Tissue sections on slides were rehydrated in PBS for 5 min before permeabilization in 0.3% Triton X-100 (Sigma-Aldrich), 0.2% BSA (Sigma-Aldrich) and 0.1% sodium azide (Sigma-Aldrich) in PBS with shaking for 45 min at room temperature. The sections were blocked by incubation with BlockAid (Thermo Fisher Scientific, B10710) at room temperature for 1 h. The sections were stained by incubation with primary fluorophore-conjugated antibodies for 1 h at room temperature and washed in 1× PBS. The sections were then stained with DAPI (BioLegend, 422801) for 5 min at room temperature followed by three washes in 1× PBS. Antibodies against the following proteins were used at a dilution of 1:200: AIRE (5H12, eBioscience, 53593482), K5 (EP1601Y, Abcam, 193895), K10 (EP1607IHCY, Abcam, 194231). All of the tissue sections were mounted in ProLong Diamond (Thermo Fisher Scientific) mounting medium. Images were captured on the Leica SP8 (Leica) laser-scanning confocal microscope.

### Histology

Organs from age- and sex-matched *Nfkb2*^*+/+*^ and *Nfkb2*^*+/*Y868*^ mice (10 to 11 weeks old) were collected and fixed overnight in 10% formalin, and shipped in 70% ethanol to HistoWiz for sectioning, and staining for haematoxylin and eosin. Immune infiltrates of tested organs were confirmed with a blinded observer.

### PhIP–seq with a mouse proteome library

The mouse proteome T7 phage-display library, which was described elsewhere^70^, was used for immunoprecipitation and sequencing to identify autoreactivities. Serum samples from *Rag2*-KO (*n* = 5), WT NOD (*n* = 8), *Aire*-KO NOD (*n* = 8) and *Nfkb2*^+/Y868*^ NOD (*n* = 8) mice were used in a previously published high-throughput protocol^70^. We analysed peptide enrichment after PhIP–seq by aligning reads at the protein level with RAPsearch as previously described^70^. Aligned reads were normalized to 100,000 reads per *k*-mer (RPK) to account for variable read depth, and log_2_-transformed fold changes in read counts were calculated for each sample relative to the mean number of read counts in mock immunoprecipitations and *Rag2*-KO mice. Peptides with *z*-scores greater than or equal to 3 were considered to be hits, and peptides displaying enrichment in mutant mice were identified as peptides classified as hits in at least three mutant mice and no WT mice. Peptides were labelled with the corresponding protein.

### RNA extraction, sequencing and analysis

Total RNA was isolated from whole blood as previously described^67^. RNA sequencing was performed using the Illumina NovaSeq S2 instruments (2 × 100 bp), at a read depth of 70 million. Single samples were sequenced across two lanes, and the resulting FASTQ files were merged by sample. All FASTQ files passed quality control and the sequences were aligned with the GRCh38 reference genome using STAR (v.2.6.1d). BAM files were converted to a raw count expression matrix using featurecount. Raw count data were normalized using DEseq2 (v.1.40.2). The ensemble IDs targeting multiple genes were collapsed (average), and a final data matrix gene was generated for single-gene set enrichment analysis with the BloodGen3Module gene set^71^. Statistical analysis was performed on a predefined gene set. Specifically, we used a fixed repertoire of 382 blood transcriptional modules that were thoroughly annotated and characterized functionally as described previously^71^. In brief, this repertoire of transcriptional modules (BloodGen3) was identified on the basis of co-expression, as measured in a collection of reference blood transcriptome datasets encompassing 16 pathological or physiological states and 985 individual transcriptome profiles. Sets of co-expressed transcripts were derived from a large weighted coclustering network in which edges represented the number of times a pair of genes coclustered in the 16 reference datasets (with a weight of 1 to 16). We calculated an IFN module enrichment score for individual samples by performing single-sample gene set enrichment analysis (ssGSEA) (GSVA package v.1.48.3), with the six IFN-response modules of the BloodGen3Module gene set (1.8.0), aggregate A28 as input. The enrichment scores of individual samples were used for heat-map visualization.

### Thymus CT scan

We performed a retrospective assessment of the thymus for those patients for whom a chest CT scan was available. For patients with several scans, we selected the first scan or the scan on which the thymus was largest. Most of the patients’ scans were performed without contrast injection, and the thymic margins were assessed by multiplanar reconstruction. The thymus was measured in three planes: thickness and width in the axial plane through the aortic arch, greatest height in a coronal or sagittal oblique plane. We established a control group matched for age (±1 month) and sex. Three controls were selected per patient. The control group was randomly selected from scans performed at our centre for polytrauma, excluding severe head trauma with coma or neurological disorders and thoracic trauma (so as not to alter mediastinal anatomic reports).

### Statistical methods

Data were analysed using GraphPad Prism software v.9.5.0 (GraphPad Software). The statistical significance of quantitative differences between groups was assessed in two-tailed unpaired Mann–Whitney *U*-tests. The statistical significance of differences between two groups in mouse studies was calculated using unpaired, parametric, two-tailed Student’s *t*-tests. For comparisons of more than two groups, the statistical significance of differences was calculated using two-way nonparametric ANOVA (Sidak’s test) with correction for multiple comparisons. Only statistically significant comparisons are indicated by their *P* values. All data are expressed as the mean ± s.d. calculated from at least three independent experiments unless otherwise stated.

### Ethics statement

Patients were included in the C18-41 Genetic Predisposition to Severe Infections study approved by the Sud Est II ethics committee (approval no. 2022-A00257-36) in France. All of the enrolled participants provided written informed consent and were collected through protocols conforming to local ethics requirements. Ethics approval was obtained from the Comitato Etico Provinciale (NP 4000—Studio CORONAlab) in Brescia, Italy, the French Ethics Committee Comité de Protection des Personnes, Ile de France II (2010-A00634-35 protocol no. C10-13) and the Rockefeller University Institutional Review Board in New York (protocol no. JCA-0700).

### Reporting summary

Further information on research design is available in the Nature Portfolio Reporting Summary linked to this article.

## Online content

Any methods, additional references, Nature Portfolio reporting summaries, source data, extended data, supplementary information, acknowledgements, peer review information; details of author contributions and competing interests; and statements of data and code availability are available at 10.1038/s41586-023-06717-x.

### Supplementary information

Supplementary Information Supplementary Results 1–4, providing additional information regarding the functional characterization of three types of inborn errors of NF-κB2, immunological and clinical description of the cohort, and Supplementary References.

Reporting Summary

Supplementary Figure 1 Uncropped images from the western blots displayed in the indicated figures.

Supplementary Tables Supplementary Tables 1–8.

### Source data

Source Data Fig. 5 and Source Data Extended Data Fig. 10

## Data Availability

All the data supporting the findings of this study are available within the Article and its Supplementary Information. The gel source data are shown in Supplementary Fig. 1. The RNA-seq data generated in this study have been deposited in the NCBI database under NCBI-SRA project PRJNA989123. All the other data and material supporting the findings of this study are available under a data transfer agreement from the corresponding authors on reasonable request. Source data are provided with this paper.
