# Supplementary material for: Autoantibodies against type I IFNs in humans with alternative NF-κB pathway deficiency
Source: Nature. 2023 Nov 8;623(7988):803–13. doi: 10.1038/s41586-023-06717-x (PMC10665196; doi:10.1038/s41586-023-06717-x)
Supplement: Supplementary file 1 — Supplementary Results 1–4, providing additional information regarding the functional characterization of three types of inborn errors of NF-κB2, immunological and clinical description of the cohort, and Supplementary References. [file 41586_2023_6717_MOESM1_ESM.pdf]

nature portfolio

https://doi.org/10.1038/s41586-023-06717-x

Supplementary information

Autoantibodies against type I IFNs in humans with alternative NF-κB pathway deficiency

In the format provided by the authors and unedited

Supplementary Information

Autoantibodies against type I IFNs in humans with alternative NF-κB pathway deficiency

Tom Le Voyer, Audrey V. Parent, Xian Liu, Axel Cederholm, Adrian Gervais, Jérémie Rosain, Tina Nguyen, Malena Perez Lorenzo, Elze Rackaityte, Darawan Rinchai, Peng Zhang, Lucy Bizien, Gonca Hancioglu, Pascale Ghillani-Dalbin, Jean-Luc Charuel, Quentin Philippot, Mame Sokhna Gueye, Majistor Raj Luxman Maglorius Renkilaraj, Masato Ogishi, Camille Soudée, Mélanie Migaud, Flore Rozenberg, Mana Momenilandi, Quentin Riller, Luisa Imberti, Ottavia M. Delmonte, Gabriele Müller, Baerbel Keller, Julio Orrego, William Alexander Franco Gallego, Tamar Rubin, Melike Emiroglu, Nima Parvaneh, Daniel Eriksson, Maribel Aranda-Guillen, David I. Berrios, Linda Vong, Constance H. Katelaris, Peter Mustillo, Johannes Raedler, Jonathan Bohlen, Jale Bengi Celik, Camila Astudillo, Sarah Winter, NF-κB consortium, COVID Human Genetic Effort, Catriona McLean, Aurélien Guffroy, Joseph L. DeRisi, David Yu, Corey Miller, Yi Feng, Audrey Guichard, Vivien Béziat, Jacinta Bustamante, Qiang Pan-Hammarström, Yu Zhang, Lindsey B. Rosen, Steve M. Holland, Marita Bosticardo, Heather Kenney, Riccardo Castagnoli, Charlotte A. Slade, Kaan Boztuğ, Nizar Mahlaoui, Sylvain Latour, Roshini S. Abraham, Vassilios Lougaris, Fabian Hauck, Anna Sediva, Faranaz Atschekzei, Georgios Sogkas, M. Cecilia Poli, Mary A. Slatter, Boaz Palterer, Michael D. Keller, Alberto Pinzon-Charry, Anna Sullivan, Luke Droney, Daniel Suan, Melanie Wong, Alisa Kane, Hannah Hu, Cindy Ma, Hana Grombiříková, Peter Ciznar, Ilan Dalal, Nathalie Aladjidi, Miguel Hie, Estibaliz Lazaro, Jose Franco, Sevgi Keles, Marion Malphettes, Marlene Pasquet, Maria Elena Maccari, Andrea Meinhardt, Aydan Ikinciogullari, Mohammad Shahrooei, Fatih Celmeli, Patrick Frosk, Christopher C. Goodnow, Paul E. Gray, Alexandre Belot, Hye Sun Kuehn, Sergio D. Rosenzweig, Makoto Miyara, Francesco Licciardi, Amélie Servettaz, Vincent Barlogis, Guillaume Le Guenno, Vera-Maria Herrmann, Taco Kuijpers, Grégoire Ducoux, Françoise Sarrot-Reynauld, Catharina Schuetz, Charlotte Cunningham-Rundles, Frédéric Rieux-Laucat, Stuart G. Tangye, Cristina Sobacchi, Rainer Doffinger, Klaus Warnatz, Bodo Grimbacher, Claire Fieschi, Laureline Berteloot, Vanessa L. Bryant, Sophie Trouillet Assant, Helen Su, Benedicte Neven, Laurent Abel, Qian Zhang, Bertrand Boisson, Aurélie Cobat, Emmanuelle Jouanguy, Olle Kampe, Paul Bastard, Chaim M. Roifman, Nils Landegren, Luigi D. Notarangelo, Mark S. Anderson, Jean-Laurent Casanova and Anne Puel

The Supplementary Information file contains:

- **Supplementary Results** 1-4 (providing additional information regarding the functional characterization of three types of inborn errors of NF-κB2, immunological and clinical description of the cohort)
- **Supplementary Figures** 1-11 (uncropped Western Blots and additional results)
- **Supplementary References**
- **Supplementary Tables** 1-8
- **Source Data** for Figure 5b,d; Extended Data Figure 10d,h,i,j,k.

Supplementary results 1: Functional characterization of three types of AD inborn errors of NF-κB2

A cohort of patients with inborn errors of the alternative NF-κB pathway

We investigated a cohort of inborn errors of the alternative NF-κB pathway comprising 73 patients from 50 kindreds heterozygous for 28 different rare (MAF< 0.0001) non-synonymous *NFKB2* variants (**Extended Data Fig. 1a,b and Supplementary Table 1**). This cohort included 29 patients from 18 families with 12 *NFKB2* variants reported in previous studies (**Supplementary Table 1**). The condition was familial in 50 patients from 27 kindreds, and sporadic in 19 patients (unknown in 4 patients) (**Extended Data Fig. 1a and Supplementary Table 1**). Consistent with AD inheritance, *NFKB2* is under strong negative selection, with a CoNeS score[1] of -1.6 and a high probability of being loss-of-function-intolerant (pLI of 1) (**Extended Data Fig. 1c and Extended Data Fig. 2a**). Nineteen variants (68%) clustered in a region corresponding to the C-terminal domain of the protein (CTD, aa 760-900) **(Fig. 1a)**. These variants comprised 13 predicted loss-of-function (pLOF) variants, and six missense variants, five of which were predicted to affect the NRS (aa 861-871). This region, which is crucial for NIK-mediated phosphorylation and the processing of p100 to generate p52, is under particularly strong purifying selection, with a low missense tolerance ratio (MTR) score (**Extended Data Fig. 2b**). We found that 59 patients from 40 kindreds carried 19 heterozygous variants in the CTD (pLOF, *n*=40 from 29 kindreds or missense, *n*=19 patients from 11 kindreds), including 20 individuals from 14 kindreds with the recurrent R853* variant (**Fig. 1a, Supplementary Table 1**). The recurrence of this variant probably reflects the existence of a mutational hotspot, as opposed to a founder effect, because this variant was previously detected in patients from nine different countries and was shown to have occurred *de novo* in six patients[2–7]. The other 13 patients were heterozygous for *NFKB2* variants in the

Rel homology domain (RHD) (*n*=5 from 4 kindreds; pLOF, *n*=4 and missense, *n*=1), or the ankyrin repeat domain (ARD) (pLOF, *n*=6 patients from 3 kindreds, and missense, *n*=3 from 3 kindreds) (**Fig. 1a and Extended Data Fig. 1a**). The consequences of the c.104-1G>C/WT *NFKB2* variant of patient P63, predicted to disrupt an essential acceptor splicing site, were evaluated by TOPO TA cloning of cDNA from T-cell blasts. About half the transcripts were abnormally spliced, mostly by a skipping of exon 4, and were predicted to encode a truncated protein (A35Efs*10), confirming the detrimental effect of the c.104-1G>C variant on the splicing of the *NFKB2* mRNA (**Extended Data Fig. 2c**). We also enrolled 14 patients with other inborn errors of the alternative NF-κB pathway (AR NIK (*n*=2) and AR RelB (*n*=8) deficiencies) or upstream receptors (AR BAFF (*n*=1) or X-linked (XL)-CD40L (*n*=3) deficiencies) (**Extended Data Fig. 2d,e and Supplementary Table 2**).

Luciferase reporter assay assessing the p52-dependent transcriptional activity of the *NFKB2* alleles

NF-κB2 (the corresponding *NFKB2* gene encodes both p100 and its cleaved product, p52) is one of the five REL/NF-κB proteins. The active form, p52 can bind DNA, but, like NF-κB1 (p105/p50), and unlike RelA, RelB, and cRel, it lacks a transcriptional activation domain (TAD). Its transcriptional activation, thus, requires heterodimerization with another TAD-containing REL protein[8]. The p52 protein predominantly dimerizes with RelB *in vivo*, but an excess of p52 can lead to p52/p52 homodimerization, potentially repressing the κB-site transcriptional activity of other dimers though a dosage effect, as indicated by *in vitro* studies[9,10] (**Extended Data Fig. 3a, left panel**). Twenty-three *NFKB2* variants have been reported in more than 80 patients with AD NF-κB2 disorders, but only five of these variants have been tested functionally. The E418* and R635* variants result in spontaneous nuclear translocation, whereas the R853*, S866R and A867Cfs*19 variants prevent the processing of p100 to p52[11–

[13]. However, these assays cannot distinguish between the two functions (p52 and IκBδ) of the proteins encoded by the *NFKB2* locus. We therefore developed an assay for evaluating the functional impact of *NFKB2* variants by assessing p52-dependent κB luciferase activity, 24 and 48 h after the co-transfection of HEK293T cells with plasmids encoding NIK, RelB, and/or NF-κB2/p100 (**Extended Data Fig. 3a, middle panel**). Transfection with NIK alone or together with RelB led to strong RelA-dependent κB transcriptional activity in HEK293T cells, 24 to 72 hours after transfection, as only a weak luciferase signal was detected in *RELA*-deficient HEK293T cells (**Extended Data Fig. 3b**). With this assay, we were, therefore, able to assess the p52-dependent capacity of the NF-κB2 variants to bind the κB promoter and to prevent the transcriptional activity induced by endogenous RelA-containing dimers [9] (**Extended Data Fig. 3a, right panel**). For the validation of this test, we generated plasmids encoding previously characterized biochemical NF-κB2 mutants (**Extended Data Fig. 3c**), including a DNA binding-deficient mutant (R52A-R54A)[14], a dimerization-defective NF-κB2 mutant (Y247A) shown to prevent heterodimerization with RelB or RelA and subsequent processing into p52[15,16], two processing-resistant NF-κB2 mutants (S866A and S870A)[16–18] and a plasmid encoding the C-terminal region of NF-κB2 ($p100^{405\text{-}900}$). Cotransfection with WT *NFKB2* (p100) repressed the κB transcriptional activity induced by NIK and RelB, 24 h and 48 h post-transfection (**Fig. 1b,** black bars and **Extended Data Fig. 3d**, black bars). By contrast, under the same conditions, the repression of κB transcriptional activity by R52A-R54A, Y247A, S866A, S870A, or $p100^{405\text{-}900}$ was impaired, indicating that these variants were $p52^{LOF}$ relative to the WT NF-κB2[17,19,20] (**Fig. 1b and Extended Data Fig. 3d**, colored bars). Mutants truncated within the ARD ($p100^{1\text{-}455}$ or $p100^{1\text{-}665}$) had an enhanced capacity to repress κB transcriptional activity due to the absence of the last ankyrin (ANK) repeats in the ARD and the PID (aa 761-851); these variants were, therefore, p52-gain-of-function ($p52^{GOF}$), 24 h and/or 48 h after transfection [17] (**Fig. 1b and Extended Data Fig. 3d**, colored bars). Biochemical mutants lacking the PID

but with all the ANK repeats intact (p100$^{1-750}$, p100$^{1-776}$), and variants in which the ubiquitinylation residue (K855R) was mutated displayed a partial impairment of repression, at 48 h after transfection (**Fig. 1b and Extended Data Fig. 3d**, colored bars).

NFKB2 variants can be LOF or GOF in terms of p52/p52 homodimer function

We used this assay to evaluate the impact of 26 *NFKB2* variants from our cohort of patients, and the 13 additional previously reported variants[21] (**Fig. 1a**). The four RHD pLOF variants (c.104-1G>C/A35Efs*10, R52*, W270*, and K321Sfs*160 (referred to hereafter as K321Sfs)) were p52^{LOF} (**Fig. 1c and Extended Data Fig. 3d**). The E418* pLOF variant was p52^{GOF}, as previously reported[11], 24 h but not 48 h after transfection. The four ARD pLOF variants lacking the PID[22] (L531Cfs*5, Q539*, R611*, and R635*) were p52^{GOF}. All the CTD pLOF (*n*=17, from S762Afs*21 to Q871*) and missense variants located within the NRS (*n*=6, from D865G to S870N) were p52^{LOF} (**Fig. 1c and Extended Data Fig. 3d**). All seven additional missense variants (K321E, G369R, P491S, A567V, V661M, P681L, and V844A) reported in patients with PAD (probably due to another genetic lesion, referred to hereafter as 'idiopathic PAD') were localized outside of the NRS and were isomorphic in terms of p52 inhibitory function (p52^{WT}) 24 h and 48 h after transfection (**Supplementary Fig. 2a,b, left panels)**. Finally, we tested the 14 missense variants with a MAF > 10^{-4} reported in the heterozygous state in the gnomAD and BRAVO public databases (**Extended Data Fig. 2a**). All these variants were p52^{WT} 24 h and 48 h after transfection (**Supplementary Fig. 2a,b, right panels**). Overall, our findings show that the deleterious *NFKB2* variants found in patients with PAD can be GOF or LOF in terms of the repression of transcription activity by p52/p52 homodimers. Indeed, pLOF variants within the RHD and CTD, or missense variants within the NRS are p52^{LOF}; pLOF variants impairing the ARD (E418* and pLOF variants within the ARD) are p52^{GOF}, whereas the missense variants reported in patients with PAD (K321E, G369R, P491S, A567V, V661M,

P681L, and V844A) and variants found in public databases with a MAF > 10^{-4} are p52^{WT}. We infer from these data that the transcriptional activity of p52-RelB heterodimers may follow the same pattern.

The p52^{LOF} variants of the CTD resist the NIK-dependent processing of p100

The processing of p100 to p52 is dependent on the phosphorylation of S866 and S870 by NIK[17]. We assessed the impact of *NFKB2* variants on p100 activation, by analyzing the phosphorylation of this protein at position S866 (P-p100), using an antibody recognizing the phosphorylated serine 866[18], and its proteolytic cleavage to generate p52, following cotransfection with NIK[17]. The Y247A, S866A and S870A mutants abolished p100 processing, impairing (Y247A and S870A) or abolishing (S866A) phosphorylation of the S866 residue of p100 at 48 h (**Extended Data Fig. 3c**). An abolition of p100 phosphorylation was observed for the truncated p52^{GOF} mutants (p100$^{1-455}$, p100$^{1-665}$). After 48 h of transfection, the p52^{LOF} W270* and K321Sfs variants of the RHD, and the E418* and the other p52^{GOF} variants of the ARD (L531Cfs*5, Q539*, R611*, and R635*) had produced a truncated protein, without the generation of phosphorylated p100 or p52 following cotransfection with NIK, contrasting with the results for the WT *NFKB2* cDNA (**Extended Data Fig. 4a**)[11]. The 23 p52^{LOF} variants of the CTD tested (17 pLOF and 6 missense variants of the NRS) presented an impairment (Q871* and S870N) or abolition (the other 21 variants) of p100 phosphorylation, and all were resistant to p100 processing, with no p52 production following cotransfection with NIK (**Extended Data Fig. 4a**). As expected, the 10 most frequent missense variants with a MAF>10^{-4} from public databases shown above to be p52^{WT} in terms of the repression of transcriptional activity had normal P-p100 levels and p100 processing capacities (**Supplementary Fig. 2c**). Overall, these findings suggest that the deleterious *NFKB2* alleles may be p52^{LOF} due to an impairment of normal p52 expression, because the p52 mutant protein is truncated or because p52 generation

is impaired by resistance to p100 processing. Alternatively, they may be p52^{GOF}, possibly due to constitutive p52 translocation and DNA-binding activity in the absence of a functional PID (**summarized in Supplementary Fig. 3 and Supplementary Table 3**).

The processing-resistant p100 variants have enhanced IκBδ inhibitory capacities for repressing RelA-dependent canonical NF-κB activation

Unprocessed p100 can inhibit NF-κB dimers by preventing their nuclear translocation (IκBδ activity)[23]. We therefore tested the hypothesis that monoallelic processing-resistant CTD variants may have IκBδ GOF activity (p52^{LOF}/IκBδ^{GOF}) because their C-terminal region cannot be degraded by NIK, unlike that of the WT protein or variants in which the RHD or ARD (predicted IκBδ^{LOF}) is affected. The transfection of HEK293T cells with plasmids encoding NIK plus WT-NF-κB2 (aa 1-900), p52^{GOF} R611*, or the p52^{LOF} processing-resistant variants (R853* or S866N) resulted in similar levels of repression of κB transcriptional activity, suggesting that this test evaluates the two repressive functions of NF-κB2 (p52-dependent, through p52/p52 homodimer DNA-binding, and IκBδ-dependent, through cytoplasmic retention of the REL-containing NF-κB complexes) *in vitro* (**Supplementary Fig. 4a,b**). In these conditions, W270* was null, consistent with its lack of both p52 and IκBδ functions (**Supplementary Fig. 4a,b**). We specifically evaluated the capacity of the IκBδ of the WT and mutant NF-κB2/p100 proteins to inhibit RelA-dependent transcriptional activity, by cotransfecting cells with a plasmid encoding NIK together with a plasmid encoding only the C-terminal WT or mutant region of NF-κB2 (p100-Cter^{WT}, aa 405-900). The p100-Cter^{WT} protein had no transcriptional repression activity in this system, consistent with its sensitivity to proteasomal degradation upon cotransfection with NIK (IκBδ^{WT}) (**Extended Data Fig. 4b**). As expected, the Cter^{R611*} (aa 405-611) protein did not repress κB transcriptional activity (IκBδ^{LOF}), confirming the specificity of this assay for evaluating NF-κB2 IκBδ function. By contrast, the

$Cter^{R853*}$ (aa 405-853) and $Cter^{S866N}$ (aa 405-900) mutants, which were insensitive to proteasomal degradation upon cotransfection with NIK, strongly repressed κB transcriptional activity ($IκBδ^{GOF}$) (**Extended Data Fig. 4b**). For confirmation of the $IκBδ^{GOF}$ activity of the R853* and S866N variants, we used the dimerization-defective and processing-resistant NF-κB2 mutant (Y247A) to create double mutants. Co-transfection with NIK and a single mutant ($p100^{Y247A}$), or with $p100^{R853*/Y247A}$ or the $p100^{S866N/Y247A}$ double mutant resulted in an enhanced capacity to repress κB transcriptional activity relative to transfection with NIK alone, or with $p100^{W270*/Y247A}$ or $p100^{R611*/Y247A}$ **(Extended Data Fig. 4c).** Thus, processing-resistant CTD variants are $IκBδ^{GOF}$ ($p52^{LOF}/IκBδ^{GOF}$) in terms of transcriptional repression in the RelA-dependent canonical NF-κB pathway following cotransfection with NIK, whereas W270* and R611* are $IκBδ^{LOF}$ ($p52^{LOF}/IκBδ^{LOF}$ and $p52^{GOF}/IκBδ^{LOF}$, respectively).

The processing-resistant $IκBδ^{GOF}$ CTD NF-κB2 variants impair WT NF-κB2- and RelB-dependent alternative NF-κB activation

The activation of the alternative NF-κB pathway is dependent on the correct activation of the p52/RelB heterodimer. We therefore investigated whether $p52^{LOF}/IκBδ^{GOF}$ variants impaired the activation of WT NF-κB2 or RelB. Cotransfection with various amounts of the $p52^{LOF}/IκBδ^{GOF}$ R853* or S866N variants and constant amounts of NIK and WT p100 revealed a dose-dependent inhibition of WT p100 phosphorylation and, to a lesser extent, of its processing to generate p52 (**Extended Data Fig. 4d**). By contrast, increasing the amount of $p52^{LOF}/IκBδ^{LOF}$ W270* cDNA had no effect on WT P-p100 levels or WT p100 processing (**Extended Data Fig. 4d**). We then assessed the effects of the $p52^{LOF}/IκBδ^{GOF}$ variants on RelB and p52 nuclear translocation, by confocal microscopy in transiently transfected HeLa cells. Transfection with the $p52^{LOF}/IκBδ^{LOF}$ K321Sfs or $p52^{GOF}/IκBδ^{LOF}$ R611* variant, with or without NIK, resulted in constitutive nuclear localization of the encoded protein, consistent

with the absence of a nuclear export sequence (NES) [23] (**Fig. 1d and Supplementary Fig. 4c**). Transfection with RelB, either alone or together with the IκBδ^{LOF} K321Sfs or R611* variant, led to the translocation of RelB to the nucleus (**Fig. 1d**, left panel). By contrast, transfection with RelB plus WT p100, R853*, or S866N, in the absence of NIK, resulted in the retention of RelB in the cytoplasm, consistent with the functional p100-IκBδ activity of these proteins (**Fig. 1d**, left panel). The addition of NIK to this system led to RelB translocation to the nucleus after cotransfection with WT-p100, but not after cotransfection with the IκBδ^{GOF} R853* or S866N protein (**Fig. 1d**, right panel). Similar results were obtained with plasmids encoding the WT or mutant C-terminal domain of NF-κB2 (**Supplementary Fig. 4d**). Overall, our findings suggest that the p52^{LOF}/IκBδ^{GOF} mutant alleles block the activation of the alternative NF-κB pathway induced by NIK through their enhanced IκBδ function, by preventing the phosphorylation of WT-p100 and its processing into p52, and the nuclear translocation of RelB-containing dimers.

The p52^{LOF}/IκBδ^{GOF} variants impair p100 processing in heterozygous fibroblasts

NF-κB2 is strongly expressed in stromal (e.g. lymph node and mTEC) and hematopoietic (e.g. B cells) cells[24]. We investigated the consequences of the p52^{LOF}/IκBδ^{GOF} variants in stromal (primary and SV40-immortalized (SV40) fibroblasts) and hematopoietic (T-cell blasts and monocyte-derived dendritic cells (MDDCs)) cells from patients following the activation of the alternative NF-κB pathway by lymphotoxin (Lt), TWEAK (fibroblasts), or CD40L (leukocytes)[25,26]. Cells heterozygous for the p52^{LOF}/IκBδ^{GOF} R853* allele produced a truncated protein, whereas cells heterozygous for the p52^{LOF}/IκBδ^{LOF} c.104-1G>C/A35Efs*10 or K321Sfs variant produced no mutant protein detectable with an antibody binding to the N-terminus of the protein (**Extended Data Fig. 5a and Supplementary Fig. 5a-c**). Primary fibroblasts from a healthy donor stimulated with Lt for 48 h displayed p100 phosphorylation and processing to generate p52 (**Extended Data Fig. 5a**). By contrast, primary fibroblasts from

a patient heterozygous for the p52^{LOF}/IκBδ^{GOF} R853* variant presented an accumulation of the truncated p100 mutant protein after Lt stimulation, with lower levels of WT p100, P-p100 and p52 induction than WT cells or cells heterozygous for the p52^{LOF}/IκBδ^{LOF} K321Sfs variant (for P-p100 in particular). Similarly, primary fibroblasts from a patient with AR complete NIK deficiency (NIK$^{-/-}$, homozygous for P565R variant[27]) presented an accumulation of unprocessed p100 following Lt stimulation, with an abolition of p100 phosphorylation, and no p52 production, consistent with the essential role of NIK in p100 processing, whereas RelB-deficient fibroblasts (RelB$^{-/-}$, homozygous for Q72Tfs*152, *unpublished*) had low levels of P-p100 and p100 following stimulation with Lt, with a normal p100 processing capacity (**Extended Data Fig. 5a**). An accumulation of the truncated p100 mutant protein and impaired p100 processing into p52 relative to WT or K321Sfs/WT cells were also observed in SV40 fibroblasts (from R853*/WT, *n*=1) after Lt stimulation, and in MDDCs (from R848Efs*38/WT (*n*=1), R853*/WT (*n*=2) or A867V/WT (*n*=1) patients) 48 h after CD40L stimulation (**Supplementary Fig. 5a-c**). Given the impairment of p100 processing and the accumulation of the mutant p100 upon stimulation of the non-canonical pathway, the p100/p52 ratio was higher in cells from patients heterozygous for a p52^{LOF}/IκBδ^{GOF} variant (primary and SV40 fibroblasts and MDDCs) than in cells from healthy donors or the K321Sfs/WT patient (**Extended Data Fig. 5a and Supplementary Fig. 5a,c**).

The p52^{LOF}/IκBδ^{GOF} variants impair the translocation of p52- and RelB-containing dimers to the nucleus in heterozygous fibroblasts

We hypothesized that the heterozygous p52^{LOF}/IκBδ^{GOF} variants might impair the activation of p52/RelB heterodimers in the patients' cells. The activation of primary or SV40 control fibroblasts by incubation with TWEAK for 48 h induced the processing of p100 to generate p52, and the induction and translocation to the nucleus of RelB and p52 (**Extended**

Data Fig. 5b-d and Supplementary Fig. 6a). Almost no RelB or p52 translocation to the nucleus was detected after 48h of TWEAK stimulation in primary fibroblasts from an R853*/WT patient (**Extended Data Fig. 5c,d**). Similarly, no RelB or p52 was detected in the nucleus of primary fibroblasts from patients with AR complete NIK ($NIK^{-/-}$) or RelB ($RelB^{-/-}$) deficiency after 48h of TWEAK stimulation. By contrast, K321Sfs heterozygous primary fibroblasts displayed almost normal RelB induction and nuclear translocation after TWEAK stimulation (**Extended Data Fig. 5c,d**). RelB translocation was almost undetectable in SV40 fibroblasts from two unrelated R853*/WT patients or a $NIK^{-/-}$ patient, but was normal in cells heterozygous for the K321Sfs *NFKB2* variant (**Supplementary Fig. 6a**). Similar results were obtained in MDDCs from two healthy donors, and from patients heterozygous for the $p52^{LOF}/I\kappa B\delta^{LOF}$ K321Sfs or c.104-1G>T, or $p52^{LOF}/I\kappa B\delta^{GOF}$ R853* variants, after stimulation with CD40L (**Supplementary Fig. 6b**). Overall, these findings suggest that heterozygosity for a $p52^{LOF}$ mutant allele can cause NF-κB2 deficiency by two different mechanisms: (1) p52/p100 haploinsufficiency for $p52^{LOF}/I\kappa B\delta^{LOF}$ mutants, or (2) enhanced p100-IκBδ inhibitory function ($p52^{LOF}/I\kappa B\delta^{GOF}$) impairing the phosphorylation and processing of the WT p100, and preventing the translocation of p52/RelB heterodimers to the nucleus after activation of the alternative NF-κB pathway.

Supplementary results 2: Distinctive immunological phenotype of patients with $p52^{LOF}/I\kappa B\delta^{GOF}$ variants

Inborn errors of the alternative NF-κB pathway impair the development of B, T, and NK cells[21,27,28]. We assessed the immunological phenotype of patients with the three types of inborn errors of NF-κB2 ($p52^{LOF}/I\kappa B\delta^{LOF}$, $p52^{GOF}/I\kappa B\delta^{LOF}$, and $p52^{LOF}/I\kappa B\delta^{GOF}$). Most of these patients (aged 1-75 years) had low serum IgG, IgM and IgA concentrations, and few, if any, circulating B cells (39/53, 74%) (**Extended Data Fig. 6a-b**). We performed a deep

immunological analysis in 14 patients with inborn errors of *NFKB2* (p52^{LOF}/IκBδ^{GOF}, *n*=10, and p52^{LOF}/IκBδ^{LOF}, *n*=4) and six APS-1 patients, by cytometry by time of flight (CyTOF) on fresh whole-blood samples (**Fig. 2a**). Twelve of the 14 patients with inborn errors of NF-κB2 tested had low circulating B-cell counts and patients with p52^{LOF}/IκBδ^{GOF} variants had low proportions of switched memory B cells (**Fig. 2b,c and Extended Data Fig. 6b-d**). By contrast, APS-1 patients had normal blood B-cell counts and B-cell subset proportions. FlowSOM-guided unsupervised clustering showed that p52^{LOF}/IκBδ^{GOF} variants mostly affected the CD19^{+}CD27^{+} memory B-cell compartment, relative to healthy controls (HCs) and 3 patient with p52^{LOF}/IκBδ^{LOF} variants (*metaclusters* 1, 2, 3 and 10, $p < 0.05$) (**Fig. 2b,c and Extended Data Fig. 6c,d,e**). The numbers of total lymphocytes, CD4^{+} and CD8^{+} T cells were within the normal ranges in patients with p52^{LOF}/IκBδ^{GOF} variants (**Supplementary Fig. 7a**), whereas patients with p52^{LOF}/IκBδ^{LOF} or p52^{LOF}/IκBδ^{GOF} variants had counts at the lower end of the control range for γδ T cells. In analyses of T-cell subset proportions, patients with p52^{LOF}/IκBδ^{GOF} variants had more naïve CD4^{+} T cells, whereas patients with p52^{LOF}/IκBδ^{LOF} variants had lower proportions of naïve CD4^{+} T cells than controls (**Supplementary Fig. 7b**). The proportion of CD4^{+} recent thymic emigrant (RTE) cells, defined as CD4^{+}CD45RA^{-}CD31^{+} cells, was in the upper part of the normal range for age-matched controls in patients with p52^{LOF}/IκBδ^{GOF} variants, suggesting that thymic output was similar to or higher than that in patients with p52^{GOF}/IκBδ^{LOF} and p52^{LOF}/IκBδ^{LOF} variants[29] (**Supplementary Fig. 7b**). The absolute numbers and proportions of CD4^{+}CD25^{+}CD127^{-} regulatory T cells (Tregs) were significantly lower in patients with p52^{LOF}/IκBδ^{GOF} variants and in APS-1 patients, and to a lesser extent in patients with p52^{LOF}/IκBδ^{LOF} variants than in age-matched healthy donors (**Fig.2c and Supplementary Fig. 7c**). By contrast, four patients carrying the p52^{GOF}/IκBδ^{LOF} R635* allele (reported in[11]) had Treg proportions similar to those in age-matched controls (**Supplementary Fig. 7c)**. Patients with p52^{LOF}/IκBδ^{GOF} variants had lower proportions and

counts of $CD4^+CD45RA^+CXCR5^+$ T follicular helper (cTfh) cells than patients with $p52^{LOF}/I\kappa B\delta^{LOF}$ variants, APS-1 patients, and healthy donors, despite having normal memory $CD4^+$ T-cell counts (**Fig. 2c and Supplementary Fig. 7d**). Patients with $p52^{LOF}/I\kappa B\delta^{GOF}$ variants had slightly lower NK cell counts, but normal MAIT and iNKT cell counts (**Supplementary Fig. 7e**), and normal monocyte and dendritic cell counts or proportions (**Supplementary Fig. 7f**). Overall, patients heterozygous for a $p52^{LOF}/I\kappa B\delta^{GOF}$ variant had a distinctive immunological phenotype different from that of patients with $p52^{LOF}/I\kappa B\delta^{LOF}$ or $p52^{GOF}/I\kappa B\delta^{LOF}$ variants or with APS-1, including a characteristic combination of low levels of memory B cells, cTfh cells, and Tregs.

Supplementary results 3: Incomplete penetrance for the development of neutralizing auto-Abs against type I IFNs in patients carrying $p52^{LOF}/I\kappa B\delta^{GOF}$ variants

For 10 of the 57 (17%) patients carrying $p52^{LOF}/I\kappa B\delta^{GOF}$ variants, no auto-Abs capable of neutralizing IFN-α2 or IFN-ω at 100 pg/mL, or IFN-β (at 10 ng/mL) were detected. Plasma from these patients did not neutralize any of the 13 IFN-α subtypes at 1 ng/mL, contrasting with the findings for other patients with $p52^{LOF}/I\kappa B\delta^{GOF}$ variants, AR RelB, or AR NIK deficiency, whose plasma neutralized IFN-α2 at a concentration of 10 ng/mL (**Supplementary Fig. 8**). None of these patients reported severe or recurrent viral disease. Most of the patients without neutralizing auto-Abs against type I IFNs (AAN-I-IFNs) carried the A867V variant (*n*=7/10). They were from two countries (France, *n*=2 kindreds; Australia, *n*=2 kindreds) and were aged 11 to 51 years at testing. Three other patients carrying the same A867V variant had AAN-I-IFNs, with the auto-Abs from two of these patients neutralizing only IFN-ω at 100 pg/mL. Two additional unrelated patients carrying the recurrent R853* $p52^{LOF}/I\kappa B\delta^{GOF}$ variant (of the 18 patients with R853*), aged 17 and 61 years at testing, and one with the K855Sfs*7 variant (aged 47 years) did not have detectable AAN-I-IFNs. None of these patients received

immunosuppressive treatment. Age and sex ratio did not differ significantly between patients with p52^{LOF}/IκBδ^{GOF} variants with and without AAN-I-IFNs (female/male ratio of 1.2 (23/18) and 1 (5/5), respectively). These findings suggest that the penetrance of AAN-I-IFNs in p52^{LOF}/IκBδ^{GOF} variant carriers is high (~80%), but not complete by the age of 61 years, especially in patients heterozygous for the A867V variant.

Supplementary results 4: IFN-β-dependent ISG induction *in vivo* in patients with auto-Abs against IFN-α and/or IFN-ω

AAN-I-IFNs have been shown to impair the induction of ISGs in PBMCs and the nasal mucosa during COVID-19[30–32]. We monitored the disease caused by infection with the B.1.529 variant (omicron) of SARS-CoV2 in two patients (P27 and P28) carrying the R853Afs*30/WT p52^{LOF}/IκBδ^{GOF} variant (**Extended Data Fig. 9c**). Before infection, both patients had received three injections of Pfizer-BioNTech mRNA vaccine. One of these patients (P28) subsequently developed detectable IgG directed against the spike protein (anti-S) (**Extended Data Fig. 9d**). Both patients received recombinant IFN-β (as their auto-Abs did not neutralize IFN-β at 10 ng/mL) and anti-spike mAb (sotrovimab in P27 and tixagevimab/cilgavimab in P28) within four days of symptom onset (**Fig. 4g**). Both experienced a mild form of COVID-19 without pneumonia or a need for oxygen supplementation. We performed longitudinal assessments, from day 2 to day 27, of SARS-CoV-2 viral load and type I IFN response, by determining IFN score (quantifying four type I/III IFN-dependent ISGs with NanoString technology[32]) on whole blood and nasal swabs, and RNA-seq on whole blood (**Extended Data Fig. 9e**). Within the first few days of symptoms, infection with the SARS-CoV2 B.1.529 variant led to a high IFN score for whole blood and the upper respiratory tract in individuals with mild COVID-19 without AAN-I-IFNs (**Fig. 4f,g** and[32]**)**. By contrast, P27, whose plasma neutralized high concentrations of all 13 IFN-α subtypes plus IFN-ω, and P28, whose plasma neutralized high concentrations

of all 13 IFN-α subtypes but not IFN-ω, had negative or weakly positive blood and nasal IFN scores, respectively, despite having a nasal SARS-CoV-2 viral load similar to that in the individuals without auto-Abs (**Fig. 4f,g**). The neutralizing activity of the auto-Abs of P27 against IFN-α2 and IFN-ω was also demonstrated in the respiratory tract (**Extended Data Fig. 9f**). The injection of recombinant IFN-β from day 4 after the onset of the symptoms led to a high nasal and whole-blood IFN score, increasing to the levels observed in individuals with mild COVID-19 and no AAN-I-IFNs (**Fig. 4g**). RNA-seq on whole-blood samples from the two patients revealed an impairment of ISG induction four days after symptom onset relative to two age-matched controls with mild COVID-19 but similar SARS-CoV-2 viral loads (**Extended Data Fig. 9g,h and Supplementary Fig. 13)**. Three to four days after IFN-β and anti-spike mAb infusion, the ISG module scores increased, with the expression of these genes becoming undetectable by day 13 post-treatment, once viral replication was controlled (**Fig. 4g and Extended Data Fig. 9g,h)**. Thus, auto-Abs against IFN-α and IFN-ω can block type I IFN signaling *in vivo* in the blood and upper respiratory tract, and ISG induction can be rescued by exogenous IFN-β treatment, which may have contributed to the favorable clinical outcome in these patients.

Supplementary Figure 1: Uncropped images from the Western Blots displayed in the indicated figures (provided in a separated online file).

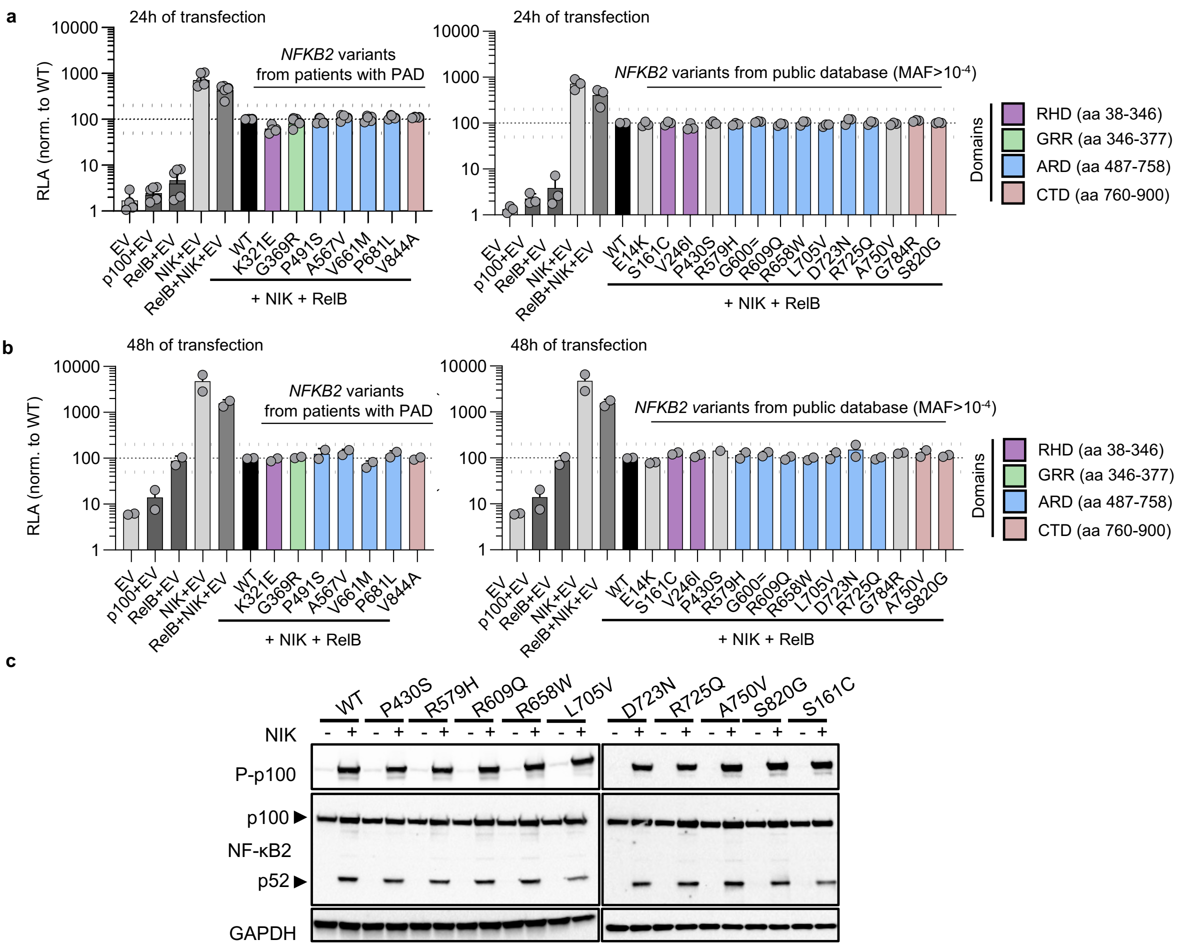

Supplementary Figure 2: Functional testing of *NFKB2* variants of patients with idiopathic PAD and from gnomAD. (**a-b**) RLA of HEK293T cells transfected with a κB reporter construct (κB-luc) in the presence or absence of plasmids encoding NIK, RelB and WT or seven NF-κB2/p100 missense variants either from patients included in this study (*n*=5 variants) or previously reported (*n*=2), or the 14 missense variants reported in public databases at a MAF >10^{-4}, after 24 h (**a**) or 48 h (**b**) of transfection. Data representative of three **(a)** or two **(b)** independent experiment are shown. Bars represent the mean values (± s.d.) from 2 (**a**) or 3 (**b**) independent experiments. (**c**) Western blot of HEK293T cells transfected in the presence or absence of plasmids encoding NIK and NF-κB2/p100 WT or missense variants reported in the public databases at a MAF >10^{-4}. Data representative of one independent experiment are shown.

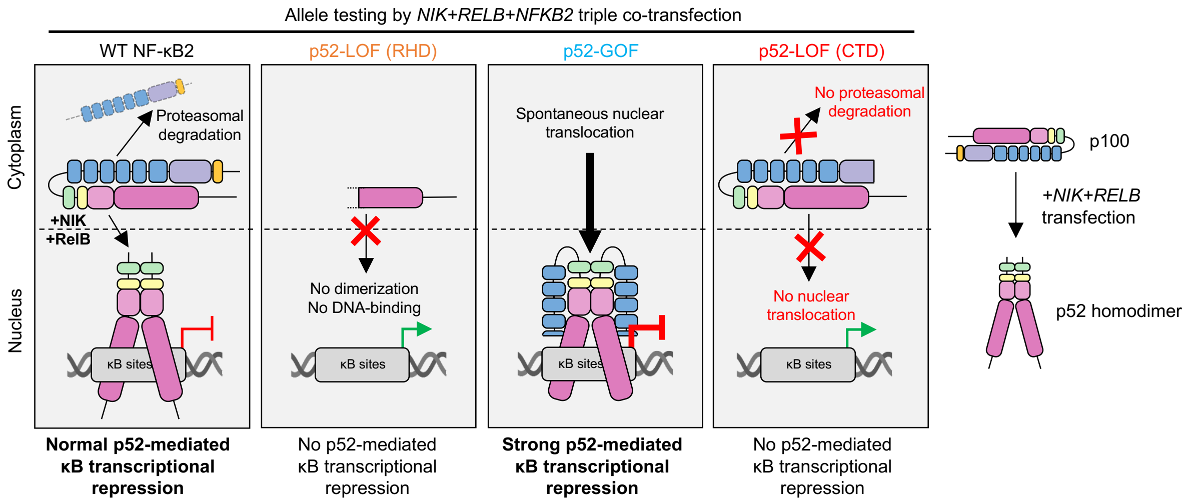

Supplementary Figure 3: Graphical illustration of allele testing in the *NIK-RELB-NFKB2* triple cotransfection assay.

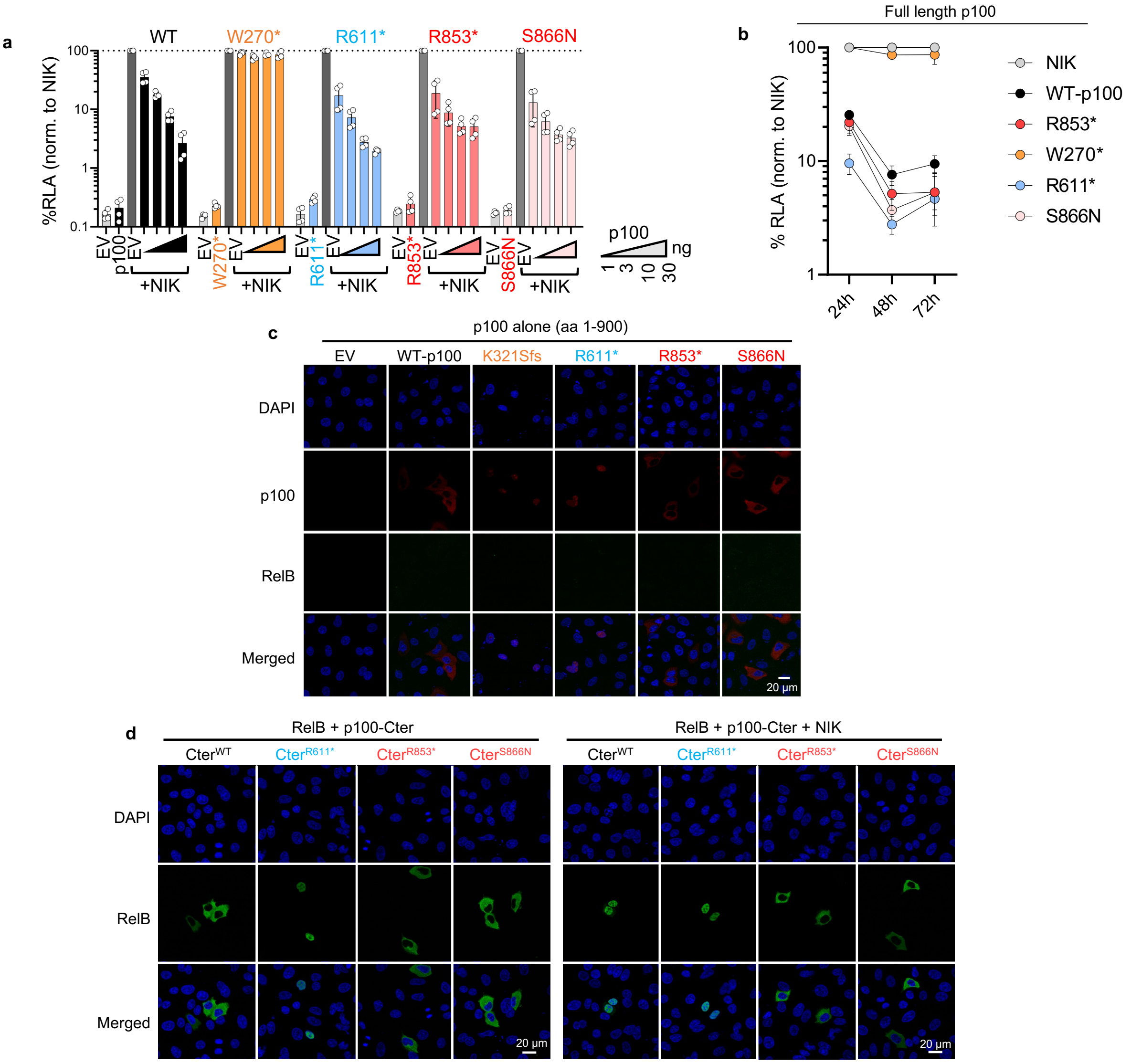

Supplementary Figure 4: The NIK processing-resistant *NFKB2* variants have enhanced p100-IκBδ activity when overexpressed. (**a**) Functional testing of the capacity of the NF-κB2/p100 WT, or W270*, R611*, R853*, S866N variants for p52-dependent κB transcriptional repression of luciferase activity in the presence or absence of plasmids encoding NIK, and various doses of WT or mutant NF-κB2/p100, 48 h after transfection. The results are expressed as a percentage (median ± s.d. of two experiments performed in technical duplicate) of the κB RLA after transfection with NIK alone. **(b)** Kinetic effect of transfection with NIK alone or together with a plasmid encoding WT or variants of NF-κB2/p100 for p52-dependent κB transcriptional repression of luciferase from 24 to 72 h after transfection. Results are expressed as a percentage of the κB RLU after transfection with NIK alone (median ± s.d. of two independent experiments). Data representative of two independent experiment are shown. (**c**) Subcellular localization of NF-κB2/p100 in HeLa cells transfected with NF-κB2/p100 WT or the variants. Data representative of two independent experiment are shown. (**d**) Subcellular localization of RelB after the cotransfection of HeLa cells with plasmids encoding the C-terminal part (Cter, aa 405-900) of the WT ($Cter^{WT}$), LOF-Cter ($Cter^{R611*}$), or GOF-Cter ($Cter^{R853*}$ and $Cter^{S866N}$) p100/NF-κB2 mutants, with or without NIK, 24 h after transfection. Data representative of two independent experiments are shown.

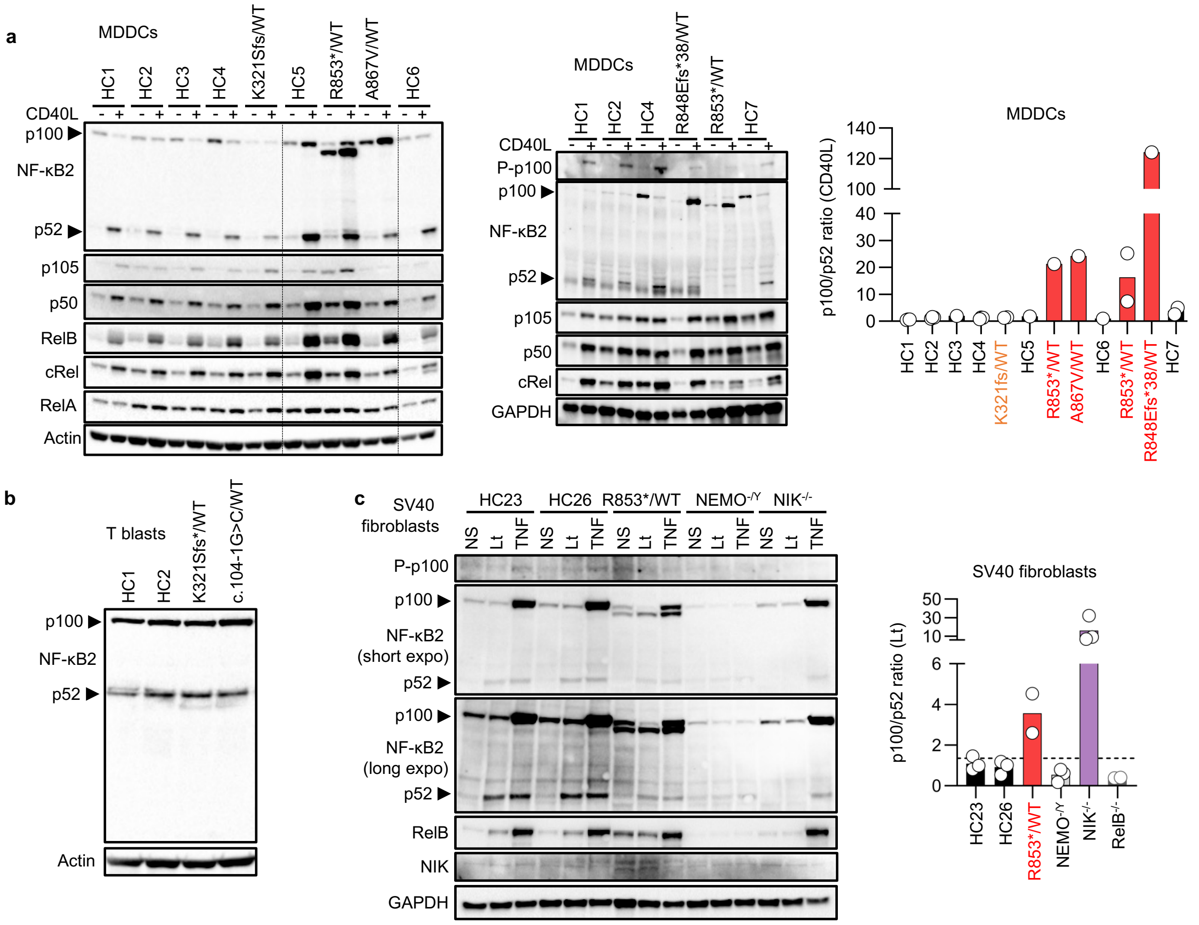

Supplementary Figure 5: The NIK-dependent processing-resistant *NFKB2* variants have enhanced p100-IκBδ activity in heterozygous patients' cells. (a) Western blot of MDDC total cell extracts from controls (HC1-HC7), four patients carrying a $p52^{LOF}/I\kappa B\delta^{GOF}$ (R848Efs*38/WT, *n*=1, R853*/WT, *n*=2, or A867V/WT *n*=1) variant, and a patient with $p52^{LOF}/I\kappa B\delta^{LOF}$ carrying the K321Sfs/WT variant, with and without stimulation with CD40L for 48 h, with a recapitulative graph depicting the total p100/p52 intensity ratio after CD40L stimulation. The bars and error bars represent the mean of two independent experiments and the standard deviation, respectively. The vertical dotted lines on the western blot delimit three independent MDDC differentiation experiments. Data representative of one independent experiment are shown. (**b**) Western blot of T-cell blast total cell extracts from two healthy controls (HC1, HC2), and two patients with the K321Sfs/WT or c.104-1G>C/WT $p52^{LOF}/I\kappa B\delta^{LOF}$ variants. Data representative of one experiment are shown. **(c)** Western blot of P-p100, p100, p52, RelB and NIK in total cell extracts from SV40 fibroblasts from two healthy donors, patients with the $p52^{LOF}/I\kappa B\delta^{GOF}$ R853*/WT variant, complete NEMO deficiency ($NEMO^{-/Y}$) or AR complete NIK deficiency ($NIK^{-/-}$), with or without stimulation with Lt or TNF for 48 h, and a recapitulative graph depicting the total p100/p52 intensity ratio after Lt stimulation. Data representative of at least two independent experiments are shown. Bars represent the mean values (± s.d.) from at least 2 independent experiments.

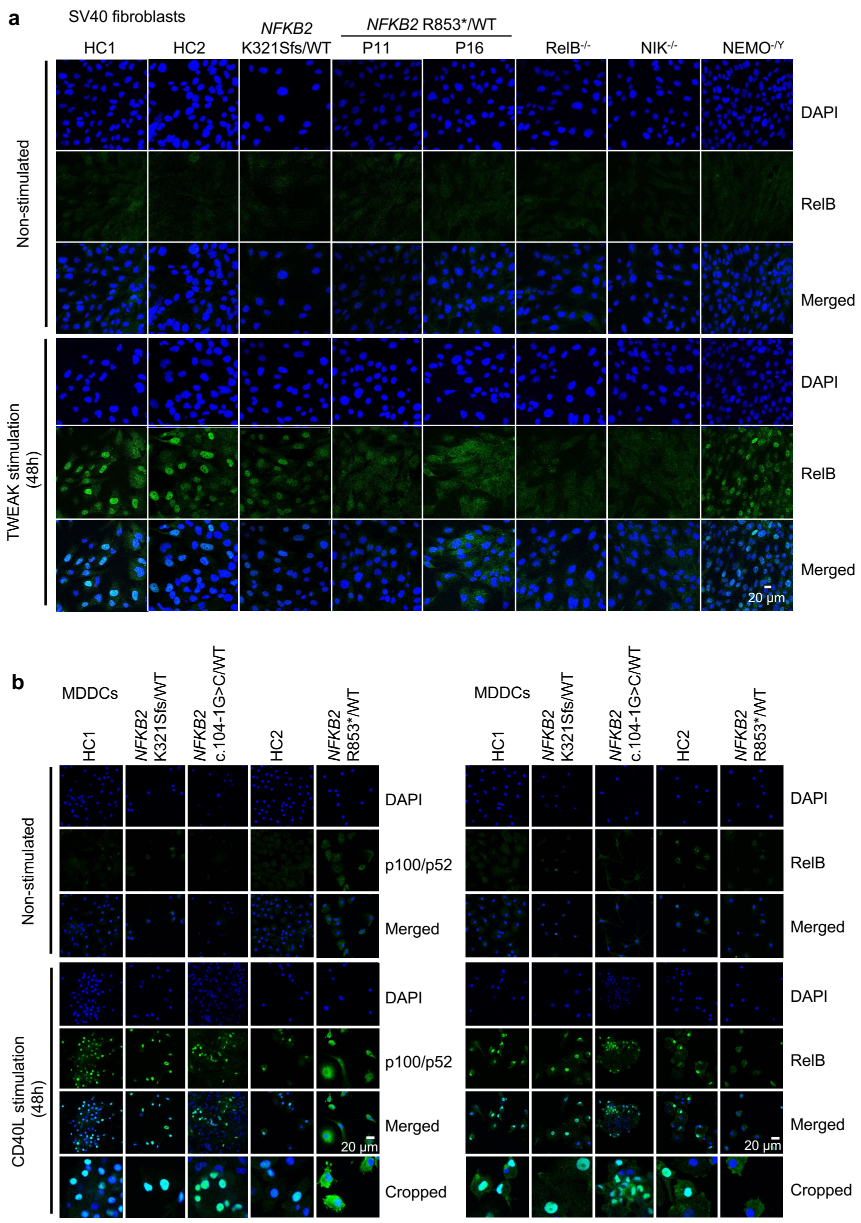

Supplementary Figure 6. The NIK-dependent processing-resistant *NFKB2* variants impair the p52- and/or RelB-dependent dimer nuclear translocation in heterozygous patients' SV40 fibroblasts and MDDCs. (a) Confocal microscopy showing the subcellular distribution of RelB in SV40 fibroblasts from two healthy controls (HC), patients with the $p52^{LOF}/I\kappa B\delta^{LOF}$ K321Sfs*/WT or $p52^{LOF}/I\kappa B\delta^{GOF}$ R853*/WT NF-κB2/p100 variant, AR complete RelB deficiency ($RelB^{-/-}$, Q72Tfs*152/Q72Tfs*152), AR complete NIK deficiency ($NIK^{-/-}$, P565R/P565R), or complete NEMO deficiency ($NEMO^{-/Y}$), with and without stimulation with 100 ng/mL TWEAK for 48 h. Data representative of three independent experiments are shown. (**b**) Confocal microscopy showing the subcellular distribution of p100/p52 (left panel) and RelB (right panel) in MDDCs from two healthy donor, two patients with a $p52^{LOF}/I\kappa B\delta^{LOF}$ (K321Sfs*/WT and c.104-1G>C/WT), and a patient with a $p52^{LOF}/I\kappa B\delta^{GOF}$ R853*/WT NF-κB2/p100 variant, stimulated with CD40L for 48 h. Data representative of one independent experiment are shown.

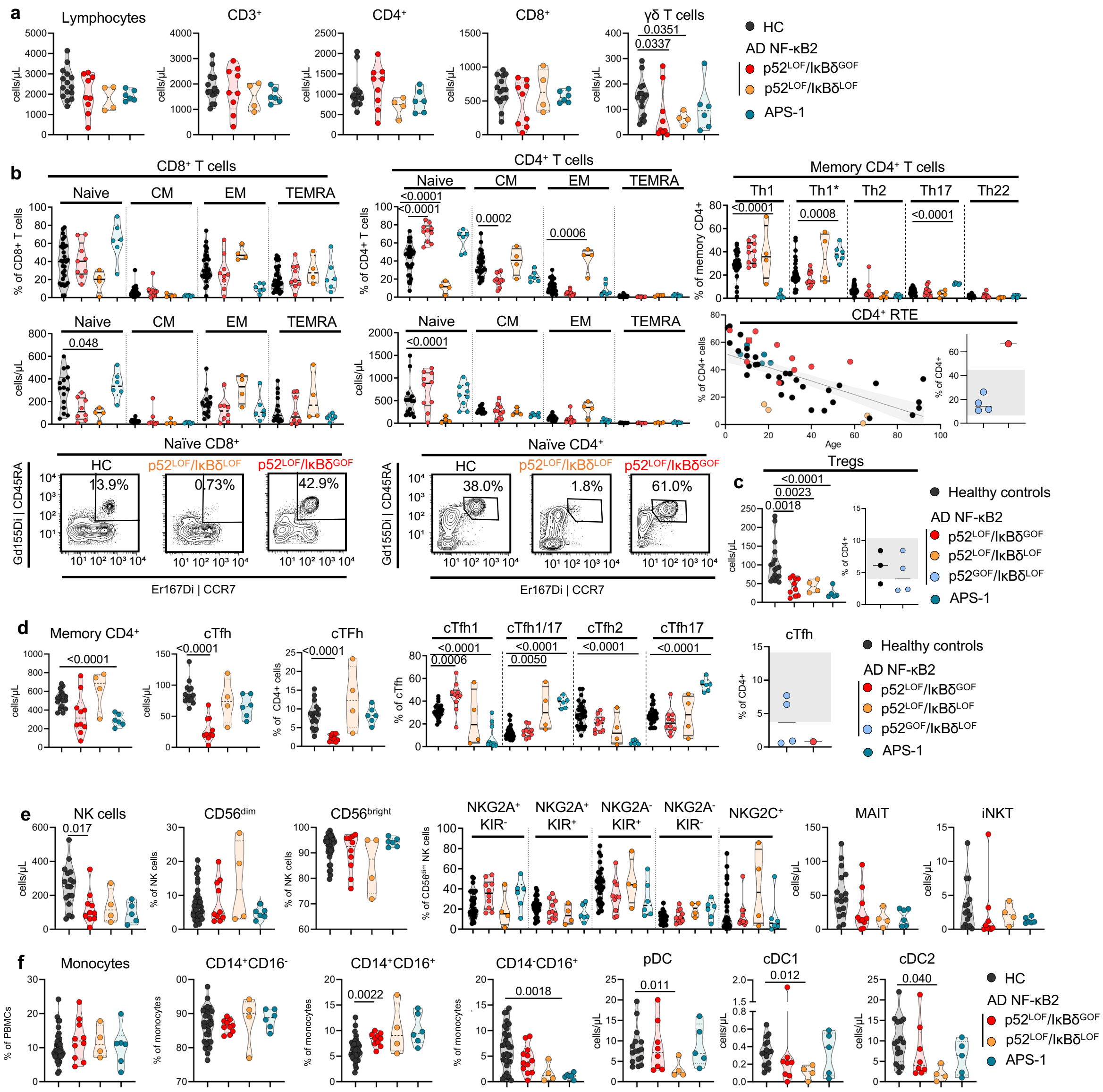

Supplementary Figure 7. Immunological investigation of patients with inborn errors of NF-κB2. (a) Counts for lymphocytes and T-cell subsets; (**b**) proportions and counts for CD8 and CD4 T-cell subsets; (**c**) counts of Tregs; (**d**) counts of memory $CD4^+$ T cells, counts and proportions for cTFh and cTFh subsets; (**e**) counts and proportions for NK cells and NK cell subsets, counts of MAIT and iNKT cells; (**f**) proportions and counts of monocytes and dendritic cells in patients with inborn errors of NF-κB2 and age-matched controls. The $CD4^+$ RTE, Treg and Tfh phenotyping from the $p52^{GOF}/IκBδ^{LOF}$ variant (blue dots) and their age-matched controls (black dots from the same panel) were analyzed from 4 patients with the E418*/WT (*n*=1) or R635*/WT (*n*=3) from[11]. The grey areas in panels **b-d** correspond to the normal distribution of the corresponding cell population. For each panel, the significance of differences was determined using two-tailed Mann-Whitney tests.

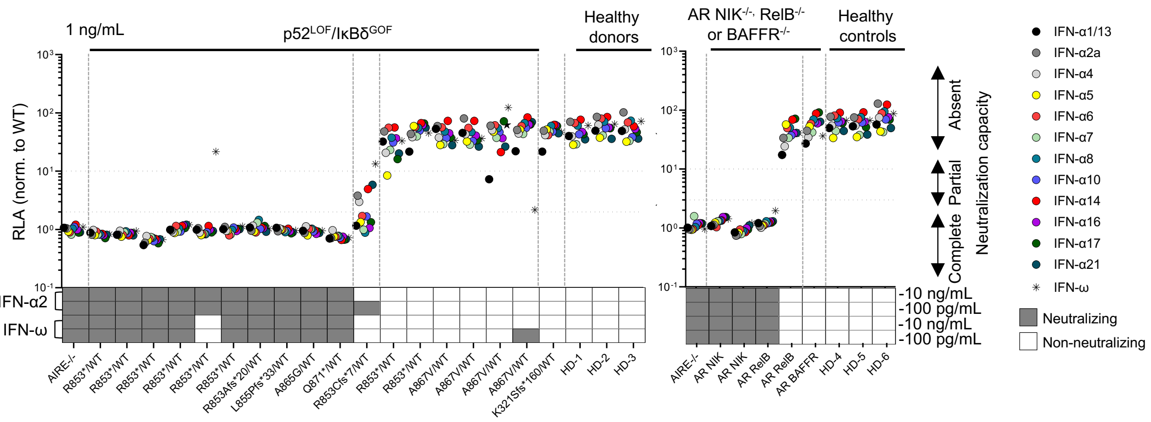

Supplementary Figure 8. Neutralization assay against the 12 IFN-α subtypes and IFN-ω. Luciferase-based neutralization assay for detecting auto-Abs neutralizing the 12 IFN-α subtypes and IFN- ω at a concentration of 1 ng/mL in patients with a p52^{LOF}/IκBδ^{GOF} variant (*n*=17), a p52^{LOF}/IκBδ^{LOF} variant (*n*=1) or APS-1 (*n*=1), and plasma from healthy donors (*n*=3) (left panels) and in patients with inborn errors of RelB (*n*=2), NIK (*n*=2) or BAFFR (*n*=1), and plasma from healthy donors (*n*=3) or patients with APS-1 (*n*=1) (right panel). The neutralization capacity of the corresponding plasma samples against IFN-α2 or IFN-ω at concentrations of 10 ng/mL and 100 pg/mL is indicated by gray (neutralizing) or white (non-neutralizing) squares. Data representative of two independent experiments are shown.

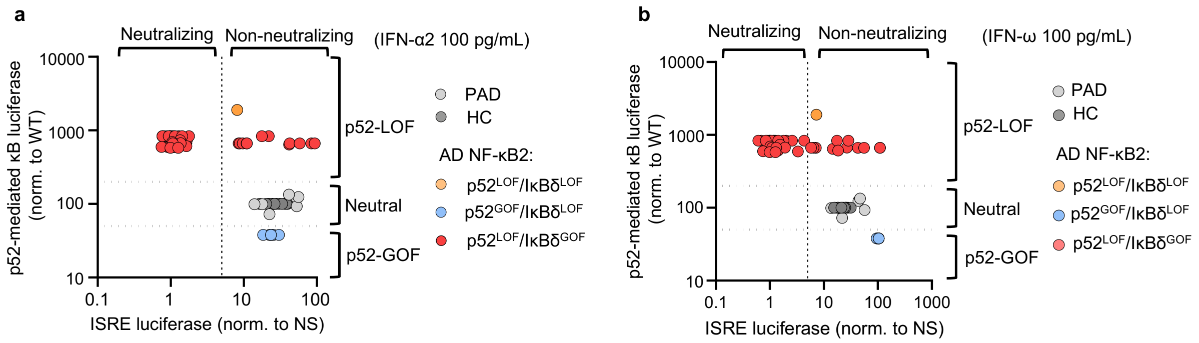

Supplementary Figure 9. Correlation between the p52-dependent transcriptional activity and the neutralizing status of auto-Abs against type I IFNs in patients with inborn errors of NF-κB2. Correlation between the p52-dependent transcriptional activity assessed in the κB luciferase assay (*y*-axis) and the neutralizing status of auto-Abs against IFN-α2 at 100 pg/mL **(a)** or IFN-ω 100 pg/mL **(b)** in the ISRE luciferase assay (*x*-axis) for patients with inborn errors of NF-κB2.

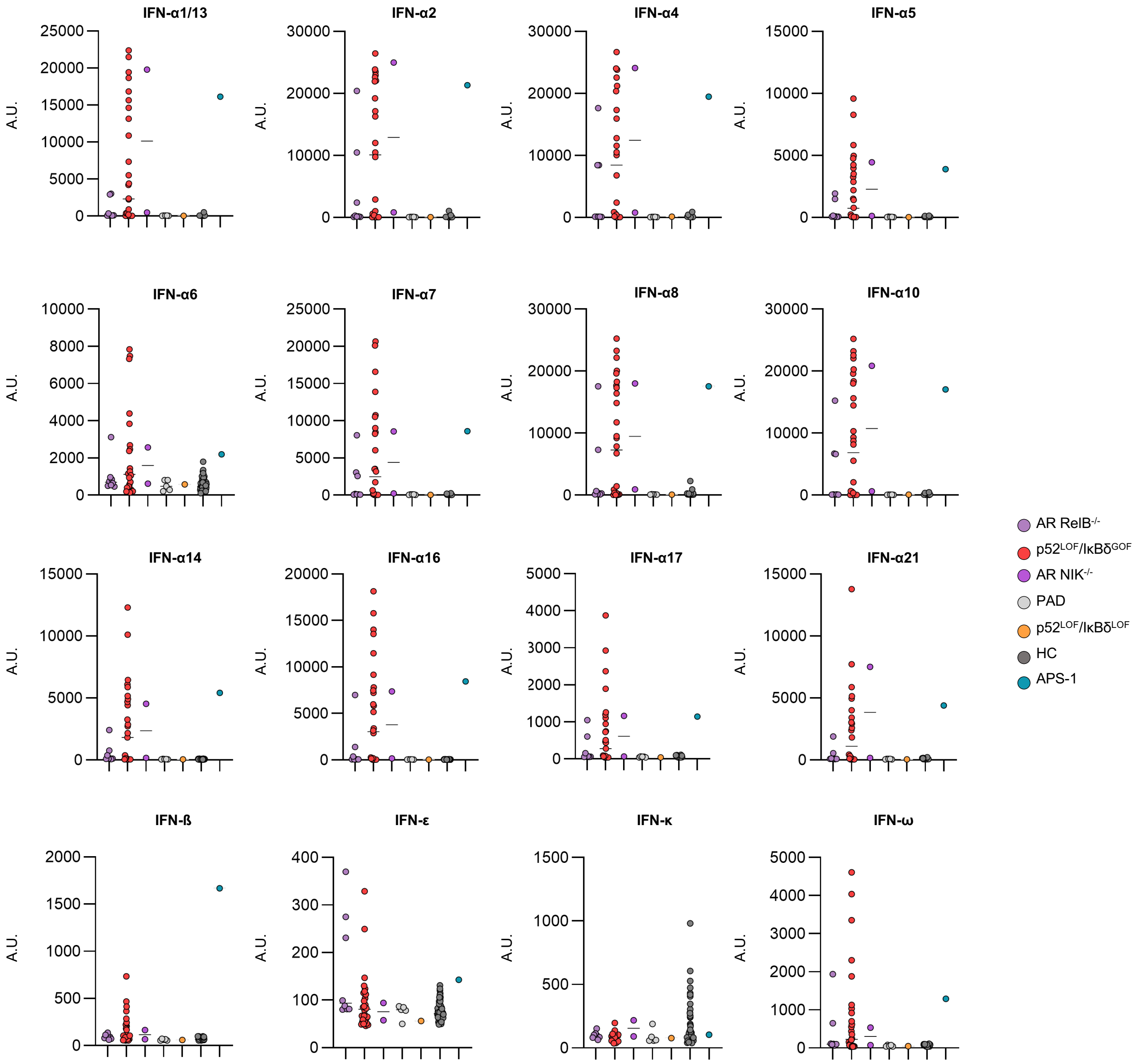

Supplementary Figure 10. Detection of auto-Abs against the 16 type I IFNs in patients in patients with inborn errors of the alternative NF-κB pathway. Detection of auto-Abs against type I IFNs with a multiplex bead array in patients with p52^{LOF}/IκBδ^{GOF} variants (*n*=28 patients), a p52^{LOF}/IκBδ^{LOF} variant (*n*=1 patient), AR RelB (*n*=8 patients) or AR NIK (*n*=2 patients) deficiency, idiopathic PAD (*n*=5 patients), or APS-1 (*n*=1 patient), and healthy controls (HC, *n*=106). Bars represent the median values. Data representative of one independent experiment is shown.

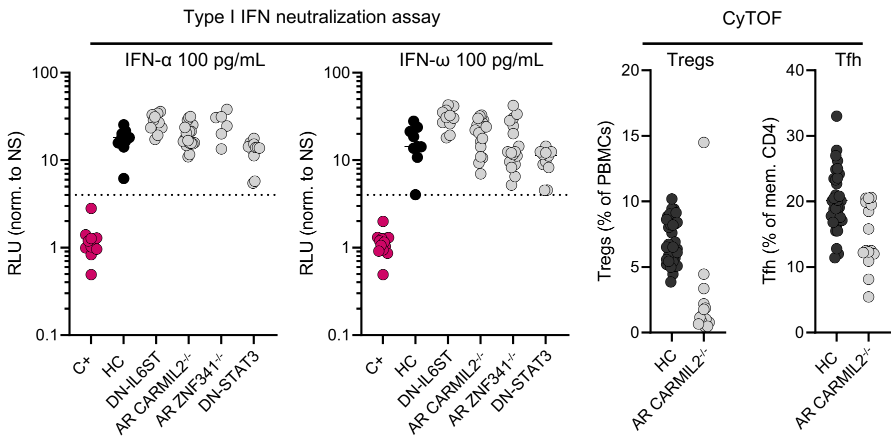

Supplementary Figure 11. Detection of auto-Abs against IFN-α and IFN-ω in patients with inborn errors affecting Tregs and/or Tfh. Luciferase-based neutralization assay for detecting auto-Abs neutralizing 100 pg/mL IFN-α2 or IFN-ω in patients with inborn errors associated with impaired Tfh function (patients with dominant negative (DN) *STAT3* variants, *n*=11), low Treg proportions (dominant negative *IL6ST* variants, *n*=10; AR ZNF341 deficiency, *n*=10), or both low Treg and Tfh counts (AR CARMIL2 deficiency, *n*=16). The proportions of Tregs and Tfh populations, as determined by CyTOF, are shown in the right panel. Data representative of two independent experiments are shown.

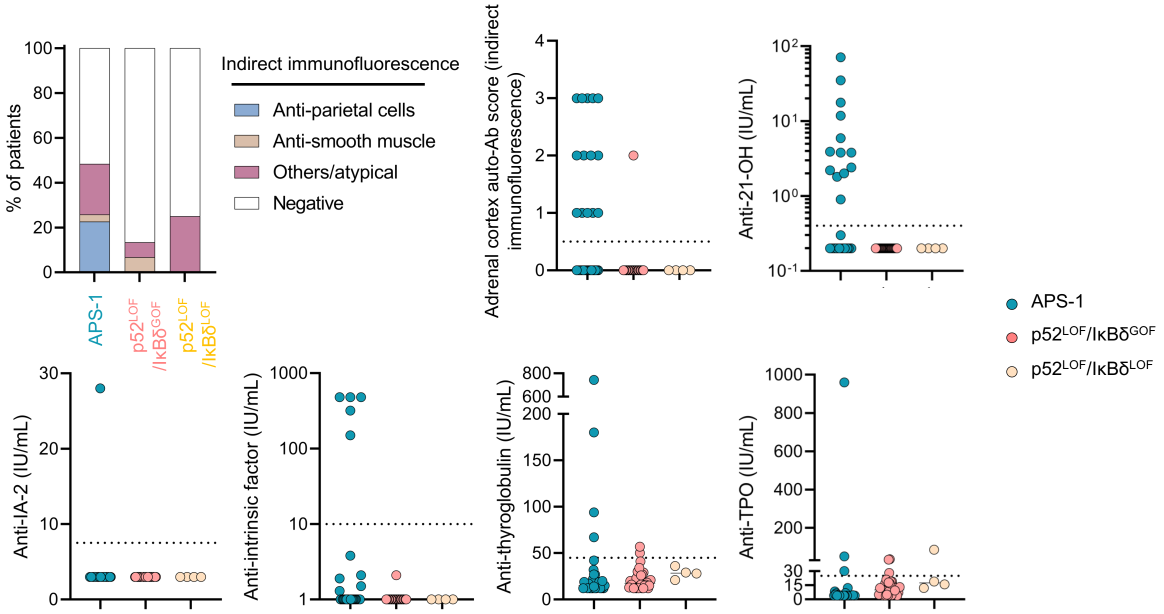

Supplementary Figure 12. Lack of APS-1 tissue-specific auto-Abs in patients with p52^{LOF}/IκBδ^{GOF} variants. Detection of auto-Abs against liver, stomach and muscle by immunofluorescence analyses on triple-tissue samples, and against adrenal glands by immunofluorescence analysis; detection of auto-Abs against 21-hydroxylase (21-OH), IA-2, intrinsic factor, thyroglobulin or thyroperoxidase by ELISA. An atypical auto-Ab indicates an atypical pattern observed under indirect immunofluorescence; Others auto-Abs indicates the detection of a cytoplasmic fluorescence pattern by indirect immunofluorescence on triple-tissue samples other anti-smooth muscles, anti-parietal cells, anti-LKM-1, anti-M2, or anti-LC1. Data representative of one independent experiment is shown.

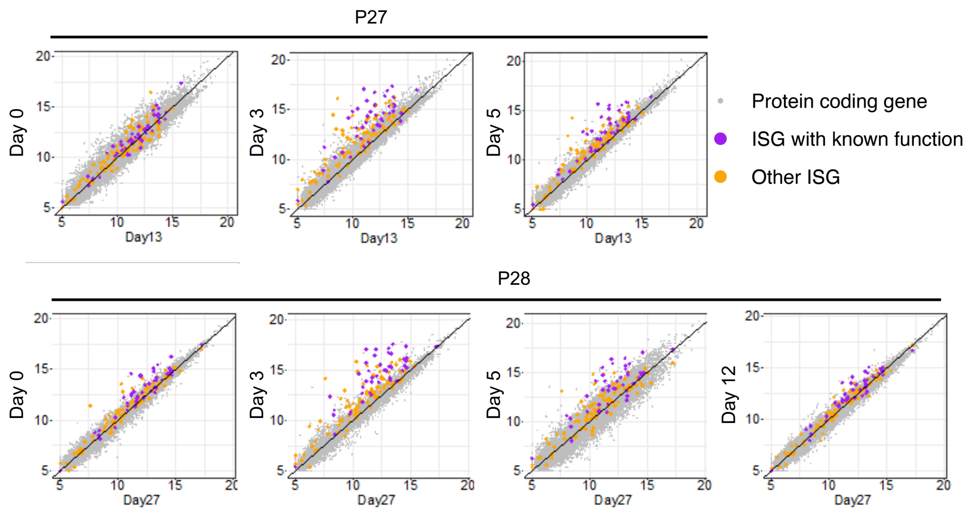

Supplementary Figure 13. IFN module enrichment score during the course of COVID-19. IFN module M.10.1 and M.8.3 enrichment score of individual samples during the course of COVID-19 in P27 and P28 and in two age-matched controls infected with SARS-CoV-2. RNA-seq comparison of gene expression between days 0, 3, 5, 12 and 13 or 27 in P27 and P28.

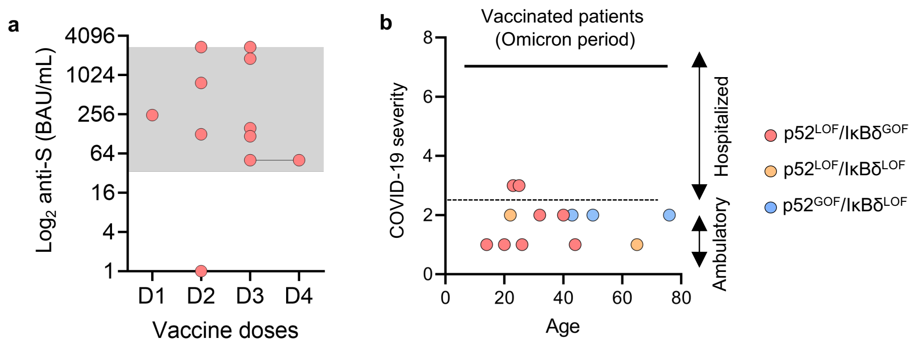

Supplementary Figure 14. Anti-spike IgG response to SARS-CoV-2 vaccination and severity of COVID-19 post-vaccination in patients with p52^{LOF}/IκBδ^{GOF} variants. (**a**) Post-vaccinal anti-S IgG levels in patients with a p52^{LOF}/IκBδ^{GOF} variant who encountered SARS-CoV-2. **(b)** Correlation between COVID-19 severity and age in patients with a p52^{LOF}/IκBδ^{GOF} (*n*=8), p52^{LOF}/IκBδ^{LOF} (*n*=2), or p52^{GOF}/IκBδ^{LOF} (*n*=3) variant who were vaccinated and then infected with SARS-CoV-2 (period from October 2021 to February 2022).

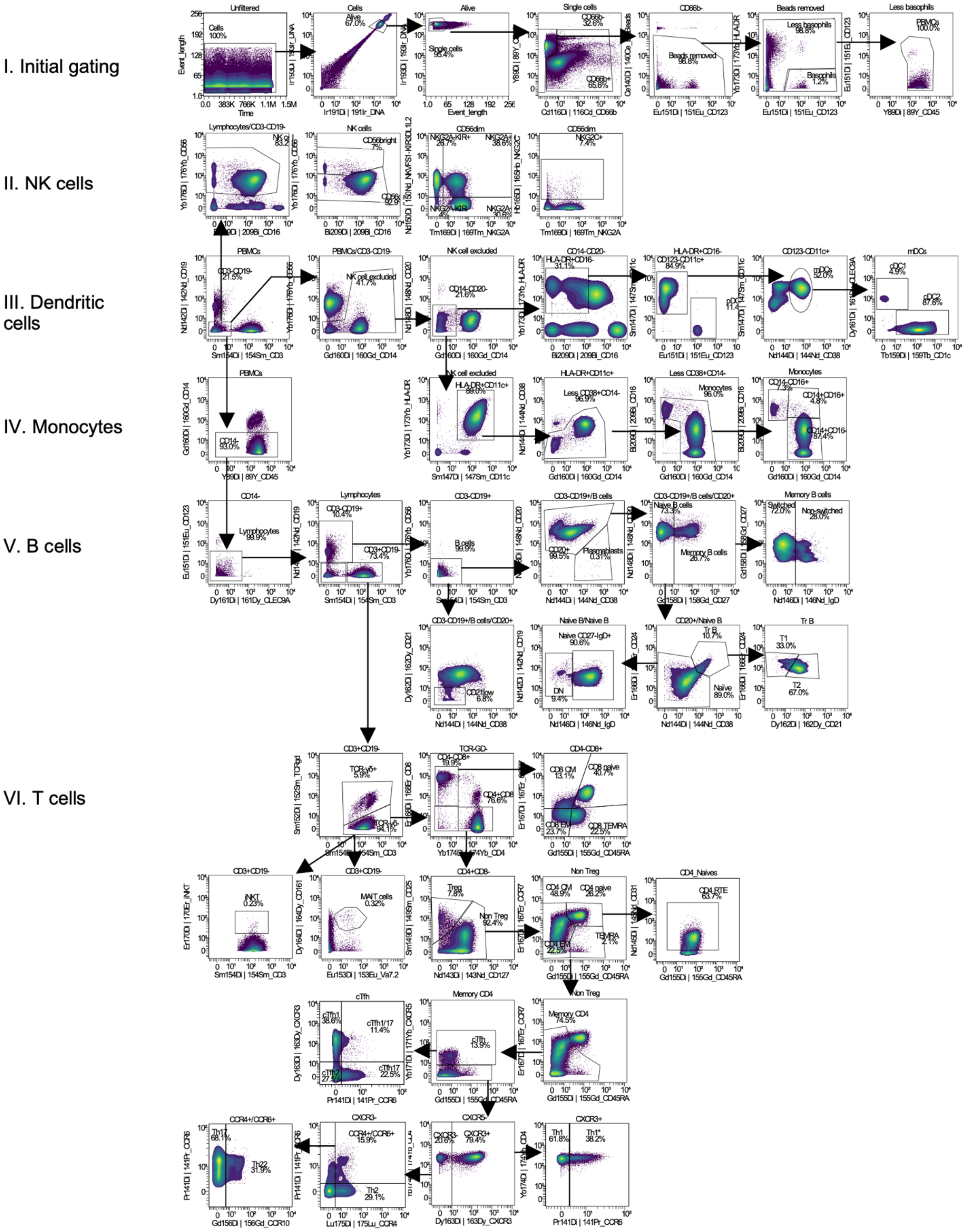

Supplementary Figure 15: Gating strategy for CyTOF immunophenotyping.

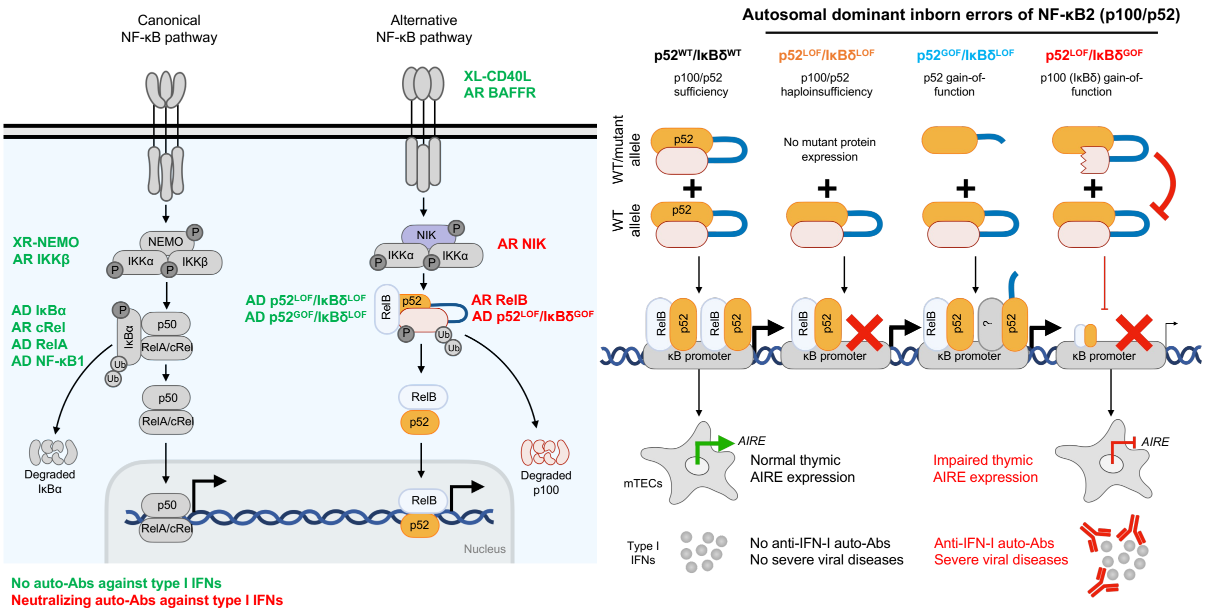

Supplementary Figure 16: Graphical abstract.

Supplementary references

1. Rapaport, F. *et al.* Negative selection on human genes underlying inborn errors depends on disease outcome and both the mode and mechanism of inheritance. *Proc. Natl. Acad. Sci.* **118**, (2021).
2. Brue, T. *et al.* Mutations in NFKB2and potential genetic heterogeneity in patients with DAVID syndrome, having variable endocrine and immune deficiencies. *BMC Med. Genet.* **15**, 139 (2014).
3. Lougaris, V. *et al.* Defective natural killer-cell cytotoxic activity in NFKB2-mutated CVID-like disease. *J. Allergy Clin. Immunol.* **135**, 1641–1643 (2015).
4. Okamura, K. *et al.* Neutrophilic dermatosis associated with an NFKB2 mutation. *Clin. Exp. Dermatol.* **44**, 350–352 (2019).
5. Nogueira, M. *et al.* Symptomatic hypoglycemia in a child with common variable immunodeficiency: Deficient anterior pituitary with variable immune deficiency (DAVID) syndrome. *Clin. Pediatr. Endocrinol.* **29**, 111–113 (2020).
6. Luo, M. Z. *et al.* [De novo NFκB2 gene mutation associated common variable immunodeficiency]. *Zhonghua Er Ke Za Zhi Chin. J. Pediatr.* **56**, 628–632 (2018).
7. Nagai, M., Imai, Y. & Yamanishi, K. Psoriasiform dermatitis associated with common variable immunodeficiency 10 due to an Arg853* mutation in the NFKB2 gene. *J. Dermatol.* **46**, e24–e26 (2019).
8. Ghosh, G. & Wang, V. Y.-F. Origin of the Functional Distinctiveness of NF-κB/p52. *Front. Cell Dev. Biol.* **9**, (2021).
9. Chang, C.-C., Zhang, J., Lombardi, L., Neri, A. & Dalla-Favera, R. Rearranged NFKB-2 Genes in Lymphoid Neoplasms Code for Constitutively Active Nuclear Transactivators. *MOL CELL BIOL* **15**, 8 (1995).
10. Chang, C. C., Zhang, J., Lombardi, L., Neri, A. & Dalla-Favera, R. Mechanism of expression and role in transcriptional control of the proto-oncogene NFKB-2/LYT-10. *Oncogene* **9**, 923–933 (1994).
11. Kuehn, H. S. *et al.* Novel nonsense gain-of-function NFKB2 mutations associated with a combined immunodeficiency phenotype. *Blood* **130**, 1553–1564 (2017).
12. Lee, C. E. *et al.* Autosomal-dominant B-cell deficiency with alopecia due to a mutation in NFKB2 that results in nonprocessable p100. *Blood* **124**, 2964–2972 (2014).
13. Lindsley, A. W. *et al.* Combined Immune Deficiency in a Patient with a Novel NFKB2 Mutation. *J. Clin. Immunol.* **34**, 910–915 (2014).
14. Schumm, K., Rocha, S., Caamano, J. & Perkins, N. D. Regulation of p53 tumour suppressor target gene expression by the p52 NF-κB subunit. *EMBO J.* **25**, 4820–4832 (2006).
15. Betts, J. C. & Nabel, G. J. Differential regulation of NF-kappaB2(p100) processing and control by amino-terminal sequences. *Mol. Cell. Biol.* **16**, 6363–6371 (1996).
16. Fusco, A. J. *et al.* The NF-κB subunit RelB controls p100 processing by competing with the kinases NIK and IKK1 for binding to p100. *Sci. Signal.* **9**, ra96 (2016).
17. Xiao, G., Harhaj, E. W. & Sun, S.-C. NF-κB-Inducing Kinase Regulates the Processing of NF-κB2 p100. *Mol. Cell* **7**, 401–409 (2001).
18. Liang, C., Zhang, M. & Sun, S.-C. β-TrCP binding and processing of NF-κB2/p100 involve its phosphorylation at serines 866 and 870. *Cell. Signal.* **18**, 1309–1317 (2006).
19. Derudder, E. *et al.* Identification and characterization of p100HB, a new mutant form of p100/NF-κB2. *Biochem. Biophys. Res. Commun.* **308**, 744–749 (2003).
20. Qing, G., Qu, Z. & Xiao, G. Regulation of NF-κB2 p100 Processing by Its cis-Acting Domain *. *J. Biol. Chem.* **280**, 18–27 (2005).
21. Klemann, C. *et al.* Clinical and Immunological Phenotype of Patients With Primary

Immunodeficiency Due to Damaging Mutations in NFKB2. *Front. Immunol.* **10**, (2019).
22. Qing, G., Qu, Z. & Xiao, G. Endoproteolytic processing of C-terminally truncated NF-κB2 precursors at κB-containing promoters. *Proc. Natl. Acad. Sci.* **104**, 5324–5329 (2007).
23. Solan, N. J., Miyoshi, H., Carmona, E. M., Bren, G. D. & Paya, C. V. RelB Cellular Regulation and Transcriptional Activity Are Regulated by p100*. *J. Biol. Chem.* **277**, 1405–1418 (2002).
24. Uhlén, M. *et al.* Tissue-based map of the human proteome. *Science* **347**, 1260419 (2015).
25. Sun, S.-C. The non-canonical NF-κB pathway in immunity and inflammation. *Nat. Rev. Immunol.* **17**, 545–558 (2017).
26. Saitoh, T. *et al.* TWEAK Induces NF-κB2 p100 Processing and Long Lasting NF-κB Activation*. *J. Biol. Chem.* **278**, 36005–36012 (2003).
27. Willmann, K. L. *et al.* Biallelic loss-of-function mutation in NIK causes a primary immunodeficiency with multifaceted aberrant lymphoid immunity. *Nat. Commun.* **5**, 5360 (2014).
28. Sharfe, N. *et al.* The effects of RelB deficiency on lymphocyte development and function. *J. Autoimmun.* **65**, 90–100 (2015).
29. Piscianz, E. *et al.* Familial hypogammaglobulinemia with high RTE and naïve T lymphocytes. *Inflamm. Res.* **68**, 901–904 (2019).
30. van der Wijst, M. G. P. *et al.* Type I interferon autoantibodies are associated with systemic immune alterations in patients with COVID-19. *Sci. Transl. Med.* **13**, eabh2624 (2021).
31. Bastard, P. *et al.* Autoantibodies neutralizing type I IFNs are present in ~4% of uninfected individuals over 70 years old and account for ~20% of COVID-19 deaths. *Sci. Immunol.* (2021) doi:10.1126/sciimmunol.abl4340.
32. Lopez, J. *et al.* Early nasal type I IFN immunity against SARS-CoV-2 is compromised in patients with autoantibodies against type I IFNs. *J. Exp. Med.* **218**, e20211211 (2021).
