# Supplementary material for: Autoantibodies against type I IFNs in humans with alternative NF-κB pathway deficiency
Source: Nature. 2023 Nov 8;623(7988):803–13. doi: 10.1038/s41586-023-06717-x (PMC10665196; doi:10.1038/s41586-023-06717-x)
Supplement: Supplementary file 2 — Reporting Summary [file 41586_2023_6717_MOESM2_ESM.pdf]

nature portfolio

Corresponding author(s): Jean-Laurent Casanova, Ann Puel, Tom Le Voyer

Last updated by author(s): Sept, 04, 2023

Reporting Summary

Nature Portfolio wishes to improve the reproducibility of the work that we publish. This form provides structure for consistency and transparency in reporting. For further information on Nature Portfolio policies, see our Editorial Policies and the Editorial Policy Checklist.

Statistics

For all statistical analyses, confirm that the following items are present in the figure legend, table legend, main text, or Methods section.

n/a	Confirmed	
☐	☒	The exact sample size (*n*) for each experimental group/condition, given as a discrete number and unit of measurement
☐	☒	A statement on whether measurements were taken from distinct samples or whether the same sample was measured repeatedly
☐	☒	The statistical test(s) used AND whether they are one- or two-sided<br>*Only common tests should be described solely by name; describe more complex techniques in the Methods section.*
☒	☐	A description of all covariates tested
☒	☐	A description of any assumptions or corrections, such as tests of normality and adjustment for multiple comparisons
☐	☒	A full description of the statistical parameters including central tendency (e.g. means) or other basic estimates (e.g. regression coefficient) AND variation (e.g. standard deviation) or associated estimates of uncertainty (e.g. confidence intervals)
☐	☒	For null hypothesis testing, the test statistic (e.g. *F*, *t*, *r*) with confidence intervals, effect sizes, degrees of freedom and *P* value noted<br>*Give P values as exact values whenever suitable.*
☒	☐	For Bayesian analysis, information on the choice of priors and Markov chain Monte Carlo settings
☒	☐	For hierarchical and complex designs, identification of the appropriate level for tests and full reporting of outcomes
☒	☐	Estimates of effect sizes (e.g. Cohen's *d*, Pearson's *r*), indicating how they were calculated

Our web collection on statistics for biologists contains articles on many of the points above.

Software and code

Policy information about availability of computer code

Data collection	Patient data were collected using Microsoft Excel.
Data analysis	Statistical softwares:<br>GraphPad Prism, version 9.5.0<br><br>Huprot microArray :<br>GenePix Pro 5.1.0.19 or GenePix Pro 7<br>Mapix 9.1.0.<br><br>Cytometry by Time of Flight :<br>Omiq: https://app.omiq.ai<br><br>RNA-seq:<br>STAR (2.6.1d): https://github.com/alexdobin/STAR<br>DEseq2 (1.40.2): https://bioconductor.org/packages/release/bioc/html/DESeq2.html<br>GSVA package (1.48.3): https://bioconductor.org/packages/release/bioc/html/GSVA.html<br>BloodGen3Module gene set (1.8.0): http://bioconductor.org/packages/release/bioc/html/BloodGen3Module.html

For manuscripts utilizing custom algorithms or software that are central to the research but not yet described in published literature, software must be made available to editors and reviewers. We strongly encourage code deposition in a community repository (e.g. GitHub). See the Nature Portfolio guidelines for submitting code & software for further information.

Data

Policy information about availability of data

All manuscripts must include a data availability statement. This statement should provide the following information, where applicable:

- Accession codes, unique identifiers, or web links for publicly available datasets
- A description of any restrictions on data availability
- For clinical datasets or third party data, please ensure that the statement adheres to our policy

All the data supporting the findings of this study are available within the article and its supplementary information.
The RNA-seq data generated in this study have been deposited in the NCBI database under NCBI-SRA project PRJNA989123 (https://www.ncbi.nlm.nih.gov/bioproject/PRJNA989123).
All the other data are presented in the main or in the supplementary materials (uncropped wester blots and source data from mice experimnets). All the other data supporting the findings of this study are available from the corresponding authors upon reasonable request.

Research involving human participants, their data, or biological material

Policy information about studies with human participants or human data. See also policy information about sex, gender (identity/presentation), and sexual orientation and race, ethnicity and racism.

Reporting on sex and gender	Information about sex was collected using medical questionnaire in the 87 patients with inborn errors of the alternative NF-kB pathway. No gender analysis was performed.
Reporting on race, ethnicity, or other socially relevant groupings	Geographic origin
Population characteristics	age, medical and genetic diagnosis, treatments
Recruitment	All patients with a genetic diagnosis of an inborn error of the alternative NF-κB pathway were included without any restriction or bias.
Ethics oversight	Patients were included in the C18-41 Genetic Predisposition to Severe Infections study approved by the Sud Est II ethics committee (approval no. 2022-A00257-36) in France. All the enrolled subjects provided written informed consent and were collected through protocols conforming to local ethics requirements. Ethics approval was obtained from the Comitato Etico Provinciale (NP 4000 – Studio CORONAlab) in Brescia, Italy, the French Ethics Committee "Comité de Protection des Personnes," Ile de France II" (2010-A00634-35- protocol no. C10-13), and the Rockefeller University Institutional Review Board in New York (protocol no. JCA-0700).

Note that full information on the approval of the study protocol must also be provided in the manuscript.

Field-specific reporting

Please select the one below that is the best fit for your research. If you are not sure, read the appropriate sections before making your selection.

☒ Life sciences ☐ Behavioural & social sciences ☐ Ecological, evolutionary & environmental sciences

For a reference copy of the document with all sections, see nature.com/documents/nr-reporting-summary-flat.pdf

Life sciences study design

All studies must disclose on these points even when the disclosure is negative.

Sample size	We conducted an international cohort study of patients with a known genetic diagnosis of an inborn error of the alternative NF-κB pathway. Each individual for whom a clinical questionnaire and a plasma or serum sample were available was included without restriction. This study encompasses 73 cases, whether symptomatic or not, all carrying a rare heterozygous NFKB2 variants (minor allele frequency <10-4), either previously reported or newly identified. Additionally, we included ten more patients with inborn errors of the non-canonical NF-κB signaling pathway (2 with NIK deficiency and 8 with RelB deficiency). In each of the experiments performed, we included a comparable or higher number of healthy controls.
Data exclusions	No data were excluded
Replication	The detection of plasma anti-IFN-a, anti-IFN-w, anti-IFN-b IgG autoantibodies, the assessment of their neutralization activity, and their screening by HuProt microarray were performed in at least two independent experiments. All the tests of the alleles (by luciferase, by western blot, by immunostaining) were performed in at least three independent experiments. On biological samples, western blot and immunostaining experiments were performed in two independent experiments. All attempts at replication were sucessful. CyTOF, IFN score, RNAseq, bead arrays, and human thymic biopsy immunostaining were performed only once due to the unique nature of these biological

samples, but all these experiments were performed in two or more individiuals of the same genotype exept for the thymic staining experiment which was performed in two individuals with inborn errors of the same siganling pathway.

Randomization

No randomization was applicable to this study because this study investigated rare patients with inborn errors of immunity. The patients' clinical and immunological phenotypes were compared to those of heathy controls or to those of patients with other inborn errors of the canonical or alternative NF-kB pathway and APS-1 patients.

Blinding

Investigators performing auto-antibody screening by HuProt, indirect immunofluorescence, ELISA, bead-array, RNA-seq, IFN scores, CyTOF were blinded to the characteristics of the patients. For other experiments, quantifications were performed equally to all samples and replicates in an objective manner.

Reporting for specific materials, systems and methods

We require information from authors about some types of materials, experimental systems and methods used in many studies. Here, indicate whether each material, system or method listed is relevant to your study. If you are not sure if a list item applies to your research, read the appropriate section before selecting a response.

Materials & experimental systems

n/a	Involved in the study
☐	☒ Antibodies
☐	☒ Eukaryotic cell lines
☒	☐ Palaeontology and archaeology
☐	☒ Animals and other organisms
☒	☐ Clinical data
☒	☐ Dual use research of concern
☒	☐ Plants

Methods

n/a	Involved in the study
☒	☐ ChIP-seq
☐	☒ Flow cytometry
☒	☐ MRI-based neuroimaging

Antibodies

Antibodies used

Human antigens:

Immunofluorescent staining on thymic sections
KRT8-Alexa647, Rb (clone EP1628Y) - Abcam ab192468, 1:300;
KRT5 Alexa488, Rb (clone EP1601Y) - Abcam ab193894, 1:300;
AIRE, rat – eBioscience 14-9534-82, 1:50
pan-ketatin, Rb -Abcam ab9377, 1 :200.
K10 Alexa647, Rb (clone EP1607IHCY)
Abcam ab194231, 1:300
UEA-1 biotinylated – Vector Laboratories B-1065-2, 1:500

Western blot and immunofluoresence
100/p52 (4882; Cell Signaling Technology) 1/1000
p105/p50 (N terminus; 3035; Cell Signaling Technology) 1/1000
p65 (sc-372; Santa Cruz Biotechnology), 1/1000
RelB (sc-48366; Santa Cruz Biotechnology), 1/800
c-Rel (sc-6955; Santa Cruz Biotechnology), 1/1000
Amersham ECL mouse IgG, HRP-linked whole antibody (from sheep; NA931; GE Healthcare Life Sciences)
Amersham ECL rabbit IgG, HRP-linked whole antibody (from donkey; NA934; GE Healthcare Life Sciences)
goat anti-mouse IgG Alexa Fluor 488 (#A-11029, dilution 1/250)
goat anti-rabbit IgG Alexa Fluor 633 (#A-11037, dilution 1/250)
Alexa Fluor 647 goat anti-human IgG (Thermo Fisher Scientific, ref. number A21445

IFNA alpha (=IFNA2) PBL Assay Science PBL11101-2
IFNA1 MedChemExpress HY-P70241
IFNA7 Novus 11079-IF
IFNA10 Origene TP314055
IFNA14 Prospec cyt-135-b
IFNA16 Novus 11190-1 Lot# 6865
IFNA17 Origene TP320824
IFNA2 Origene TP321091
IFNA21 Origene TP310115
IFNA4 Origene TP323649
IFNA5 Origene TP310825
IFNA6 Origene TP760329
IFNA8 Origene TP311169
IFNB1 MedChemExpress HY-P73128
IFNE R&D 9667-ME/CF
IFNG MedChemExpress HY-P7025
IFNG Origene TP721239

IFNK Cusabio CSB-EP889172HU
IFNL4 R&D 9165-IF
IFNW1 MedChemExpress HY-P7201
IL17A Origene TP318057
IL17F MedChemExpress HY-P70540
IL22 Origene TP309995
IL28a Nordic biosite (Sino Biological) 12340-H0By
IL28b Abcam ab276441
IL29 Abcam ab155625
IL6 MedChemExpress HY-P7044G
RBM38 Origene TP311451
ATP4A Origene LY424563
TROVE2 = Ro60 OriGene TP306071

Mouse antigens:
AIRE (5H12, eBioscience Cat#53593482),
CD45 (30-F11, Biolegend Cat#103130)
EpCAM (G8.8, Biolegend Cat# 118218)
I-Ak (10-3.6, Biolegend Cat#109908)
K5 (EP1601Y, Abcam Cat#193895),
K10 (EP1607IHCY, Abcam Cat#194231).

Validation	The specificity of primary antibodies for immunofluorescent staining was verified on human thymic sections using various dilutions (including the manufacturer's recommended dilution).<br><br>Primary human antibodies were internally tested for Western blotting, flow cytometry, or microscopy through preliminary experiments using either cell lines or primary cells from control subjects. This was done to ensure their functionality before performing experiments with patient cells. All mice primary antibodies were validated using primary mouse or tissues or single cell suspensions.

Eukaryotic cell lines

Policy information about cell lines and Sex and Gender in Research

Cell line source(s)	HEK293T cells; HeLa cells from ATCC
Authentication	Nonce of the cell lines used were authenticated
Mycoplasma contamination	Cells were regularly screened for the presence of mycoplasma and used in the absence of mycoplasma contamination
Commonly misidentified lines (See ICLAC register)	none

Animals and other research organisms

Policy information about studies involving animals; ARRIVE guidelines recommended for reporting animal research, and Sex and Gender in Research

Laboratory animals	Mice were on the NOD background (Nfkb2-Y868*/WT, Aire-KO, WT) or B6 (Rag2-KO: B6.Cg-Rag2tm1.1Cgn/J. Both male and female mice were used. All NOD mice were 8-12 weeks of age, matched for age and sex. All B6 Rag2-KO were 10-15 weeks of age. Mice in standard 12:12 light:dark cycle; humidity kept between 30-70%; temperature 68-79 degrees F.
Wild animals	No wild animals were used in this study
Reporting on sex	15 female and 10 male Nfkb2-Y868*/WT were used. 5 female and 7 male NOD Aire-KO were used. 7 female and 5 male NOD Aire WT were used. 4 female and 1 male Rag2-KO were used.
Field-collected samples	No field collected samples were used in the study.
Ethics oversight	Mice were maintained in accordance with the guidelines established by the Institutional Committee on Animal Use and Care (IACUC) and Laboratory Animal Resource Center (LARC). Animal procedures were approved by the IACUC and LARC at UCSF

Note that full information on the approval of the study protocol must also be provided in the manuscript.

Flow Cytometry

Plots

Confirm that:

☒ The axis labels state the marker and fluorochrome used (e.g. CD4-FITC).

☒ The axis scales are clearly visible. Include numbers along axes only for bottom left plot of group (a 'group' is an analysis of identical markers).

☒ All plots are contour plots with outliers or pseudocolor plots.

☒ A numerical value for number of cells or percentage (with statistics) is provided.

Methodology

Sample preparation	Single-cell suspensions were incubated with Live/Dead Fixable Blue Dead Cell Stain (Thermo Fisher Scientific) in 1X PBS for 15 min at 4°C and then washed in PBS. They were blocked by incubation with anti-mouse CD16/CD32 (24G2) antibody (UCSF Hybridoma Core Facility) for 15 min at 4°C before cell surface marker staining in FACS buffer for 30 min at 4°C. Cells were fixed and permeabilized with the FoxP3 staining buffer kit (eBioscience), according to the manufacturer’s protocol for intracellular protein staining.
Instrument	LSRII Flow Cytometer (BD Biosciences)
Software	FlowJo 10.8.1 software
Cell population abundance	All cells were sorted to a purity of > 95%
Gating strategy	All cells were gated on singlets, live cells, and FSC/SSC.<br><br>mTECs: CD11c- CD45- EPCAM+ (+/- Ly51-)

☒ Tick this box to confirm that a figure exemplifying the gating strategy is provided in the Supplementary Information.
