# Supplementary material for: Autoantibodies against type I IFNs in humans with alternative NF-κB pathway deficiency
Source: Nature. 2023 Nov 8;623(7988):803–13. doi: 10.1038/s41586-023-06717-x (PMC10665196; doi:10.1038/s41586-023-06717-x)
Supplement: Supplementary file 3 — Uncropped images from the western blots displayed in the indicated figures. [file 41586_2023_6717_MOESM3_ESM.pdf]

Supplementary figure 1 (1)

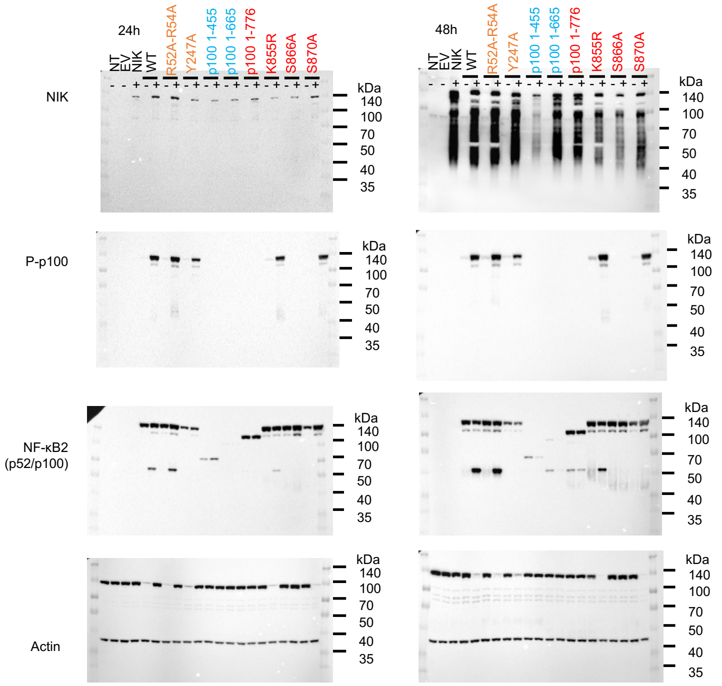

Extended Data Figure 3

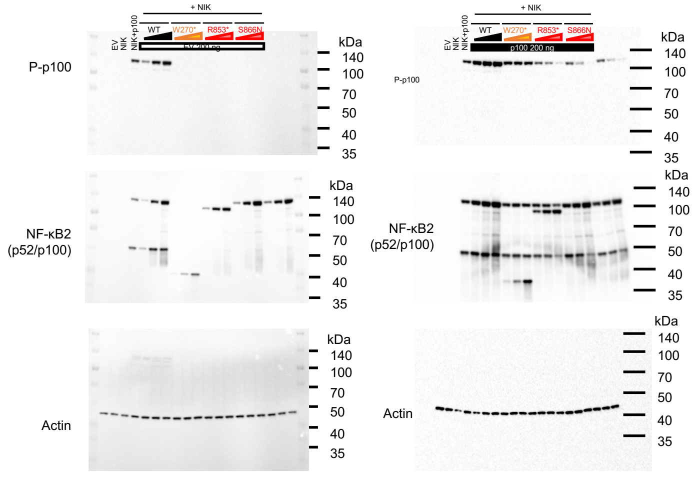

Extended Data Figure 4d

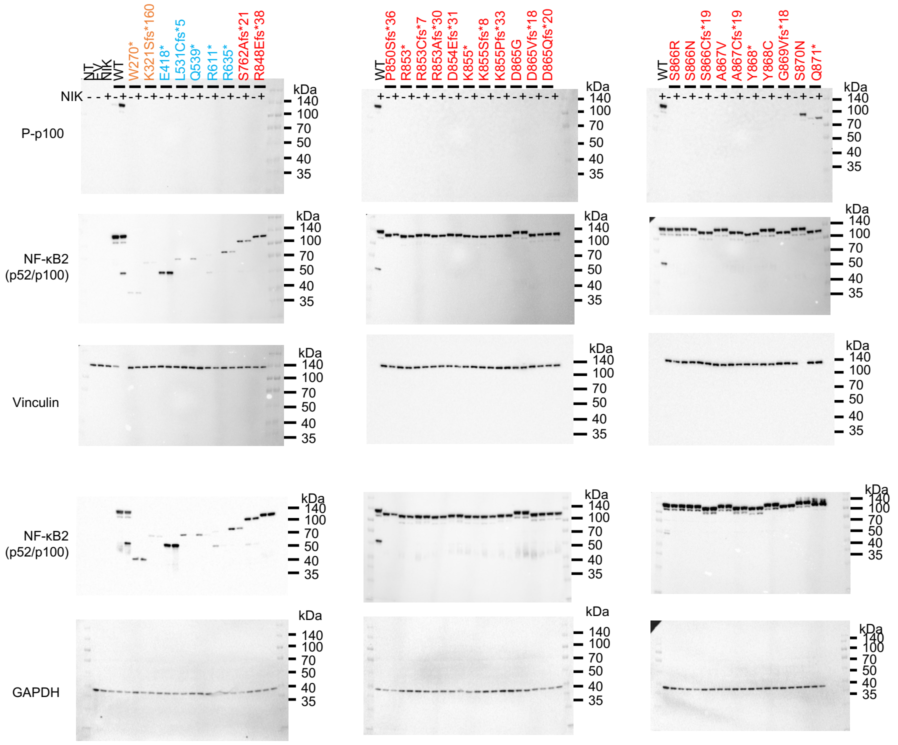

Extended Data Figure 4a

Supplementary figure 1 (2)

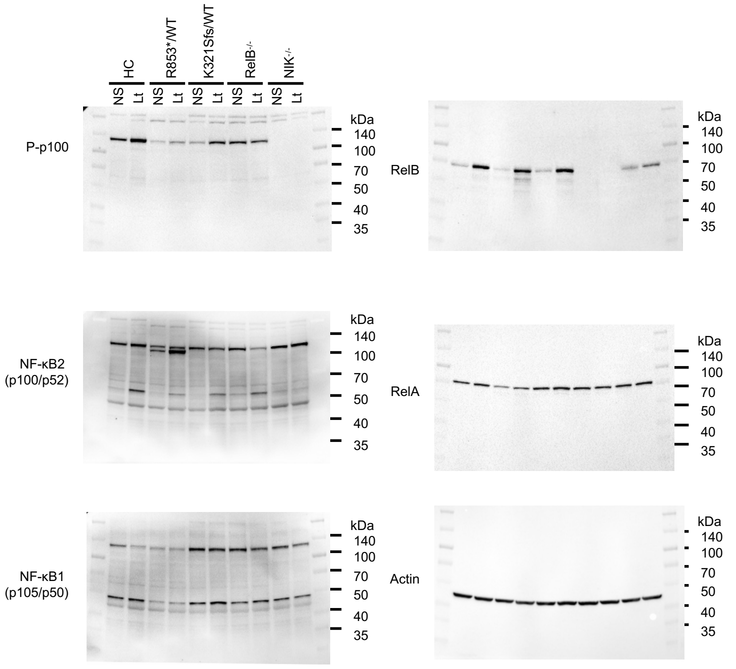

Extended Data Figure 5a

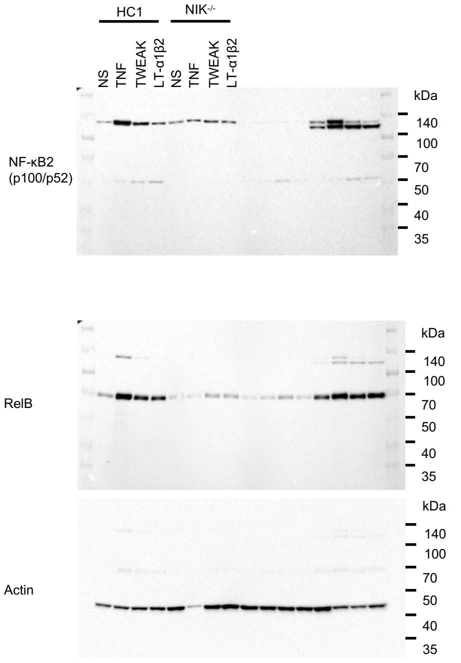

Extended Data Figure 5b

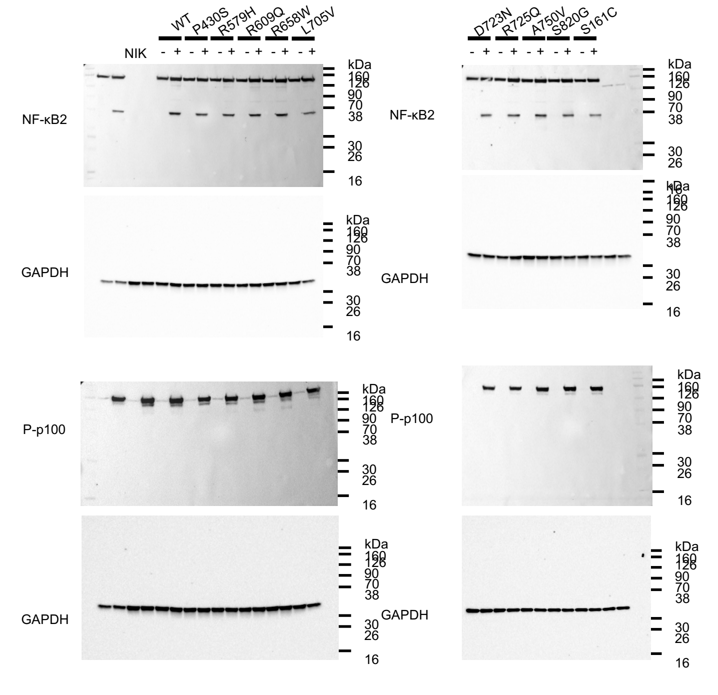

Supplementary Figure 2

Supplementary figure 1 (3)

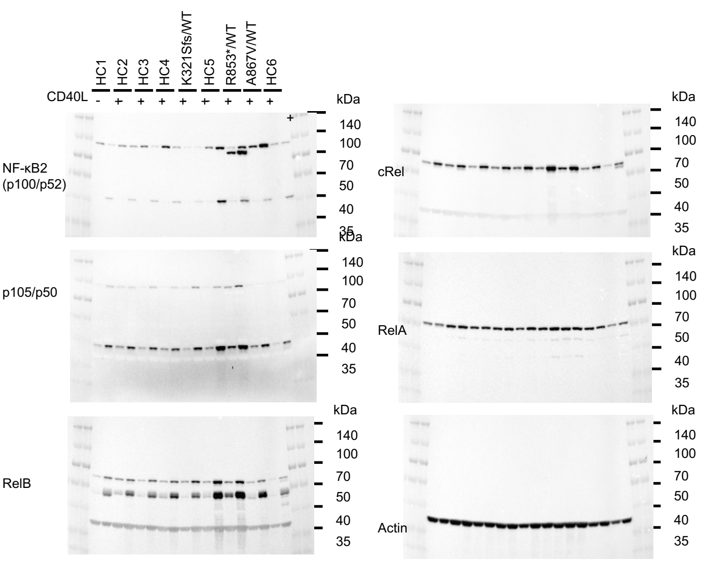

Supplementary Figure 5a (1)

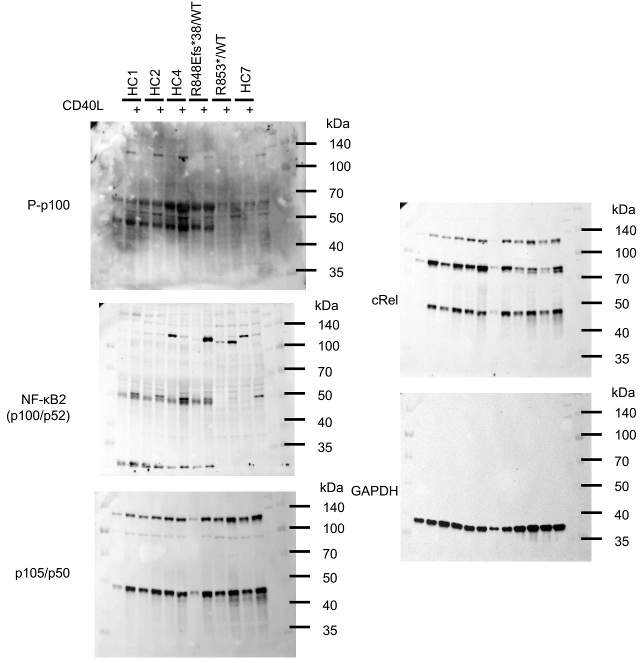

Supplementary Figure 5a (2)

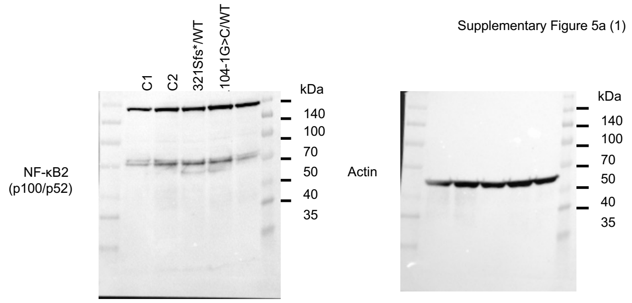

Supplementary Figure 5b

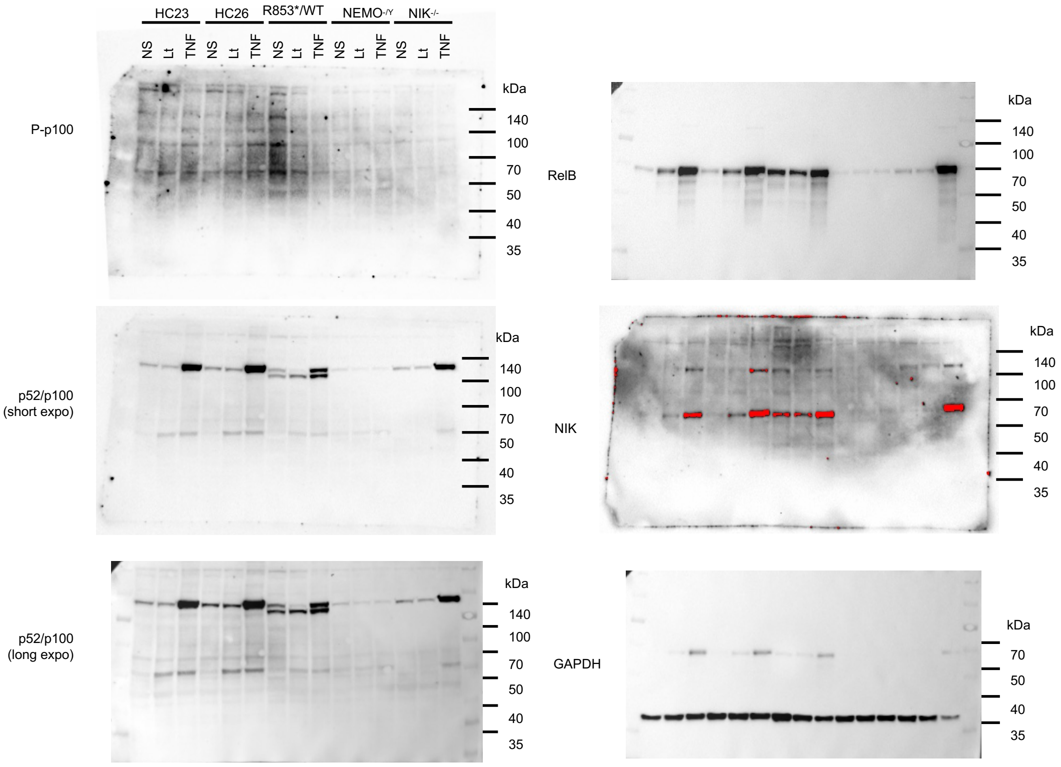

Supplementary Figure 5c
